# High diagnostic value of miRNAs for NSCLC: quantitative analysis for both single and combined miRNAs in lung cancer

**DOI:** 10.1080/07853890.2021.2000634

**Published:** 2021-12-16

**Authors:** Minhan Yi, Zexi Liao, Langmei Deng, Li Xu, Yun Tan, Kun Liu, Ziliang Chen, Yuan Zhang

**Affiliations:** a Department of Respiratory Medicine, Xiangya Hospital, Central South University, Changsha, Hunan, China; b School of Life Sciences, Central South University, Changsha, Hunan, China; c National Clinical Research Center for Geriatric Disorders, Xiangya Hospital, Central South University, Changsha, Hunan, China; d Xiangya Medical School, Central South University, Changsha, Hunan, China; e Department of Emergency, The Third Xiangya Hospital, Central South University, Changsha, Hunan, China; f School of Computer Science and Engineering, Central South University, Changsha, Hunan, China

**Keywords:** Lung cancer, microRNA, diagnostic biomarker, early diagnosis, NSCLC

## Abstract

**Background:**

MicroRNAs (miRNAs) are good candidates as biomarkers for Lung cancer (LC). The aim of this article is to figure out the diagnostic value of both single and combined miRNAs in LC.

**Methods:**

Normative meta-analysis was conducted based on PRISMA. We assessed the diagnostic value by calculating the combined sensitivity (Sen), specificity (Spe), positive likelihood ratio (PLR), negative likelihood ratio (NLR) and diagnostic odds ratio (DOR) and the area under the curve (AUC) of single and combined miRNAs for LC and specific subgroups.

**Results:**

A total of 80 qualified studies with a total of 8971 patients and 10758 controls were included. In non-small cell lung carcinoma (NSCLC), we involved 20 single-miRNAs and found their Sen, Spe and AUC ranged from 0.52-0.81, 0.66-0.88, and 0.68-0.90, respectively, specially, miR-19 with the maximum Sen, miR-20 and miR-10 with the highest Spe as well as miR-17 with the maximum AUC. Additionally, we detected miR-21 with the maximum Sen of 0.74 [95%CI: 0.62-0.83], miR-146 with the maximum Spe and AUC of 0.93 [95%CI: 0.79-0.98] and 0.89 [95%CI: 0.86-0.92] for early-stage NSCLC. We also identified the diagnostic power of available panel (miR-210, miR-31 and miR-21) for NSCLC with satisfying Sen, Spe and AUC of 0.82 [95%CI: 0.78-0.84], 0.87 [95%CI: 0.84-0.89] and 0.91 [95%CI: 0.88-0.93], and furtherly constructed 2 models for better diagnosis.

**Conclusions:**

We identified several single miRNAs and combined groups with high diagnostic power for NSCLC through pooled quantitative analysis, which shows that specific miRNAs are good biomarker candidates for NSCLC and further researches needed.

## 1. Introduction

Lung cancer (LC) is a type of malignant neoplasm arising from bronchial mucosa or glands which accounts for the largest proportion of cancer globally in consideration of patient quantity as well as mortality [1, 2]. Histologically, LC is categorized as small cell lung carcinoma (SCLC) and non-small cell lung carcinoma (NSCLC) which is further classified as adenocarcinoma (AD), squamous cell carcinoma (SCC) and large cell carcinoma (LCC) [3]. NSCLC, accounting for 80–85% of LC, harbours specific molecular and genetic characteristics [4], indicating the likelihood to distinguish NSCLC from other subtypes of LC under the help of particular biomarkers. We have identified the association between interleukin polymorphisms and protein levels with lung cancer susceptibility as well as phenotypes in our previous study [5]. Current challenges lying on its early diagnosis: lung tissue biopsy, being regarded as “gold standard”, has to be done through invasive bronchoscopy or surgical excision [4]; CT [6] as well as PET-CT [7] are widely applied in the definition of TNM stage in NSCLC, which still lacks specific diagnostic directivity towards NSCLC. Novel diagnostic methods such as the detection of circulating tumour cells or other circulating biomarkers [8] need further confirmation [9]. Among all possible biomarkers under research, microRNAs (miRNAs) are considered to be one of the most promising objects in terms of early detection of NSCLC [10].

MiRNAs are non-coding RNA with the length varied from 18 to 25 nucleotides who involve in the regulation of gene expression through suppression of mRNA directly. We previously proved that miRNAs could serve as diagnostic biomarker in asthma [11]. The abnormal expression level of multiple miRNAs has been determined among diverse cancers [12,13]: oncogenic miRNAs are the ones overexpressed inside tumour cells which promote the development and proliferation of cancerous cells; tumour-suppressive miRNAs are the ones who are down-regulated during the process of tumorigenesis. MiRNAs could be expelled from a tumour or stromal cells to the body or secreted fluid in the form of exosomes [14], providing the possibility for detection of exosomal miRNAs to be novel but useful approach of a cancer diagnosis.

Till now, a group of miRNAs has been proved to participate in LC cancerization, proliferation, and metastasis and their target genes have been confirmed [15]: miR-21 serves as oncogene and participates in multiple pathways controlling NSCLC tumorigenesis such as proliferation and angiogenesis [16]; miR-148a suppresses invasion of NSCLC cells by affecting Wnt1 pathway [17]; exosomal miR-619-5p improves angiogenesis as well as metastasis in NSCLC by inhibiting RCAN1.4 [18]. Remarkably, specific miRNAs like miR-590-5p and miR-26b possess potential to be diagnostic or prognostic biomarkers in NSCLC [19,20]. Specific miRNA panels even show their potential on histological subcategorization of NSCLC [10]: the concentration of miR-181b-5p, miR-30a-3p, miR-30e-3p, and miR-361-5p suggests higher possibility for AD while the combination of miR-10b-5p, miR-15b-5p, and miR-320b points to SCC. However, whether certain miRNA or miRNA panel could serve as good biomarkers candidates for NSCLC diagnosis or detection of early-stage NSCLC remains unknown.

Therefore, we aimed to clarify whether miRNAs, single miRNAs and miRNA panels, can serve as a biomarker for NSCLC and other LC subtypes by performing a quantitative analysis of previously published miRNA expression profiling studies? Did the results show any difference among various sample sources and clinical stages?

## 2. Materials and methods

### 2.1. Search strategy

The Preferred Reporting Items for Systematic Reviews and Meta-analyses (PRISMA) guidelines were followed [21]. We carefully search four databases (PubMed, Embase, Web of Science and Cochrane Library) by using keywords ((lung neoplasms) OR (lung tumour) OR (lung cancer) OR (lung carcinoma) OR (pulmonary neoplasms) OR (pulmonary cancer)) AND ((miRNA) OR (microRNA)) by Mar 31th, 2021. References and citing articles of the original articles were also searched artificially for further information extraction. All literatures were searched and screened by two independent staff because of objectivity principle. If there was a dispute, a third party was appreciated to make final decision.

### 2.2. Inclusion criteria based on PICOS

We strictly selected eligible studies by the principle of PICOS as follow:

1. Participants: patients attained the pathological diagnosis of LC who were further graded according to the 7th and 8th edition of lung cancer TNM grading by the International Association for the Study of Lung Cancer (including I, II, III, and IV) [22,23]. Available and detailed diagnosis of LC subtypes (including SCLC and NSCLC which further involved AD, SCC and LCC) defined by WHO classification in 2004 and 2015 [24,25] were appreciated.
2. Intervention: microarray or quantitative real-time polymerase chain reaction (qRT-PCR) for detection of the miRNAs’ expression levels in all participants;
3. Control: healthy people or cancer-free controls;
4. Outcomes: diagnostic data of individual miRNA or miRNA panels for lung cancer was provided, including true positive (TP), false positive (FP), false negative (FN), and true negative (TN);
5. Studies: case-control studies or cohort studies.

### 2.3. Exclusion criteria

We also carefully excluded articles with the following characteristics: (1) the literature not written in English; (2) researches not of the type as original articles, such as review, case report, letter, or conference summary; (3) the publication on the same topic from the same team which also shared overlapped participants;(4) deficiency of detailed data for combined analysis; (5) articles of each specific miRNA which owned less than 4 records; (6) articles of miRNA panels whose miRNA types were not all researched in our individual miRNA section.

### 2.4. Data extraction and quality assessment

We reviewed all the eligible publications and extracted the following information: the first author, year of publication, size of cases and controls, diagnosis of LC (including subtypes and staging), involved miRNAs and their expression outcome, as well as sources of the sample. We further normalized names of miRNAs from different studies basing on miRbase version 22 released in 2018 (http://www.mirbase.org/).

We used the Quality Assessment of Diagnostic Accuracy Studies-2 (QUADAS-2) [26], which consists of four key domains including patient selection, index test, reference standard, flow and timing, to evaluate the included papers by RevMan5.3 with the levels of “high”, “low” and “unclear”.

### 2.5. Statistical analysis

All the analyses were performed in STATA version 14 (Stata Corporation, College Station, TX, USA). The Spearman correlation coefficient was used to access the threshold effect, with *r* > 0.6 and *p* < .05, indicating a significant threshold effect between studies [27], which means effect size of included studies could be combined and further analyzed. The bivariate mixed‐effects mode [28] was used to calculate the indicators reflecting the diagnostic effect, such as sensitivity (Sen), specificity (Spe), positive likelihood ratio (PLR), negative likelihood ratio (NLR), diagnostic odds ratio (DOR) and corresponding 95% credible interval (CI). The summary receiver operator characteristic curve (SROC) which was plotted based on Sen and Spe and the area under the SROC curve (AUC) was also used to test the pooled diagnostic value of miRNAs.

Heterogeneity between studies was evaluated by the *I*^2^ test, with *I*^2^>25% indicating heterogeneity [29]. When the *I*^2^>50%, it suggested the heterogeneity between studies was high [30]. Then the subgroup and meta-analysis would be performed to find the sources of heterogeneity, which we performed from the aspect of sample sources (respiratory-based sample vs. blood-based sample) and control group (health and cancer-free).

We also performed the sensitivity analysis for further analysis of those studies that resulting in large heterogeneity [31]. By comparing the changes in effect size and 95% confidence intervals before and after the inclusion and exclusion of those studies, we accessed the impact of those studies on the process of pool analysis.

We used two methods, the Deek and the Funnel plot [32], to evaluate the publication bias based on the situation of a different number of studies in various miRNAs. When the number of studies more than 10, the Deek method was adopted, otherwise, the Funnel plot method was used. When the *p* > .05 in Deek or the Funnel plot is symmetrical, it suggests that there may be no obvious publication bias. Otherwise, the trim and fill method [33] will be used to review and identify the publication bias.

## 3. Results

### 3.1. Characteristics of the included studies and quality assessment

**Figure 1.**
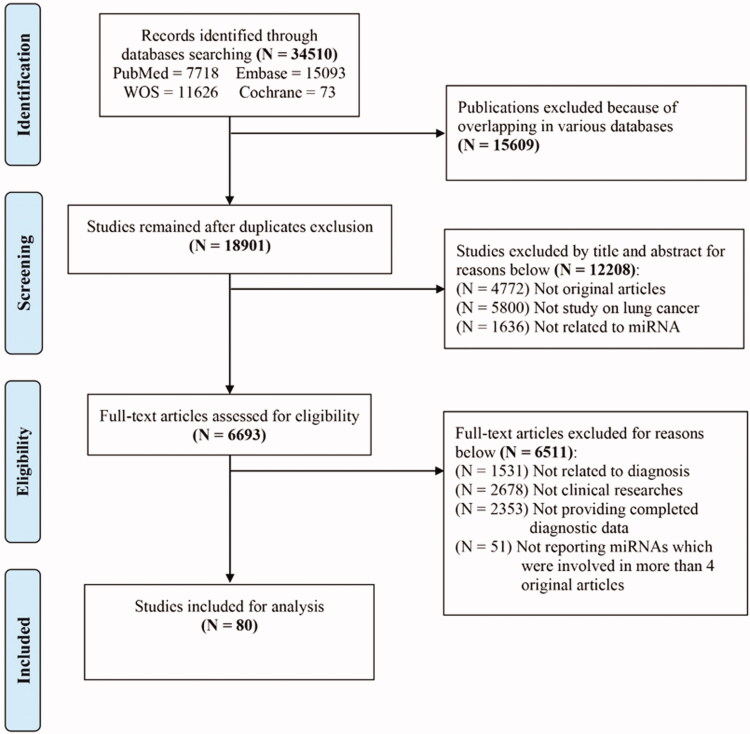
Flowchart of study selection based on the inclusion and exclusion criteria.

Based on the listed criteria of inclusion and exclusion in Method and PRISMA standard, a total of 80 studies with 8971 patients and 10758 controls were eventually included (Figure 1), among which, 24 single-miRNAs (miR-21, -210, -145, -155, -486, -17, -126, -223, -31, -20, -182, -146, -205, -19, -221, -200, Let-7, -125, -7, -10, -375, -150, -92, -25) were involved as reported in more than 4 articles in LC. Besides, a variety of panels with several miRNAs were also reported. Based on the QUADAS-2, these researches’ quality was at the middle and upper grades (Supplementary Appendix Figure 1). All involved original article harboured ethical approval.

**Figure 2.**
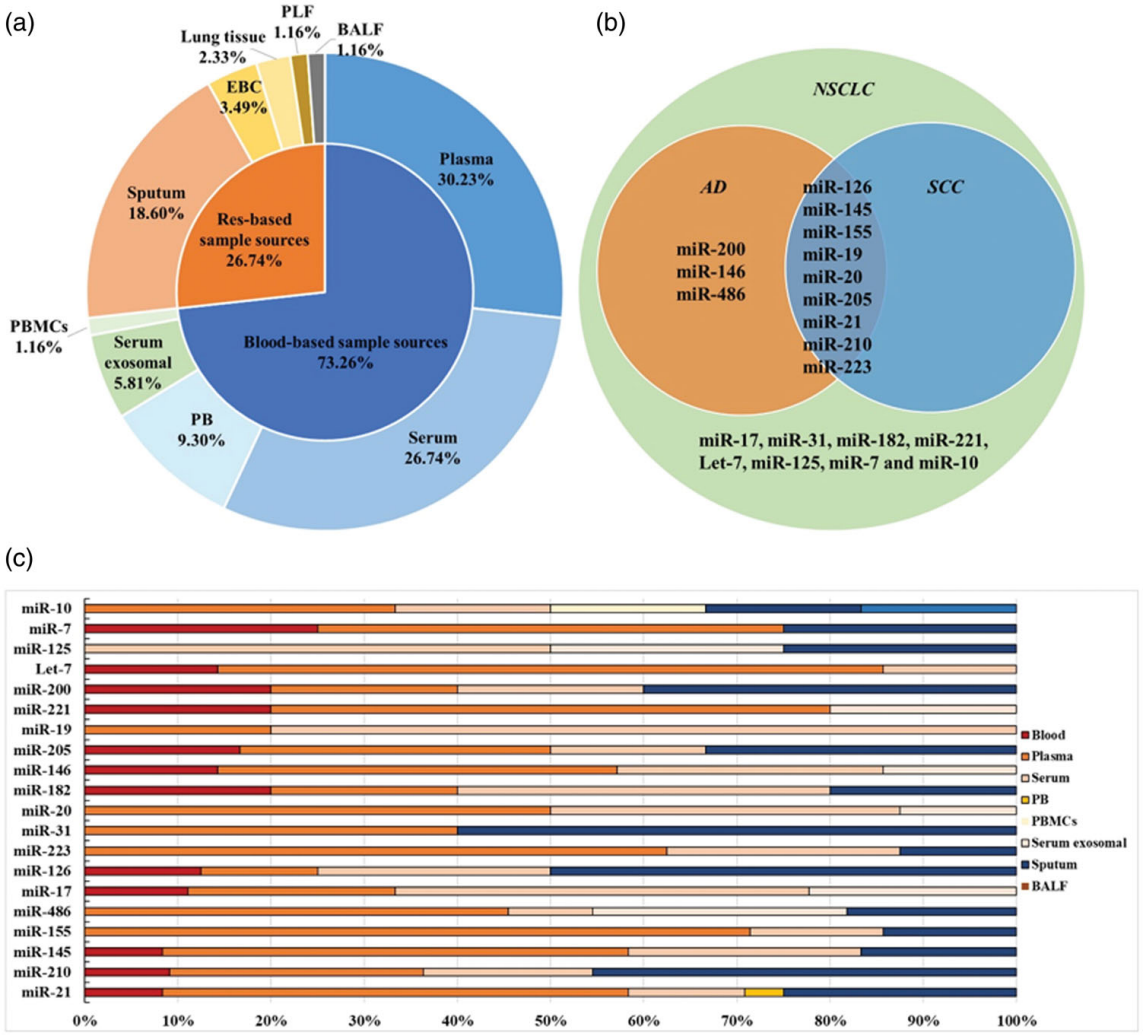
The characteristics for included miRNAs of involved articles. (a) The proportion of sample sources for researched single-miRNAs and miRNA panels miRNAs in all included articles. (b)The relationship between total reported miRNAs and LC subtypes. (c) The proportion of specimen sources among 20 included single-miRNAs. Tumour type: AD: adenocarcinoma; SCC: squamous cell carcinoma; SCLC: small cell lung carcinoma; NSCLC: non-small cell lung carcinoma. Sample sources: EBC: exhaled breath condensate; PB: peripheral blood; PBMCs: peripheral blood mononuclear cells; BALF: bronchoalveolar lavage; PLF: pleural lavage fluid.

For all included researches (Table 1), the major LC subtype involved was NSCLC (69 studies, 86.3%), of which AD (13 studies) furtherly occupied the majority and SCC (10 studies) following after, while SCLC accounted for the least (1 study, 1.25%) and the rest (10 studies, 12.5%) lacking of clear description of LC subtypes. Additionally, except 15 articles (18.8%) without description of staging, we noticed that there were 44 studies (55%) stated I–IV stages, 17 studies (21.3%) focussed on early stage (I–II) and 4 studies (3.8%) concentrated on stage of III–IV. Furthermore, considering sample sources of researched miRNAs, blood derived samples sources (peripheral whole blood, serum, plasma or specific ingredients of peripheral blood mononuclear cell or serum exosomal) accounted for the main sources (73.26%) while respiratory system derived samples sources occupied the proportion of 26.74%, which included sputum, pleural lavage fluid, lung tissue, bronchoalveolar lavage fluid and exhaled breath condensate (Figure 2(a)). Therefore, we took these features, including LC subtypes, clinical staging and sample sources, into consideration in subgroup analysis.

**Table 1.** The main features of eligible studies that related to the diagnosis of miRNA for LC.

Study ID	Region	NO. of Case/Control	Tumor-type	Stage*	Sample sources	PMID
2020.Zhang, Z. J. [34]	China	330/312	NSCLC	I-IV	Serum exosomal	33178588
2020.Yang, C. [35]	China	74/23	NSCLC	III-IV	Serum	32466856
2020.Wu, Q. [36]	USA	48/48	NSCLC	I-II	Serum exosomal	32021461
2020.Wang, W. [37]	China	54/28	NSCLC	I	Blood	32596148
2020.Wang, J. Y. [38]	China	82/90	AD	I-IV	Serum	32388809
2020.Liu, X. [39]	China	245/245	NSCLC	I-IV	Serum	31906699
2020.Liu, C. [40]	USA	64/15	NSCLC	I-II	Serum exosomal	32265989
2020.Liao, J. [41]	USA	132/127	NSCLC	I-IV	Sputum, Plasma	31994346
2020.Ghany, S. M. A. [42]	Egypt	70/34	NSCLC	NA	Plasma	NA
2020.Fehlmann, T. [43]	USA	606/2440	LC	I-IV	Blood	32134442
2020.Asakura, K. [44]	Japan	1566/2178	LC	I-IV	Serum	32193503
2019.Zou, J. G. [45]	China	50/30	NSCLC	NA	Serum	30779079
2019.Zhang, Y. [46]	China	172/137	NSCLC	I-III	Serum exosomal	31146974
2019.Xi, K. [47]	China	67/25	NSCLC	I-II	Plasma	31632908
2019.Wang, S. [48]	China	50/24	NSCLC	NA	Plasma	31049003
2019.Szczyrek, M. [49]	Poland	160/45	NSCLC	I-IV	Plasma	31115013
2019.Switlik, W. Z. [50]	Poland	14/29	AD	NA	Serum	30950648
2019.Sui, A. [51]	China	76/60	NSCLC	I-IV	Serum	30867756
2019.Sheervalilou, R. [52]	Iran	47/41	NSCLC	I-IV	Plasma	32215263
2019.Roman-Canal, B. [53]	Spain	14/21	LC	NA	PLF	31636323
2019.Li, J. [54]	China	471/489	AD, SCC	I-IV	Plasma	31674214
2019.Hetta, H. F. [55]	USA	40/20	NSCLC	I-IV	Plasma	31244320
2019.Abdollahi, A. [56]	Iran	43/43	NSCLC	I-IV	Blood	31236600
2018.Yang, Y. L. [57]	China	194/199	NSCLC	III-IV	PBMCs	30064233
2018.Yang, Y. [58]	China	104/50	NSCLC	I-IV	Serum	29430184
2018.Xi, K. X. [59]	China	42/15	NSCLC	I-II	Plasma	30174846
2018.Sun, Y. [60]	China	28/28	AD	I-IV	Plasma	29103767
2018.Qiu, F. [61]	China	58/42	LC	NA	PB	30556877
2018.Poroyko, V. [62]	USA	20/10	LC	NA	Serum exosomal	29731983
2018.Mohamed, M. A. [63]	Egypt	50/50	LC	I-IV	Lung tissue	29437031
2018.Leng, Q. [64]	USA	56/28	NSCLC, SCC, AD	I-IV	Plasma	29783093
2018.Fan, L. H. [65]	China	128/79	NSCLC	I	Serum	NA
2018.Bao, M. [66]	China	80/75	NSCLC	I-IV	Serum, Lung tissue	31938164
2018.Bagheri, A. [67]	Iran	30/30	NSCLC, SCC, AD	I-IV	Sputum	30485511
2018.Aiso, T. [68]	Japan	56/26	NSCLC	I-IV	Serum	30405804
2018.Abu-Duhier, F.M. [69]	Saudi Arabia	80/80	AD, SCC	I-IV	Plasma	30256067
2017.Zhang, H. [70]	China	129/83	NSCLC	I-II	Plasma	28356944
2017.Yu, Y. [71]	USA	50/30	SCLC	I-IV	Plasma	28106539
2017.Sheervalilou, R. [72]	Iran	30/30	NSCLC	I-III	BALF, Sputum	NA
2017.Shang, A. Q. [73]	China	127/112	NSCLC	I-IV	Serum	28253725
2017.Lv, S. [74]	China	160/160	AD	I-IV	Serum	28123597
2017.Leng, Q. [75]	USA	126/118	NSCLC	I-IV	Plasma	29340099
2017.Ibrahim, F. K. [76]	Egypt	15/15	LC	NA	EBC	NA
2017.Bagheri, A. [77]	Iran	17/17	NSCLC	I-IV	Sputum, Lung tissue	29090068
2016.Zhu, W. [78]	China	112/104	NSCLC	I-II	Serum	27093275
2016.Zaporozhchenko, I.A. [79]	Russia	75/50	SCC, AD	II-IV	Plasma	27768748
2016.Wang, X. [80]	China	59/59	NSCLC	I-IV	Plasma	27499953
2016.Su, Y^b^. [81]	USA	57/62	NSCLC	I	Sputum	27777637
2016.Su, Y^a^. [82]	USA	117/174	NSCLC	I	Sputum	27176474
2016.Razzak, R. [83]	Canada	43/10	NSCLC	I-II	Sputum	27122989
2016.Jia, Y. C. [84]	China	35/30	NSCLC, SCLC	NA	Plasma	NA
2016.Chen, J. L. [85]	China	30/30	NSCLC	I-II	EBC, Serum	NA
2015.Zhao, W. [86]	China	80/60	NSCLC	NA	Serum	26628958
2015.Yang, J. S. [87]	China	152/300	NSCLC	I-IV	Serum	25501703
2015.Yan, H. J. [88]	China	300/300	NSCLC	I-IV	PB	25765717
2015.Xing L. [89]	USA	203/227	LC	I-II	Sputum	25593345
2015.Wang, R. J. [90]	China	70/70	NSCLC	NA	Serum	25755772
2015.Wang, P. [91]	China	142/111	NSCLC	I-II	Serum	25639977
2015.Su, J. [92]	USA	56/73	NSCLC	NA	Sputum	26309391
2015.Li, W. [93]	China	11/11	NSCLC, SCC	NA	Plasma	26237047
2015.Fan, L. [94]	China	164/112	NSCLC	I-III	Serum	26695145
2015.Dou, H. [95]	China	120/360	NSCLC	I-IV	Plasma	26309587
2014.Zhu, W. Y. [96]	China	36/44	NSCLC	I	Serum	24945821
2014.Li, N. [97]	USA	35/40	NSCLC	NA	Sputum	24281335
2014.Geng, Q. [98]	China	151/85	NSCLC, SCC, AD	I-II	Plasma	25421010
2013.Zeng, X. L. [99]	China	64/26	NSCLC	I-IV	PBMCs	24286416
2013.Tang, D. [100]	China	96/92	NSCLC, SCC, AD	I-III	Plasma	23462458
2013.Mozzoni, P. [101]	Italy	54/46	NSCLC	I-III	Plasma, EBC	24102090
2013.Anjuman, N. [102]	USA	43/47	NSCLC	I	Sputum	24053570
2013.Abd El Fattah, A. A. [103]	Egypt	65/37	LC	NA	Serum	23559272
2012.Wang, B. [104]	China	31/39	LC	I-IV	Serum	22638884
2011.Wei, J^b^. [105]	China	77/36	NSCLC	I-IV	Plasma	21627863
2011.Wei, J^a^. [106]	China	63/30	NSCLC	I-IV	Plasma	23483517
2011.Shen J^b^. [107]	USA	58/29	NSCLC, SCC, AD	III-IV	Sputum	21116241
2011.Shen J^a^. [108]	USA	108/113	NSCLC	I-IV	Sputum, Plasma	21864403
2011.Li, Y. [109]	China	20/10	NSCLC	I-IV	PB	22866162
2011.Jeong, H. C. [110]	Korea	35/30	NSCLC	I-IV	Blood	21468581
2010.Yu, L. [111]	USA	64/58	NSCLC, AD	I-IV	Sputum	21351266
2010.Xing, L. [112]	USA	67/55	SCC	III-IV	Sputum	20526284
2010.Xie, Y. [113]	USA	23/17	NSCLC	I-IV	Sputum	19446359

^a,b^: Different articles by the same author initials in the same year. *: The stage of clinical diagnosis in the CASE group, in which stage I-IV represents patients who are not classified as stage I-II or III-IV, including II-III, I-III, I-IV. NA: not available.

Tumor type: AD: Adenocarcinoma; SCC: Squamous cell carcinoma; SCLC: Small cell lung carcinoma; NSCLC: Non-Small Cell Lung Carcinoma; LC: lung cancer.

Sample sources: EBC: exhaled breath condensate; PB: Peripheral blood; PBMCs: Peripheral blood mononuclear cells; BALF: bronchoalveolar lavage; PLF: Pleural lavage fluid.

Considering that specified diagnosis of LC histologically usually provides clinical value for the selection of therapy, we intended to identify specific miRNAs connected with LC subtypes. Since NSCLC occupied the major histological subtype of LC, here we specially focus on the diagnostic potential of 20 miRNAs being involved in NSCLC (miR-21, -210, -145, -155, -486, -17, -126, -223, -31, -20, -182, -146, -205, -19, -221, -200, Let-7, -125, -7 and -10). When analyzing the associations between researched 20 miRNAs and NSCLC subtypes, we further found 9 miRNAs reported in both AD and SCC. Besides, 3 kinds miRNAs (miR-200, −146 and −486) were specifically reported in AD (Figure 2(b)). The proportion of specimen sources among theses 20 miRNAs were diagrammatically shown in Figure 2(c). In addition, due to data limitations, the diagnostic value of the other 4 miRNAs (miR-375, -150, -92 and -25) could only be explored in LC rather than NSCLC.

### 3.2. Single miRNA as a diagnostic biomarker in NSCLC

#### 3.2.1. The diagnostic value of 20 miRNAs singlely in NSCLC

We analyzed 20 single-miRNAs mentioned above (Supplementary Appendix Table 1) and found there was no significant statistical threshold effect among involved miRNAs according to Spearman (Supplementary Appendix Table 2). Besides, slight heterogeneity as well as publication bias which could be corrected were observed, indicating the rationality of pooled analysis respectively (Supplementary Appendix Figure 2, Supplementary Appendix Table 3). As for the other 4 miRNAs (miR-375, -150, -92 and -25) in the unclassified LC, they are similar to the above 20 miRNAs and the diagnostic data and results are shown in the (Supplementary Appendix Table 4–6, Supplementary Appendix Figure 3–4).

Through pooled effect analysis on these 20 miRNAs, their Sen, Spe, and AUC varied from 0.52-0.81, 0.66-0.88 and 0.68-0.90, respectively. According to our results, miR-19 had the highest sensitivity of 0.81 [95%CI: 0.70-0.89], 2 miRNAs (miR-20 and miR-10) both shown the best specificity of 0.88 and miR-17 owned the highest AUC value of 0.90 [95%CI: 0.87-0.92]. Additionally, there were 12 miRNAs with AUC equal or higher than 0.8, 4 miRNAs with AUC equal or higher than 0.85, which suggested that these miRNAs were more worthy of attention in NSCLC diagnosis than other miRNAs. (Table 2, Supplementary Appendix Figure 3–4). In addition, we identified miR-21 could even be a potential satisfying biomarker for AD diagnosis considering its favoured sensitivity of 0.72 [95%CI: 0.57-0.83], specificity of 0.70 [95%CI: 0.46-0.87] and AUC of 0.76 [95%CI: 0.72-0.80].

**Table 2.** Detailed assessment of overall diagnostic value of 20 single-miRNAs in NSCLC.

miRNA-type	No. of research (Case/Control)	Sen [95% CI]	Spe [95% CI]	PLR [95% CI]	NLR [95% CI]	AUC [95% CI]	DOR [95% CI]
miR-21	23 (1641/1503)	0.70 [0.64–0.76]	0.77 [0.73–0.81]	3.1 [2.6–3.6]	0.39 [0.32–0.46]	0.81 [0.77–0.84]	8 [6–11]
miR-210	11 (670/645)	0.71 [0.57–0.82]	0.81 [0.70–0.88]	3.7 [2.6–5.3]	0.36 [0.26–0.51]	0.83 [0.79–0.86]	10 [7–15]
miR-145	11 (762/626)	0.73 [0.57–0.84]	0.75 [0.66–0.82]	2.9 [2.1–4.0]	0.36 [0.22–0.58]	0.80 [0.76–0.83]	8 [4–16]
miR-155	10 (697/555)	0.77 [0.63–0.87]	0.84 [0.72–0.91]	4.8 [2.6–8.9]	0.27 [0.16–0.46]	0.88 [0.85–0.91]	18 [7–47]
miR-486	10 (491/404)	0.75 [0.71–0.78]	0.76 [0.68–0.82]	3.1 [2.3–4.2]	0.33 [0.28–0.39]	0.77 [0.73–0.80]	9 [6–14]
miR-17	9 (1339/1170)	0.78 [0.63–0.88]	0.86 [0.77–0.92]	5.7 [3.1–10.3]	0.26 [0.15–0.46]	0.90 [0.87–0.92]	22 [8–60]
miR-126	8 (531/430)	0.70 [0.51–0.84]	0.85 [0.76–0.91]	4.6 [2.8–7.6]	0.36 [0.21–0.62]	0.86 [0.83–0.89]	13 [5–31]
miR-223	7 (605/419)	0.78 [0.73–0.83]	0.81 [0.72–0.87]	4.0 [2.7–6.0]	0.27 [0.21–0.35]	0.85 [0.82–0.88]	15 [8–27]
miR-31	7 (873/819)	0.73 [0.63–0.81]	0.78 [0.74–0.81]	3.3 [2.8–3.8]	0.35 [0.25–0.48]	0.80 [0.77–0.84]	9 [6–14]
miR-20	7 (329/239)	0.68 [0.47–0.84]	0.88 [0.72–0.95]	5.6 [2.6–12.2]	0.36 [0.21–0.62]	0.86 [0.83–0.89]	15 [6–37]
miR-182	6 (444/227)	0.57 [0.43–0.70]	0.79 [0.61–0.90]	2.7 [1.5–4.9]	0.54 [0.42–0.70]	0.72 [0.68–0.76]	5 [2–10]
miR-146	6 (306/247)	0.58 [0.36–0.76]	0.86 [0.62–0.96]	4.0 [1.6–10.0]	0.49 [0.33–0.74]	0.78 [0.75–0.82]	8 [3–22]
miR-205	6 (332/270)	0.57 [0.43–0.69]	0.78 [0.53–0.92]	2.6 [1.2–5.7]	0.55 [0.44–0.69]	0.68 [0.64–0.72]	5 [2–11]
miR-19	6 (470/363)	0.81 [0.70–0.89]	0.66 [0.53–0.77]	2.4 [1.7–3.4]	0.29 [0.18–0.45]	0.81 [0.77–0.84]	8 [4–16]
miR-221	6 (283/176)	0.52 [0.28–0.74]	0.75 [0.60–0.86]	2.1 [1.2–3.8]	0.64 [0.39–1.04]	0.73 [0.68–0.76]	3 [1–9]
miR-200	5 (200/154)	0.58 [0.48–0.67]	0.74 [0.60–0.84]	2.2 [1.5–3.4]	0.57 [0.46–0.70]	0.69 [0.64–0.73]	4 [2–7]
Let-7	5 (297/486)	0.67 [0.57–0.75]	0.74 [0.65–0.81]	2.5 [1.7–3.9]	0.45 [0.32–0.65]	0.76 [0.72–0.80]	6 [3–12]
miR-125	4 (466/456)	0.67 [0.57–0.75]	0.66 [0.57–0.73]	1.9 [1.6–2.3]	0.51 [0.41–0.62]	0.71 [0.67–0.75]	4 [3–5]
miR-7	4 (168/86)	0.66 [0.44–0.83]	0.86 [0.66–0.95]	4.8 [2.1–10.6]	0.39 [0.23–0.66]	0.85 [0.81–0.88]	12 [5–28]
miR-10	4 (351/345)	0.79 [0.72–0.85]	0.88 [0.73–0.96]	6.8 [2.6–17.8]	0.24 [0.16–0.34]	0.85 [0.82–0.88]	29 [8–103]

Sen: sensitivity; Spe: specificity; PLR: positive likelihood ratio; NLR: negative likelihood ratio; AUC: area under curve; DOR: diagnostic odds ratio; CI: confidence interval.

**Figure 3.**
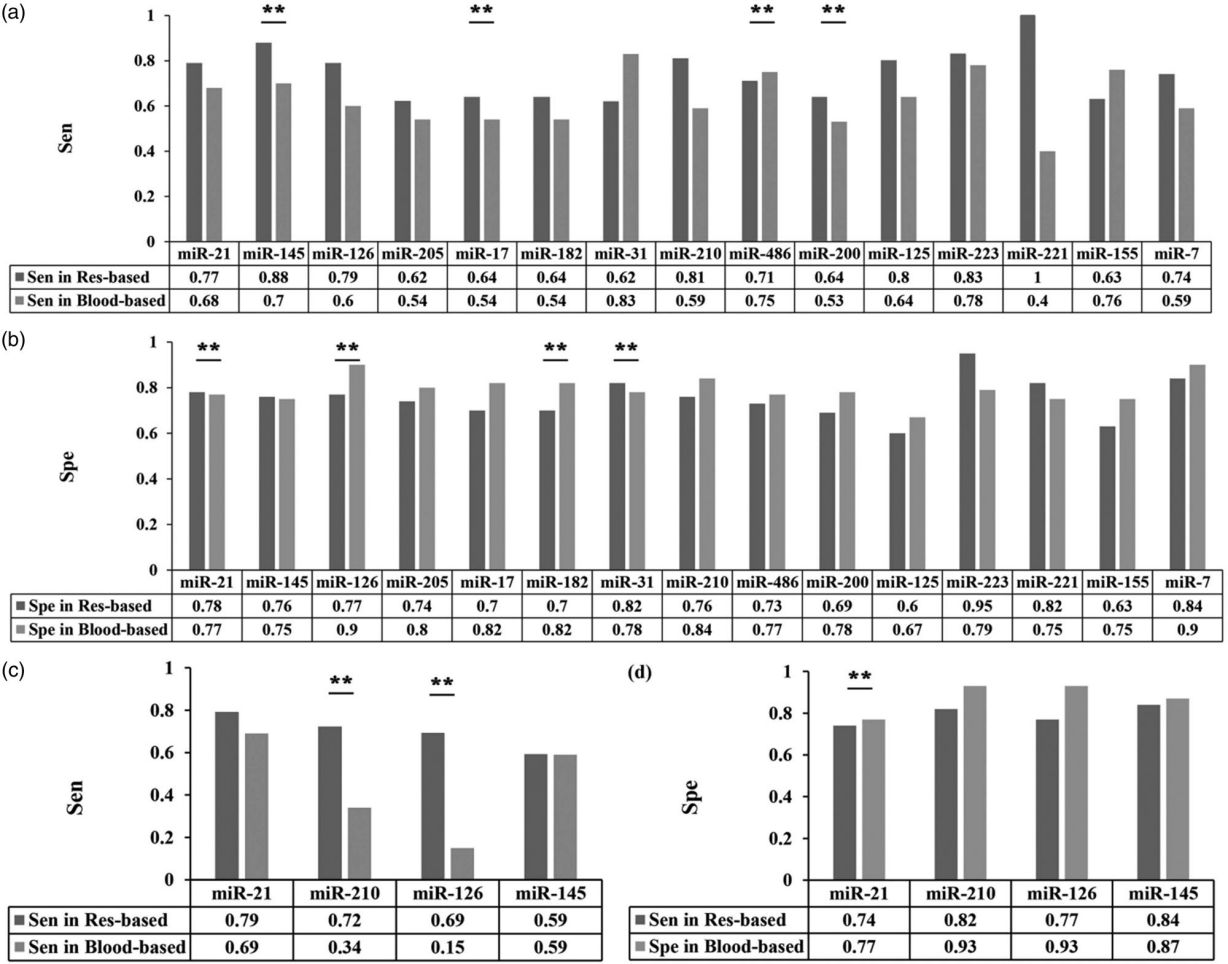
The diagnostic value of several single-miRNAs in NSCLC between different sample sources. (a–d) The difference in diagnostic power of a certain miRNA between respiratory-based and blood-based samples for NSCLC (a–b) and early NSCLC (c–d) through comparison of sensitivity (a, c) and specificity (b, d). Colour in dark gray refers to respiratory-based samples while colour in light gray refers to blood-based samples.

We further conducted subgroup analysis according to sample sources. We found that miR-145 had the most obvious difference in sensitivity (0.88 [95%CI: 0.68–1.00] vs. 0.70 [95%CI: 0.54–0.86]) (Figure 3(a)) and miR-126 showed the largest difference in specificity between the two kinds of samples (0.77 [95%CI: 0.44–0.82] vs. 0.90 [95%CI: 0.68–0.82]). (Figure 3(b)). These results with obvious otherness suggested different selection of miRNAs as biomarkers, depending on sample sources.

#### 3.2.2. The diagnostic value of 5 miRNAs singlely in early NSCLC

Since early diagnosing (stage I and II) of NSCLC help to reach better prognosis in NSCLC, we conducted analysis on assessment of diagnostic efficiency for miRNAs in early NSCLC. We further found 5 miRNAs (miR-21, -145, -126, -210, and -146) from above 20 miRNAs possessing diagnostic value for early lung cancer with Sen, Spe, and AUC varying from 0.49 to 0.74, 0.76 to 0.93, and 0.80 to 0.89, respectively. Noteworthily, miR-21 and miR-146, owned the highest value of Sen (0.74 [95%CI: 0.62–0.83]) and Spe (0.93 [95%CI: 0.79–0.98]) respectively. As for best performance assessing by AUC, miR-146 owned the highest value of 0.84, indicating them as candidate markers of early diagnosis of NSCLC (Table 3, Supplementary Appendix Figure 7–8).

**Table 3.** The overall diagnostic value of single miRNAs in early NSCLC.

miRNA-type	No. of research (Case/Control)	Sen [95% CI]	Spe [95% CI]	PLR [95% CI]	NLR [95% CI]	AUC [95% CI]	DOR [95% CI]
miR-21	10 (703/606)	0.74 [0.62–0.83]	0.76 [0.68–0.82]	3.1 [2.4–3.9]	0.35 [0.25–0.49]	0.81 [0.78–0.84]	9 [6–13]
miR-145	4 (342/260)	0.59 [0.34–0.80]	0.86 [0.68–0.95]	4.2 [2.0–8.8]	0.48 [0.28–0.82]	0.83 [0.79–0.86]	9 [3–23]
miR-126	4 (170/160)	0.56 [0.30–0.79]	0.81 [0.70–0.89]	2.9 [1.9–4.5]	0.55 [0.32–0.95]	0.80 [0.77–0.84]	5 [2–13]
miR-210	4 (251/298)	0.64 [0.46–0.78]	0.83 [0.76–0.88]	3.8 [2.6–5.5]	0.44 [0.28–0.68]	0.84 [0.81–0.87]	9 [4–18]
miR-146	4 (144/89)	0.49 [0.25–0.75]	0.93 [0.79–0.98]	7.3 [2.6–20.2]	0.54 [0.33–0.90]	0.89 [0.86–0.92]	13 [4–43]

Sen: sensitivity; Spe: specificity; PLR: positive likelihood ratio; NLR: negative likelihood ratio; AUC: area under curve; DOR: diagnostic odds ratio; CI: confidence interval.

Considering the subtype-analysis of different sampling sources, we analyzed 4 of 5 single-miRNAs since miR-146 was merely available in blood-based sample. According to our results, miR-210 (0.72 [95%CI: 0.64-0.80] vs. 0.34 [95%CI: 0.14-0.55]) as well as miR-126 (0.69 [95%CI: 0.61-0.78] vs. 0.15 [95%CI: 0.01-0.30]) shown superiority in respiratory-based samples compared with blood-based samples in the aspect of sensitivity (Figure 3(c)). When it came to calculation of pooled specificity, only miR-21 occupied statistical difference. Still, their results for specificity appeared to be similar in the above two sampling sources (0.77 [95%CI: 0.68-0.87] vs. 0.74 [95%CI: 0.63-0.84], Figure 3(d)). Therefore, we supposed the diagnostic value on early NSCLC of miR-210 and miR-126 depended partly on their sample sources.

### 3.3. Combining miRNAs into panels as diagnostic biomarker in NSCLC

**Figure 4.**
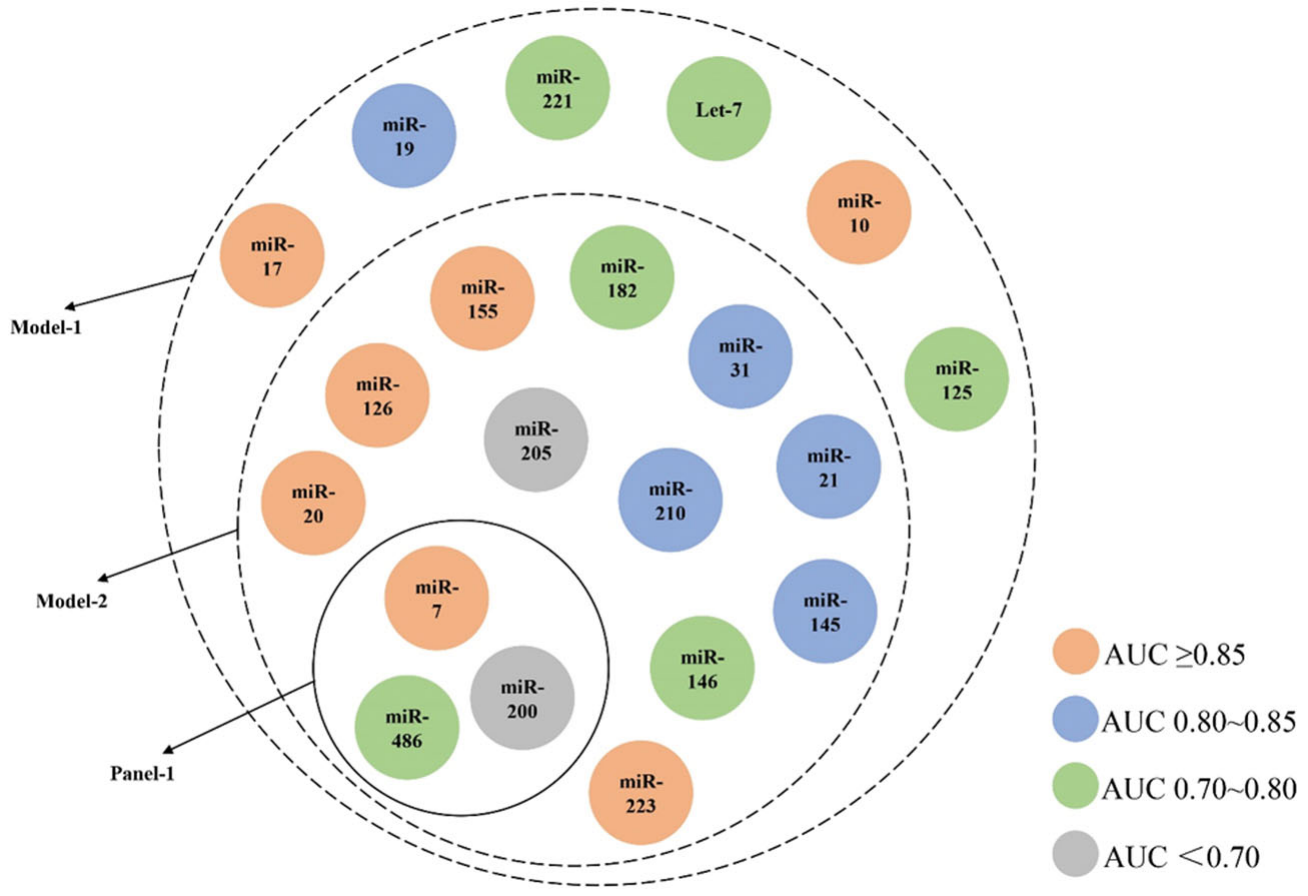
Containment relationship of analyzed single-miRNAs as different combined groups for NSCLC. The colour of each miRNA depends on their AUC value shown in Table 2. Panel-1contains three miRNAs (miR-21, -31, -210), Model-1 contains 20 miRNAs and Model-2 contains 14 miRNAs.

According to our results above, the performance of a certain miRNA seemed to reach satisfaction considering either sensitivity or specificity, making it difficult to choose one particularly for NSCLC diagnosis. For investigation of NSCLC diagnostic value of combining miRNAs, we firstly conducted pooled effect size analysis on available miRNA-panels (involving multiple miRNAs researched at the same time in participants) which were reported in no less than 4 articles and uniquely contained miRNAs as mentioned in above section (Supplementary Appendix Table 7). To sum up, there were only 1 panel (Panel-1) fulfilling the above criteria, containing miR-210, miR-31 and miR-21 for NSCLC (Figure 4). Still, it performed well according to AUC (0.91 [95%CI: 0.88-0.93]) and even better than any single miRNAs in both Sen and Spe of 0.82 [95%CI: 0.78-0.84] and 0.87 [95%CI: 0.84-0.89] respectively (>0.8), suggesting enhanced diagnostic value in NSCLC of combining miRNAs as panels.

Additionally, there were also other miRNA-panels only containing above 20 miRNAs but reported in less than 4 articles (Supplementary Appendix Table 7).For further verification of these favourable miRNAs, we conducted effect size analysis by constructing models consisting of numerous reported panels. During this process, there was no requirement of the publication count for each panel except that the components of miRNA types are among the 20 miRNAs mentioned in previous section. The positive outcome of these models was defined as either of the involved panels owned positive finding. We firstly constructed Model-1 that finely retained these 20 miRNAs. As for total NSCLC, Model-1, the combination of 20 miRNAs, illustrated much better efficiency compared to any single miRNA with Sen of 0.77 [95%CI: 0.74-0.80], Spe of 0.84 [95%CI: 0.82-0.86] and AUC of 0.88 [95%CI: 0.85-0.91] since no miRNA could achieve such satisfying sensitivity and specificity at the same time. (Figure 4, Supplementary Appendix Figure 9, 10). Still, considering the large quantity of involved miRNAs, we furtherly set up Model-2 by limiting AUC cut-off value of each involved panel as no less than 0.80 to optimise the best combination of miRNA for construction of diagnostic panels with less miRNAs while owning better diagnostic value (with the sensitivity of 0.79 [95%CI: 0.75-0.82], specificity of 0.86 [95%CI: 0.83–0.88] and AUC value of 0.90 [95%CI: 0.87-0.92]). All the results above suggested the diagnostic potential of Model-1 and -2 in clinical practice.

## 4. Discussion

In this pooled study, we firstly quantificationally assessed the diagnostic value of 20 single-miRNAs for NSCLC and furtherly conducted subgroup analysis on sample sources. We found that, according to the cut-off of AUC > 0.85, there were 4 single-miRNAs ranked top, containing miR-155, miR-17, miR-126 and miR-20 for NSCLC. Additionally, miR-21 and miR-146 also shown promising diagnostic value for early-stage NSCLC. Besides, we also found that miRNA panel which contains miR-210, miR-31 and miR-21 illustrated much better efficiency compared to any single-miRNA and constructed 2 models for optimization. All our results suggested that single-miRNAs and miRNA panels show high diagnostic values and are good diagnosis biomarker for NSCLC.

Although there are plenty of original articles on the diagnostic function of miRNAs, meta-analysis of single-miRNA and their potential for LC diagnosis is limited in quantity. A current published systemic review [114] discussed the diagnostic and prognostic potential of miRNAs in LC. Still, they focussed on the changing trend of miRNA expression and carried out a criterion to grade miRNAs for screening of candidate in LC diagnosis while we directly obtained potential biomarkers including single-miRNAs and miRNA panels through quantitative analysis. On the other hand, they found 7 miRNAs (miR-21, miR-25, miR-27b, miR-19b, miR-125b, miR-146a, and miR-210) with talent as latent therapic targets for LC, 5 of which were also included in our analysis (miR-21, miR-19, miR-125, miR-146 and miR-210). Furtherly, we identified miR-19 with maximum sensitivity in LC, miR-146 with maximum specificity in LC, miR-21 with maximum sensitivity in early-stage LC and miR-210 with maximum AUC value in early-stage LC. Therefore, our study not only serve as quantitative evidence supporting the former review, but also intuitively illustrated specific diagnostic power of each single-miRNAs, miRNA panels and novel models, which provides more convincible testimony for clinical practice of miRNAs as NSCLC biomarkers.

There are many miRNAs, e.g. miR-17 [115,116] and miR-223 [117], with good diagnostic performance also shown related biological functions. However, some of the miRNAs who also contribute to LC neoplasia fail to serve as candidate for LC diagnosis according to our results. Members in miR-200 family participate in a series of LC-related function such as migration, invasion and mesenchymal-to-epithelial transition [118]. Still, their diagnostic power was less than satisfactory. MiR-375 was considered to associated with NSCLC development and Cladin-1 was proved to be its target [119], while whether miR-375 could be suitable diagnostic biomarker of LC is lacking of evidence. This situation suggested that there may be candidate miRNAs did not included in our current research limited by our strict inclusion criteria, and high quality study screened for a wider group of miRNAs are need.

A hallmark of early-stage LC is speculated for a long time in practice. Since extracellular secretion of miRNAs were observed during the process of cancerization in LC [120], the expression levels of some miRNAs might be lower at the beginning of LC tumorigenesis in samples sourced from body fluid (like serum) or secreta (like sputum). In this article, we defined early-stage LC as stage I or II after considering former related articles [121] and in view of different treatment strategies recommended for each stage [122]. According to our data, we found the expression level of miR-145 and miR-486 vary during different stages of LC regardless of specific LC subtypes: the expression of miR-145 [37,51,59,67,68,70,90,98,100,111] and miR-486 [36,40,63,80,84,89,101,107] was upregulated in LC at stage I–II while that was downregulated after the studied stages covered from I to IV. Considering the anti-tumour effect of miR-145 and miR-486 [123–126], we supposed that the tumour itself might tend to reduce intracellular miR-145 and miR-486 level for its good growth. Still, the underlying specific mechanism of this interesting finding needs further study because we did not conduct a subgroup analysis of confounding factors like LC subtypes or sample sources considering limited information provided by original articles.

According to our study, blood is a good sample source in the case of detecting miRNAs as diagnostic marker for LC. Since LC rooted in the normal respiratory tract, we supposed that samples from the respiratory system might be more powerful subjects than blood-derived samples. Detection of LC by sputum cytology was not satisfying according to earlier studies [127,128]. What’s more, articles using other respiratory samples have limitations not only on quantity but also on biomarkers researchers chose to detect (most of them focus on DNA, cytokines, and other proteins) [129–132]. According to our included articles, detection of miRNA from respiratory system only account for 20%. More original studies using respiratory samples are needed for further study of their diagnosis value.

Still, we found 3 miRNAs including miR-21, miR-486, and miR-31 whose statistical difference between sample sources was significant. Interestingly, miR-31 had higher Sen in haematic samples while its specificity was higher in respiratory samples. The possible mechanism is still waited to be determined. According to the results of other statistically positive miRNAs (miR-210, miR-126, miR-182, and miR-200b), haematic samples owned higher Spe than respiratory ones. Here we have to admit that blood is a good sample source in the detection of miRNAs not only for it is highly accessible regardless of hospital-level but also because of the anti-RNase characteristic of miRNAs themselves [133], which might cause the current condition that researchers prefer to choose blood as sample source in the detection of miRNAs. Besides, whether the examination for sampling was invasive or not should be taken into consideration when it came to clinical practice.

Last but not less, the combination of miRNAs with other biomarkers as novel panels might serve as powerful tool for LC diagnosis. Currently, miRNA panels are widely used in the diagnosis of various cancers, such as pancreatic cancer [134,135], gastric cancer [136], bladder cancer [137] and glioma [138]. For example, the Dartmouth-Hitchcock Medical Centre demonstrated a qRT-PCR assay containing 5 miRNAs to diagnose pancreatic cancer [135] while Qian even conducted a meta-analysis in Glioma and found that panels of multiple miRNAs enhanced the diagnostic sensitivity [138]. We also conduct analysis on the diagnostic potential of miRNA panels in this article. However, our panels are not satisfying enough since we could not verify its power and the involved miRNAs were numerous. There are also other biomarker panels of LC, which contains peptides or proteins [139] (such as ProGRP, NSE, CEA, CYFRA21-1, HE4), genes [140] (such as TP53, STK11, RTK) and lncRNA [141].

There are still some limitations to this analysis. Due to insufficiency of available data, we did not conducted analysis on ethnic or NSCLC subtypes’ subgroups. Besides, the clinical relevance of miRNAs involved in the miRNA panel and models we constructed in this article was still lacking and the further optimization of this panel was waited to conduct. We also had to admit that the score of our patients’ quality according to QUADAS-2 was dissatisfactory because all of our included articles were case-control study. Even so we still brought those articles into analysis not only because their total score of quality were favoured, but also for the lacking of high-quality original articles.

## 5. Conclusion

In summary, we identified several single miRNAs as potential diagnostic biomarkers of NSCLC as well as sub-analysis of early-stage NSCLC through pooled calculation of their sensitivity, specificity and AUC value. In addition, we found 6 miRNAs with statistical difference between blood-based and respiratory-based sample sources. Innovatively, we discovered 1 miRNA panel (miR-210, miR-31 and miR-21) with great diagnostic power among available panels and furtherly defined 2 models of miRNA groups with satisfying value on NSCLC diagnosing. All our results suggested that single miRNA and miRNA panel could serve as a diagnosis biomarker for NSCLC, which also need to be further verified in independence clinical samples.

## Supplementary Material

Supplemental Material

## Data Availability

The authors confirm that the data supporting the findings of this study are available within the article and its supplementary materials.
