# Supplementary material for: High diagnostic value of miRNAs for NSCLC: quantitative analysis for both single and combined miRNAs in lung cancer
Source: Ann Med. 2021 Dec 16;53(1):2178–93. doi: 10.1080/07853890.2021.2000634 (PMC8740622; doi:10.1080/07853890.2021.2000634)
Supplement: Supplemental Material [file IANN_A_2000634_SM6817.zip › Supplemental files/20210918_Appendix 1_Tables.pdf]

Supplementary material 1: Appendix Tables

Appendix Table 1. The basic diagnostic data of 20 single-miRNAs in NSCLC extracted from all included papers.

Appendix Table 2. The Threshold effect analysis of analyzed 20 single-miRNAs in NSCLC.

Appendix Table 3. The results of correction publication bias for 6 kinds of miRNAs by trim and fill method.

Appendix Table 4. The basic diagnostic data extracted of 4 miRNAs included in unclassified LC.

Appendix Table 5. The Threshold effect analysis of 4 single-miRNA in unclassified LC.

Appendix Table 6. The overall diagnostic value of 4 single miRNAs in unclassified LC.

Appendix Table 7. The basic NSCLC diagnostic data extracted of different miRNA panels.

Appendix Table 1. The basic diagnostic data of 20 single-miRNAs in NSCLC extracted from all included papers.

No.	Study ID	Case/Control	Stage	Case-type	Control-type	Sample sources	TP	FP	FN	TN	Sensitivity	Specificity	AUC
miR-21													
1	2020.Wu, Q.	48/48	I-II	NSCLC	HC	Serum exosomal	40	16	8	32	83.33%	66.25%	0.75
2	2020.Wang, W.	26/14	I	NSCLC	Cancer-free	Blood	4	0	22	14	15.40%	100.00%	0.58
4	2019.Wang, S.	50/24	N/S	NSCLC	HC	Plasma	30	8	20	16	60.00%	66.70%	0.65
5	2019.Abdollahi, A.	43/43	I-IV	NSCLC	Cancer-free	Blood	39	14	4	29	90.00%	67.00%	0.8500
6	2018.Yang, Y.	104/50	I-IV	NSCLC	Cancer-free	Serum	71	11	33	39	68.27%	78.00%	0.77
7	2018.Xi, K. X.	42/15	I-II	NSCLC	Cancer-free	Plasma	33	6	9	9	78.60%	60.00%	0.675
8	2018.Sun, Y.	28/28	I-III	AD	HC	Plasma	23	1	5	27	82.10%	96.40%	0.88
11	2017.Zhang, H.	129/83	I-II	NSCLC	HC	Plasma	101	12	28	71	78.00%	86.00%	0.84
12	2017.Leng, Q.	92/88	I-IV	NSCLC	Cancer-free	Plasma	54	32	38	56	58.73%	63.33%	0.58
14	2016.Su, Y.(1)	117/174	I	NSCLC	Cancer-free	Sputum	93	50	24	124	79.47%	70.98%	0.80
15	2016.Chen, J. L.	30/30	I-II	NSCLC	HC	Sputum	26	8	4	22	86.70%	73.30%	0.858
16	2015.Zhao, W.	80/60	N/S	NSCLC	HC	Serum	59	17	21	43	73.80%	71.70%	0.81
17	2015.Yang, J. S.	152/300	I-IV	NSCLC	HC	Serum	105	87	47	213	69.00%	71.00%	0.81
18	2015.Xing, L.	60/62	I-II	NSCLC	Cancer-free	Sputum	47	18	13	44	78.16%	71.08%	0.82
19	2015.Su, J.	56/73	N/S	NSCLC	Cancer-free	Sputum	42	14	14	59	75.00%	80.28%	0.81
20	2014.Geng, Q.(b)	126/102	I-II	NSCLC	Cancer-free	Plasma	84	33	42	69	67.00%	68.00%	0.77
21	2014.Geng, Q.(a)	89/42	I-II	NSCLC	Cancer-free	Plasma	69	10	20	32	78.00%	75.00%	0.84
22	2013.Tang, D.(b)	34/32	I-III	NSCLC	HC	Plasma	18	9	16	23	52.90%	71.90%	0.71
23	2013.Tang, D.(a)	62/60	I-III	NSCLC	HC	Plasma	30	13	32	47	48.40%	78.30%	0.72
24	2013.Mozzoni, P.	54/46	I-III	NSCLC	Cancer-free	Plasma	27	4	27	42	50.00%	92.30%	0.74
28	2011.Wei, J.(1)	63/30	I-IV	NSCLC	HC	Plasma	48	9	15	21	76.20%	70.00%	0.78
27	2011.Wei, J.(2)	77/36	I-IV	NSCLC	HC	Plasma	47	6	30	30	61.04%	83.33%	0.73
30	2011.Li, Y.	20/10	I-IV	NSCLC	Cancer-free	PB	16	0	4	10	78.80%	100.00%	0.91
31	2010.Yu, L.	36/36	I	AD	HC	Sputum	26	7	10	29	72.60%	79.20%	0.85
32	2010.Xie, Y.	23/17	I-IV	NSCLC	Cancer-free	Sputum	16	0	7	17	69.66%	100.00%	0.90

miR-210													
1	2020.Wang, W.	26/14	I	NSCLC	Cancer-free	Blood	9	1	17	13	34.60%	92.90%	0.64
3	2019.Switlik, W. Z.	14/29	N/S	AD	HC	Serum	12	6	2	23	86.00%	79.00%	0.84
6	2018.Leng, Q.	56/28	I-IV	NSCLC	Cancer-free	Plasma	36	5	20	23	64.29%	82.14%	0.75
7	2018.Bagheri, A.	30/30	I-IV	NSCLC	Cancer-free	Sputum	30	18	0	12	100.00%	40.00%	0.69
8	2017.Leng, Q.	92/88	I-IV	NSCLC	Cancer-free	Plasma	61	32	31	56	66.43%	63.33%	0.73
9	2016.Zhu, W.	112/40	I-III	NSCLC	HC	Serum	38	0	74	40	33.90%	100.00%	0.62
10	2016.Wang, X.	59/59	I-III	NSCLC	Cancer-free	Plasma	44	15	15	44	74.60%	74.60%	0.75
11	2016.Su, Y.[1]	117/174	I	NSCLC	Cancer-free	Sputum	90	33	27	141	76.58%	81.16%	0.84
12	2015.Xing, L.	60/62	I-II	NSCLC	Cancer-free	Sputum	45	9	15	53	75.27%	85.88%	0.85
13	2015.Su, J.	56/73	N/S	NSCLC	Cancer-free	Sputum	46	16	10	57	82.76%	78.56%	0.85
16	2010.Xing, L.	48/48	I	SCC	HC	Sputum	28	10	20	38	58.00%	79.00%	0.75
miR-145													
1	2020.Wang, W.	26/14	I	NSCLC	Cancer-free	Blood	3	0	23	14	11.50%	100.00%	0.56
2	2019.Sui, A.	76/60	I-IV	NSCLC	HC	Serum	64	17	12	43	84.21%	71.67%	0.88
5	2018.Bagheri, A.	30/30	I-IV	NSCLC	Cancer-free	Sputum	30	10	0	20	100.00%	66.70%	0.88
6	2018.Aiso, T.	56/26	I-IV	NSCLC	Cancer-free	Serum	40	3	16	23	71.40%	88.50%	0.83
7	2017.Zhang, H.	129/83	I-II	NSCLC	HC	Plasma	104	9	25	74	81.00%	89.00%	0.89
8	2017.Leng, Q.	92/88	I-IV	NSCLC	Cancer-free	Plasma	66	25	26	63	71.43%	71.33%	0.72
9	2015.Wang, R. J.	70/70	N/S	NSCLC	HC	Serum	65	27	5	43	92.75%	61.43%	0.84
11	2014.Geng, Q.[a]	126/102	I-II	NSCLC	Cancer-free	Plasma	88	33	38	69	70.00%	68.00%	0.77
10	2014.Geng, Q.[b]	25/25	I-II	NSCLC	HC	Plasma	18	6	7	19	73.00%	75.00%	0.82
13	2013.Tang, D.[a]	62/60	I-III	NSCLC	HC	Plasma	38	27	24	33	61.30%	55.00%	0.63
12	2013.Tang, D.[b]	34/32	I-III	NSCLC	HC	Plasma	19	14	15	18	55.90%	56.30%	0.66
14	2010.Yu, L.	36/36	I	AD	HC	Sputum	21	6	15	30	59.50%	82.90%	0.81
miR-155													
2	2020.Liu, X.	128/128	I-IV	NSCLC	HC	Serum	106	43	22	85	82.50%	66.70%	0.798
4	2018.Xi, K. X.	42/15	I-II	NSCLC	Cancer-free	Plasma	26	4	16	11	61.90%	73.30%	0.68

7	2015.Xing, L.	60/62	I-II	NSCLC	Cancer-free	Sputum	38	23	22	39	62.67%	62.96%	0.70
9	2014.Geng, Q.[(a)]	126/102	I-II	NSCLC	Cancer-free	Plasma	108	16	18	86	86.00%	84.00%	0.92
8	2014.Geng, Q.[(b)]	25/25	I-II	NSCLC	HC	Plasma	22	3	3	22	87.00%	87.00%	0.94
11	2013.Tang, D.[(a)]	62/60	I-III	NSCLC	HC	Plasma	37	15	25	45	59.70%	75.00%	0.73
10	2013.Tang, D.[(b)]	34/32	I-III	NSCLC	HC	Plasma	23	11	11	21	67.60%	65.60%	0.74
miR-486													
1	2020.Wu, Q.	48/48	I-II	NSCLC	HC	Serum exosomal	37	16	11	32	77.08%	66.25%	0.78
2	2020.Liu, C.	64/15	I-IV	NSCLC	HC	Serum exosomal	47	1	17	14	73.40%	93.30%	0.91
3	2018.Poroyko, V.	9/10	N/S	SCLC	HC	Serum exosomal	9	1	0	9	100.00%	90.00%	0.98
5	2017.Leng, Q.	92/88	I-IV	NSCLC	Cancer-free	Plasma	65	15	27	73	70.97%	83.33%	0.80
6	2016.Wang, X.	59/59	I-III	NSCLC	Cancer-free	Plasma	49	13	10	46	83.10%	78.00%	0.85
8	2015.Xing, L.	60/62	I-II	NSCLC	Cancer-free	Sputum	44	21	16	41	74.03%	66.67%	0.75
9	2015.Li, W.	11/11	N/S	NSCLC	HC	Plasma	10	2	1	9	90.90%	81.80%	0.93
10	2013.Mozzoni, P.	54/46	I-III	NSCLC	Cancer-free	Plasma	38	5	16	41	70.00%	89.50%	0.86
11	2011.Shen, J.[(1)]	58/29	I-IV	NSCLC	HC	Plasma	49	10	9	19	84.48%	65.52%	0.68
12	2010.Yu, L.	36/36	I	AD	HC	Sputum	24	7	12	29	66.90%	79.40%	0.83
13	2020.Wang, J. W.	82/90	I-IV	AD	HC	Serum	58	37	24	53	70.70%	58.90%	0.66
miR-17													
2	2020.Yang, C.[(a)]	14/23	I-III	NSCLC	HC	Serum	12	7	2	16	82.50%	68.18%	0.8097
1	2020.Yang, C.[(b)]	9/23	IV	NSCLC	HC	Serum	9	0	0	23	100.00%	100.00%	1.0000
3	2020.Wang, W.	26/14	I	NSCLC	Cancer-free	Blood	3	0	23	14	11.50%	100.00%	0.56
7	2019.Zhang, Y.[(a)]	100/90	I-III	NSCLC	HC	Serum exosomal	70	16	30	74	70.00%	82.20%	0.75
6	2019.Zhang, Y.[(b)]	72/47	I-III	NSCLC	HC	Serum exosomal	48	11	24	36	66.70%	76.60%	0.74
8	2019.Hetta, H. F.	40/20	II-IV	NSCLC	HC	Plasma	31	2	9	18	77.78%	87.50%	0.83
9	2018.Xi, K. X.	42/15	I-II	NSCLC	Cancer-free	Plasma	28	3	14	12	66.70%	80.00%	0.711
10	2018.Fan, L. H.	128/79	I	NSCLC	HC	Serum	97	27	31	52	75.90%	65.90%	N/S
11	2015.Fan, L.	94/58	I-III	NSCLC	HC	Serum	77	19	17	39	81.60%	67.90%	N/S
miR-126													

1	2020.Wang, W.	26/14	I	NSCLC	Cancer-free	Blood	4	1	22	13	15.40%	92.90%	0.54
4	2018.Bagheri, A.	30/30	I-IV	NSCLC	Cancer-free	Sputum	29	7	1	23	96.70%	76.70%	0.81
5	2017.Shang, A. Q.	127/112	I-IV	NSCLC	HC	Serum	105	4	22	108	82.68%	96.40%	0.87
6	2017.Leng, Q.	92/88	I-IV	NSCLC	Cancer-free	Plasma	66	23	26	65	71.43%	73.33%	0.78
7	2016.Zhu, W.	112/40	I-III	NSCLC	HC	Serum	68	3	44	37	60.70%	92.50%	0.79
8	2015.Xing, L.	60/62	I-II	NSCLC	Cancer-free	Sputum	47	15	13	47	77.63%	75.00%	0.78
11	2010.Yu, L.	36/36	I	AD	HC	Sputum	24	9	12	27	67.20%	73.80%	0.82
12	2010.Xing, L.	48/48	I	SCC	HC	Sputum	29	10	19	38	60.00%	79.00%	0.70
miR-223													
2	2018.Yang, Y.	104/50	I-IV	NSCLC	Cancer-free	Serum	80	10	24	40	76.92%	80.00%	0.74
3	2018.Leng, Q.	56/28	I-IV	NSCLC	Cancer-free	Plasma	41	11	15	17	73.21%	60.71%	0.67
4	2018.Aiso, T.	56/26	I-IV	NSCLC	Cancer-free	Serum	46	12	10	14	82.10%	52.00%	0.69
5	2017.Zhang, H.	129/83	I-II	NSCLC	HC	Plasma	90	13	39	70	70.00%	84.00%	0.81
6	2017.Leng, Q.	92/88	I-IV	NSCLC	Cancer-free	Plasma	65	15	27	73	70.97%	83.33%	0.80
7	2017.Bagheri, A.	17/17	I-IV	NSCLC	Cancer-free	Sputum	14	1	3	16	82.00%	95.00%	0.90
8	2014.Geng, Q.[a]	126/102	I-II	NSCLC	Cancer-free	Plasma	110	14	16	88	87.00%	86.00%	0.94
9	2014.Geng, Q.[b]	25/25	I-II	NSCLC	HC	Plasma	22	3	3	22	87.00%	86.00%	0.96
miR-31													
1	2019.Wang, S.	50/24	N/S	NSCLC	HC	Plasma	30	6	20	18	60.00%	75.00%	0.73
2	2019.Szczyrek, M.	160/45	I-IV	NSCLC	HC	Plasma	150	9	10	36	94.00%	81.00%	0.95
3	2016.Su, Y.[1]	117/174	I	NSCLC	Cancer-free	Sputum	72	32	45	142	61.66%	81.86%	0.76
5	2015.Xing, L.	60/62	I-II	NSCLC	Cancer-free	Sputum	36	11	24	51	60.23%	82.67%	0.79
6	2015.Su, J.	56/73	N/S	NSCLC	Cancer-free	Sputum	36	12	20	61	64.26%	83.93%	0.77
miR-20													
1	2020.Yang, C.[a]	14/23	I-III	NSCLC	HC	Serum	13	6	1	17	90.20%	72.73%	0.8975
2	2020.Yang, C.[b]	9/23	IV	NSCLC	HC	Serum	9	0	0	23	100.00%	100.00%	1.0000
3	2020.Wu, Q.	48/48	I-II	NSCLC	HC	Serum exosomal	33	5	15	43	68.75%	90.00%	0.81
4	2020.Wang, W.	26/14	I	NSCLC	Cancer-free	Plasma	3	0	23	14	11.50%	100.00%	0.56

5	2019.Xi, K.	28/12	I-II	NSCLC	Cancer-free	Serum	19	1	9	11	67.90%	91.70%	0.73
6	2018.Xi, K. X.	42/15	I-II	NSCLC	Cancer-free	Plasma	23	2	19	13	54.80%	86.70%	0.71
7	2017.Leng, Q.	92/88	I-IV	NSCLC	Cancer-free	Plasma	66	23	26	65	71.43%	73.33%	0.78
8	2015.Wang, R. J.	70/70	N/S	NSCLC	HC	Plasma	59	29	11	41	84.06%	58.57%	0.78
miR-182													
1	2020.Wang, W.	26/14	I	NSCLC	Cancer-free	Blood	5	0	21	14	19.20%	100.00%	0.60
2	2019.Zou, J. G.	50/30	N/S	NSCLC	HC	Serum	27	13	23	17	53.00%	57.00%	0.60
3	2019.Szczyrek, M.	160/45	I-IV	NSCLC	HC	Plasma	112	9	48	36	70.00%	79.00%	0.77
5	2016.Zhu, W.	112/40	I-III	NSCLC	HC	Serum	71	8	41	32	63.40%	80.00%	0.73
6	2015.Xing, L.	60/62	I-II	NSCLC	Cancer-free	Sputum	39	25	21	37	64.94%	59.76%	0.68
miR-146													
1	2020.Wang, J. W.	82/90	I-IV	AD	HC	Serum	55	21	27	69	67.10%	76.70%	0.75
2	2020.Wu, Q.	48/48	I-II	NSCLC	HC	Serum exosomal	33	5	15	43	68.75%	90.00%	0.81
3	2020.Wang, W.	26/14	I	NSCLC	Cancer-free	Blood	3	0	23	14	11.50%	100.00%	0.56
4	2019.Xi, K.	28/12	I-II	NSCLC	Cancer-free	Plasma	19	1	9	11	67.90%	91.70%	0.73
5	2018.Xi, K. X.	42/15	I-II	NSCLC	Cancer-free	Plasma	23	2	19	13	54.80%	86.70%	0.71
6	2017.Leng, Q.	92/88	I-IV	NSCLC	Cancer-free	Plasma	54	35	38	53	58.49%	60.00%	0.64
7	2015.Wang, R. J.	70/70	N/S	NSCLC	HC	Serum	59	29	11	41	84.06%	58.57%	0.78
miR-205													
1	2020.Wang, W.	26/14	I	NSCLC	Cancer-free	Blood	5	0	21	14	19.20%	100.00%	0.60
2	2019.Zou, J. G.	50/30	N/S	NSCLC	HC	Serum	28	11	22	19	55.80%	63.60%	0.70
5	2018.Leng, Q.	56/28	I-IV	NSCLC	Cancer-free	Plasma	39	9	17	19	69.64%	67.86%	0.75
6	2017.Leng, Q.	92/88	I-IV	NSCLC	Cancer-free	Plasma	61	32	31	56	66.43%	63.33%	0.73
8	2015.Xing, L.	60/62	I-II	NSCLC	Cancer-free	Sputum	36	29	24	33	59.74%	53.93%	0.64
10	2010.Xing, L.	48/48	I	SCC	HC	Sputum	31	5	17	43	65.00%	90.00%	0.79
miR-19													
1	2020.Yang, C.[a]	14/23	I-III	NSCLC	HC	Serum	11	10	3	13	78.85%	55.56%	0.8451
2	2020.Yang, C.[b]	9/23	IV	NSCLC	HC	Serum	7	13	2	10	75.00%	44.44%	0.5104

4	2018.Fan, L. H.	128/79	I	NSCLC	HC	Serum	93	25	35	54	72.30%	67.90%	N/S
5	2017.Leng, Q.	92/88	I-IV	NSCLC	Cancer-free	Plasma	63	32	29	56	68.25%	63.33%	0.66
7	2015.Fan, L.	94/58	I-III	NSCLC	HC	Serum	78	19	16	39	83.30%	66.70%	N/S
miR-221													
1	2020.Wang, W.	26/14	I	NSCLC	Cancer-free	Blood	3	1	23	13	11.50%	92.90%	0.52
2	2020.Ghany, S. M. A.	70/34	N/S	NSCLC	HC	Plasma	28	8	42	26	40.00%	75.00%	N/S
4	2018.Xi, K. X.[(b)]	42/15	I-II	NSCLC	Cancer-free	Plasma	23	3	19	12	54.80%	80.00%	0.687
5	2018.Poroyko, V.	11/10	N/S	NSCLC	HC	Serum exosomal	11	2	0	8	100.00%	80.00%	0.95
6	2017.Leng, Q.	92/88	I-IV	NSCLC	Cancer-free	Plasma	53	42	39	46	57.14%	51.72%	0.58
miR-200													
1	2020.Wang, W.	26/14	I	NSCLC	Cancer-free	Blood	9	1	17	13	34.60%	92.90%	0.64
2	2019.Zou, J. G.	50/30	N/S	NSCLC	HC	Serum	28	11	22	19	55.10%	62.10%	0.67
3	2019.Xi, K.	28/12	I-II	NSCLC	Cancer-free	Plasma	19	2	9	10	67.90%	83.30%	0.70
5	2015.Xing, L.	60/62	I-II	NSCLC	Cancer-free	Sputum	39	24	21	38	65.22%	61.19%	0.68
7	2010.Yu, L.	36/36	I	AD	HC	Sputum	23	8	13	28	62.90%	78.50%	0.82
Let-7													
1	2019.Wang, S.	50/24	N/S	NSCLC	HC	Plasma	27	10	23	14	54.00%	58.30%	0.67
2	2018.Leng, Q.	56/28	I-IV	NSCLC	Cancer-free	Plasma	38	9	18	19	68.23%	67.53%	0.71
3	2018.Leng, Q.	56/28	I-IV	NSCLC	Cancer-free	Plasma	36	11	20	17	63.92%	60.25%	0.68
4	2018.Leng, Q.	56/28	I-IV	NSCLC	Cancer-free	Plasma	33	11	23	17	59.66%	61.26%	0.67
5	2015.Dou, H.	120/360	I-IV	NSCLC	HC	Plasma	86	79	34	281	72.00%	78.00%	0.71
6	2014.Zhu, W. Y.	36/44	I	NSCLC	HC	Serum	18	7	18	37	50.00%	83.30%	0.64
7	2011.Jeong, H. C.	35/30	I-IV	NSCLC	HC	Blood	32	3	3	27	90.30%	90.30%	0.95
miR-125													
1	2018.Bagheri, A.	30/30	I-IV	NSCLC	Cancer-free	Sputum	24	12	6	18	80.00%	60.00%	0.66
2	2015.Wang, R. J.	70/70	N/S	NSCLC	HC	Serum	51	31	19	39	73.53%	55.71%	0.71
4	2014.Zhu, W. Y.	36/44	I	NSCLC	HC	Serum	19	11	17	33	53.50%	75.00%	0.65
5	2020.Zhang, Z. J.	330/312	I-IV	NSCLC	HC	Serum exosomal	205	94	125	218	62.40%	70.00%	0.70

miR-7												
1	2020.Wang, W.	26/14	I	NSCLC	Cancer-free	Blood	5	0	21	14	19.20%	100.00%	0.60
2	2019.Xi, K.	28/12	I-II	NSCLC	Cancer-free	Plasma	21	2	7	10	75.00%	83.30%	0.71
4	2018.Xi, K. X.[(b)]	42/15	I-II	NSCLC	Cancer-free	Plasma	33	4	9	11	78.60%	73.30%	0.735
5	2018.Bagheri, A.	30/30	I-IV	NSCLC	Cancer-free	Sputum	22	5	8	25	73.30%	83.30%	0.77
miR-10													
1	2019.Sheervalilou, R.[(a)]	47/41	N/S	NSCLC	Cancer-free	Plasma	37	9	10	32	78.00%	78.00%	N/S
2	2019.Sheervalilou, R.[(b)]	47/41	I-IV	NSCLC	Cancer-free	Plasma	37	9	10	32	78.00%	78.00%	0.90
3	2018.Yang, Y. L.	194/199	III-IV	NSCLC	HC	PBMCs	165	1	29	198	85.10%	99.50%	0.97
4	2018.Bao, M.	80/75	I-IV	NSCLC	HC	Serum	53	20	27	55	65.98%	72.71%	0.71
5	2017.Sheervalilou, R.[(a)]	30/30	I-III	NSCLC	Cancer-free	Sputum	27	6	3	24	90.00%	80.00%	0.927
6	2017.Sheervalilou, R.[(b)]	30/30	I-III	NSCLC	Cancer-free	BALF	26	3	4	27	86.00%	90.00%	0.931

(1), (2): Different articles by the same author in the same year; (a), (b): Different diagnosis data in the same article; N/S: not specified.
AD: Adenocarcinoma; SCC: Squamous cell carcinoma; NSCLC: Non-Small Cell Lung Carcinoma; LC: lung cancer; HC: health control.
EBC: exhaled breath condensate; PLF: Pleural lavage fluid; PB: Peripheral blood; PBMCs: Peripheral blood mononuclear cells; BALF: bronchoalveolar lavage; LT: Lung tissue.
TP: true positive; FP: false positive; FN: false negative; TN: true negative; AUC: Area Under Curve; Sen: sensitivity; Spe: Specificity.

Appendix Table 2. The Threshold effect analysis of analyzed 20 single-miRNAs in NSCLC.

miRNA-type	Spearman Coefficient	*P*-value
miR-21	-0.194	0.3527
miR-210	-0.615	0.044
miR-145	-0.1329	0.6806
miR-486	-0.2485	0.4888
miR-126	-0.1905	0.6514
miR-155	0.4286	0.3374
miR-205	-0.2571	0.6228
miR-17	-0.1429	0.7139
miR-182	-0.3714	0.4685
miR-223	0.3012	0.4685
miR-31	0.3002	0.6238
miR-200	-0.3	0.6238
miR-20	-0.3234	0.4346
miR-146	-0.5429	0.2657
miR-19	-0.1	0.8729
miR-125	-0.8	0.2
miR-7	-0.9487	0.0513
miR-221	-0.4104	0.4925
Let-7	0.4286	0.3374
miR-10	0.7647	0.0765

Spearman coefficient was used to evaluate the threshold effect of 20 miRNAs. When the Spearman coefficient was greater than 0.6 and the *P*-value was less than 0.05, the threshold effect was considered to exist and could not be further analyzed.

Appendix Table 3. The results of correction publication bias for 6 kinds of miRNAs by trim and fill method.

miRNA	No. of research	pooled OR (95%*CI*) before Trimming	pooled OR (95%*CI*) after Trimming
miR-17	9	3.051 (1.985-4.117)	3.051 (1.985-4.117)
miR-182	8	1.911 (1.011-2.810)	1.378 (0.409-2.348)
miR-31	7	2.119 (1.882-2.356)	2.119 (1.882-2.356)
miR-146	7	1.976 (1.076-2.876)	1.835 (1.000-2.670)
miR-19	6	2.097 (1.438-2.755)	2.097 (1.438-2.755)
miR-125	5	1.864 (1.444-2.283)	1.820 (0.880-2.760)

OR: odd ratio; *CI*: confidence interval.

Appendix Table 4. The basic diagnostic data extracted of 4 miRNAs included in unclassified LC.

No.	Study ID	Case/Control	Stage	Case-type	Control-type	Sample sources	TP	FP	FN	TN	Sen	Spe	AUC
miR-375													
1	2018.Bagheri, A.	30/30	I-IV	NSCLC	Cancer-free	Sputum	13	4	17	26	43.30%	86.70%	0.57
2	2015.Xing, L.	60/62	I-II	NSCLC	Cancer-free	Sputum	40	23	20	39	66.23%	62.22%	0.67
3	2011.Shen, J[1].	66/68	I-IV	LC	Cancer-free	Sputum	35	27	31	41	53.60%	61.00%	0.64
4	2010.Yu, L.	36/36	I	AD	HC	Sputum	23	7	13	29	63.00%	80.60%	0.82
miR-150													
1	2019.Roman-Canal, B.	14/20	N/S	LC	Cancer-free	PLF	12	1	2	19	85.70%	95.00%	0.939
2	2015.Li, W.	11/11	N/S	NSCLC	HC	Plasma	9	2	2	9	81.80%	81.80%	0.752
3	2013.Zeng, X. L.	34/26	I-IV	NSCLC	HC	PBMCs	30	8	4	18	87.50%	69.20%	0.834
4	2020.Wang, J. W.	82/90	I-IV	AD	HC	Serum	62	51	20	39	75.60%	43.30%	0.602
miR-92													
1	2020.Yang, C.[(a)]	14/23	I-III	NSCLC	HC	Serum	12	9	2	14	83.02%	59.09%	0.8097
2	2020.Yang, C.[(b)]	9/23	IV	NSCLC	HC	Serum	7	8	2	15	76.47%	63.64%	0.8342
3	2017.Yu, Y.	50/30	I-IV	SCLC	HC	Plasma	28	0	22	30	56.00%	100.00%	0.76
4	2017.Leng, Q.	92/88	I-IV	NSCLC	Cancer-free	Plasma	66	12	26	76	71.43%	86.87%	0.79
5	2015.Fan, L.	94/58	I-III	NSCLC	HC	Serum	82	20	12	38	86.80%	65.50%	N/S
miR-25													
1	2020.Liu, C.[(a)]	64/15	I-IV	LC	HC	Serum	56	3	8	12	87.50%	80.00%	0.86
2	2020.Liu, C.[(b)]	64/15	I-IV	LC	HC	Serum	46	7	18	8	71.90%	50.00%	0.54
3	2020.Liu, C.[(c)]	32/15	III-IV	LC	HC	Serum	29	3	3	12	90.60%	80.00%	0.87
4	2020.Liu, C.[(d)]	32/15	I-II	LC	HC	Serum	27	3	5	12	84.40%	80.00%	0.86
5	2020.Liu, C.[(e)]	32/15	I-II	LC	HC	Serum	25	7	7	8	78.10%	50.00%	0.56
6	2020.Liu, C.[(f)]	32/15	III-IV	LC	HC	Serum	26	10	6	5	81.20%	35.70%	0.51
7	2018.Poroyko, V.	11/10	N/S	NSCLC	HC	Lung tissue	9	1	2	9	83.33%	90.00%	0.91
8	2016.Zaporozhchenko, I. A.[(a)]	75/50	II-IV	LC	HC	Plasma	42	8	33	42	56.20%	84.00%	0.66

9	2016.Zaporozhchenko, I. A.(b)	53/50	II-IV	SCC	HC	Plasma	36	8	17	42	68.00%	84.00%	0.81
10	2016.Zaporozhchenko, I. A.(c)	18/50	II-IV	AD	HC	Plasma	10	8	8	42	53.90%	84.00%	0.81
11	2015.Wang, P.	94/111	I-II	LC	HC	Serum	78	28	16	83	83.30%	75.00%	0.82

(a), (b): Different diagnosis data in the same article; N/S: not specified.

AD: Adenocarcinoma; SCC: Squamous cell carcinoma; NSCLC: Non-Small Cell Lung Carcinoma; LC: lung cancer; HC: health control.

EBC: exhaled breath condensate; PLF: Pleural lavage fluid; PB: Peripheral blood; PBMCs: Peripheral blood mononuclear cells; BALF: bronchoalveolar lavage; LT: Lung tissue; TP: true positive; FP: false positive; FN: false negative; TN: true negative; AUC: Area Under Curve; Sen: sensitivity; Spe: Specificity.

Appendix Table 5. The Threshold effect analysis of 4 single-miRNA in unclassified LC.

**miRNA-type**	**Spearman Coefficient**	***P*-value**
miR-375	-0.4	0.6
miR-92	-0.7	0.1881
miR-25	-0.1624	0.6332
miR-150	0.4	0.6

Spearman coefficient was used to evaluate the threshold effect of 4 miRNAs. When the Spearman coefficient was greater than 0.6 and the *P*-value was less than 0.05, the threshold effect was considered to exist and could not be further analyzed.

Appendix Table 6. The overall diagnostic value of 4 single miRNAs in unclassified LC.

miRNA-type	No. of research (Case/Control)	Sen [95% *CI*]	Spe [95% *CI*]	PLR [95% *CI*]	NLR [95% *CI*]	AUC [95% *CI*]	DOR [95% *CI*]
miR-375	4 (192/196)	0.57 [0.48 - 0.66]	0.72 [0.59 - 0.83]	2.1 [1.4 - 3.1]	0.60 [0.48 - 0.74]	0.65 [0.61 - 0.69]	3 [2 - 6]
miR-92	4 (259/222)	0.78 [0.66 - 0.87]	0.80 [0.60 - 0.92]	4.0 [2.0 - 7.9]	0.27 [0.19 - 0.39]	0.85 [0.82 - 0.88]	15 [8 - 27]
miR-25	4 (507/361)	0.77 [0.70 - 0.83]	0.75 [0.66 - 0.82]	3.0 [2.2 - 4.2]	0.31 [0.23 - 0.41]	0.83 [0.79 - 0.86]	10 [6 - 16]
miR-150	4 (141/147)	0.84 [0.72 - 0.91]	0.75 [0.48 - 0.90]	3.3 [1.3 - 8.1]	0.22 [0.10 - 0.45]	0.87 [0.83 - 0.89]	15 [3 - 71]

Sen: sensitivity; Spe: specificity; PLR: positive likelihood ratio; NLR: negative likelihood ratio; AUC: Area Under Curve; DOR: diagnostic odds ratio;

Appendix Table 7. The basic NSCLC diagnostic data extracted of different miRNA panels.

Study ID	Case/ Control	Stage	Case-type	Control-type	miRNA	Sample	TP	FP	FN	TN	Sen	Spe	AUC
**2020.Liao, J.[(a)]**	76/72	I-IV	NSCLC	Cancer-free	miR-31-5p, -210-3, -21-5p	Sputum, Plasma	65	6	11	66	85.50%	91.70%	0.91
**2020.Liao, J.[(b)]**	76/72	I-IV	NSCLC	Cancer-free	miR-31-5p, -210-3p, -21-5p	Plasma	57	12	19	60	75.00%	83.33%	0.85
**2020.Liao, J.[(d)]**	56/55	I-IV	NSCLC	Cancer-free	miR-31-5p, -210-3p, -21-5p	Plasma	51	9	5	46	90.90%	83.60%	NS
**2016.Su, Y[2].**	57/62	I	NSCLC	Cancer-free	miR-21, -31, -210	Sputum	47	9	10	53	82.61%	85.45%	0.90
**2016.Su, Y[1].**	117/174	I	NSCLC	Cancer-free	miR-21, -31, -210	Sputum	95	25	22	149	81.48%	85.91%	0.89
**2015.Xing, L.(a)**	76/79	I-II	NSCLC	Cancer-free	miR-21, -31, -210	Sputum	61	11	15	68	80.52%	86.08%	NS
**2015.Xing, L.(b)**	67/69	I-II	NSCLC	Cancer-free	miR-21, -31, -210	Sputum	55	8	12	61	82.09%	88.41%	NS
**2015.Xing, L.(c)**	60/62	I-II	NSCLC	Cancer-free	miR-21, -31, -210	Sputum	50	8	10	54	82.93%	87.84%	0.92
**2015.Su, J.**	56/73	NA	NSCLC	Cancer-free	miR-31, -21, -210	Sputum	47	9	9	64	83.93%	87.67%	0.91
**2020.Wu, Q.**	48/48	I-II	NSCLC	HC	miR-146a-5p, -486-5p	Serum	40	5	8	43	83.33%	90.00%	0.90
**2020.Wang, W.**	26/14	I	NSCLC	Cancer-free	miR-17, -146a, -200b, -miR-182, -155, -221, -205, -126, -7, -21, -145, -210	Blood	13	1	13	13	50.00%	92.90%	0.71
**2020.Liu, C.[(a)]**	64/15	I-IV	LC	HC	miR-21, -25, -155, -210, -486	Serum	62	1	2	14	96.90%	93.30%	0.97
**2020.Liu, C.[(b)]**	64/15	I-IV	LC	HC	miR-21, -25, -155, -210, -486	Serum	30	0	34	15	46.90%	100.00%	0.79
**2020.Liu, C.[(c)]**	32/15	I-II	LC	HC	miR-21, -25, -155, -210, -486	Serum	32	0	0	15	100.00%	100.00%	1.00
**2020.Liu, C.[(d)]**	32/15	III-IV	LC	HC	miR-21, -25, -155, -210, -486	Serum	31	1	1	14	96.90%	93.30%	0.96
**2020.Liu, C.[(e)]**	32/15	III-IV	LC	HC	miR-21, -25, -155, -210, -486	Serum	30	4	2	11	93.80%	71.40%	0.87
**2020.Liu, C.[(f)]**	32/15	I-II	LC	HC	miR-21, -25, -155, -210, -486	Serum	25	2	7	13	78.10%	85.70%	0.82
**2020.Liao, J.[(c)]**	76/72	I-IV	NSCLC	Cancer-free	miR-31-5p, -210-3p	Sputum	50	9	26	63	65.79%	87.50%	0.82
**2020.Liao, J.[(e)]**	56/55	I-IV	NSCLC	Cancer-free	miR-31-5p, -210-3p	Sputum	47	14	9	41	83.90%	75.00%	NS

**2019.Zou, J. G.**	50/30	NA	NSCLC	HC	miR-182, -200b, -205	Serum	29	9	21	21	58.30%	69.60%	0.78
**2019.Xi, K.[(a)]**	39/13	I-II	NSCLC	Cancer-free	miR-146a, -200b, -7	Plasma	28	4	11	9	71.80%	69.20%	0.78
**2019.Xi, K.[(b)]**	28/12	I-II	NSCLC	Cancer-free	miR-146a, -200b, -7	Plasma	26	2	2	10	92.90%	83.30%	0.93
**2019.Wang, S.[(a)]**	50/24	NA	NSCLC	HC	miR-21, -31, Let-7	Plasma	36	6	14	18	72.00%	75.00%	0.80
**2019.Wang, S.[(b)]**	50/24	NA	NSCLC	HC	miR-21, -31	Plasma	35	7	15	17	70.00%	70.80%	0.72
**2019.Wang, S.[(c)]**	50/24	NA	NSCLC	HC	miR-31, Let-7	Plasma	30	6	20	18	60.00%	75.00%	0.73
**2019.Li, J.**	232/243	I-IV	LC	Cancer-free	miR-210-3p, -145, -126-3p, -205-5p	Plasma	213	10	19	233	91.81%	95.88%	0.96
**2018.Qiu, F.**	58/42	NA	LC	HC	miR-19, -21	PB	50	2	8	40	86.60%	97.62%	0.95
**2018.Leng, Q.(a)**	56/28	I-IV	SCC	Cancer-free	miR-205-5p, -210-3p	Plasma	46	3	10	25	82.10%	89.30%	0.88
**2018.Leng, Q.(b)**	56/28	I-IV	NSCLC	Cancer-free	miR-205-5p, -210-3p	Plasma	44	3	12	25	78.60%	89.30%	0.85
**2018.Leng, Q.(c)**	56/28	I-IV	NSCLC	Cancer-free	miR-205-5p, -210-3p	Plasma	44	3	12	25	78.57%	89.29%	0.85
**2018.Leng, Q.(d)**	56/28	I-IV	AD	Cancer-free	miR-205-5p, -210-3p	Plasma	42	3	14	25	75.00%	89.30%	0.82
**2018.Bagheri, A.(a)**	30/30	I-IV	NSCLC	Cancer-free	miR-145, -126, -7	Sputum	27	3	3	27	90.00%	90.00%	0.93
**2018.Bagheri, A.(b)**	24/30	I-IV	AD	Cancer-free	miR-145, -126, -7	Sputum	22	4	2	26	93.30%	87.50%	0.93
**2018.Bagheri, A.(c)**	6/30	I-IV	SCC	Cancer-free	miR-145, -126, -7	Sputum	6	5	0	25	100.00%	83.30%	0.94
**2018.Aiso, T.**	56/26	I-IV	NSCLC	Cancer-free	miR-145, -223	Serum	48	5	8	21	85.70%	80.00%	0.89
**2017.Zhang, H.**	129/83	I-II	NSCLC	HC	miR-145, -20a, -21, -223	Plasma	106	8	23	75	81.80%	90.10%	0.90
**2017.Leng, Q.(a)**	92/88	I-IV	NSCLC	Cancer-free	miR-126, -145, -210, -205-6p	Plasma	84	3	8	85	91.50%	96.20%	0.96
**2017.Leng, Q.(b)**	92/88	I-IV	NSCLC	Cancer-free	miR-21, -210, -486-5p	Plasma	69	13	23	75	75.50%	85.30%	0.85
**2017.Leng, Q.(c)**	34/30	I-IV	NSCLC	Cancer-free	miR-126, -145, -210, -205-6p	Plasma	31	1	3	29	91.18%	96.67%	0.96
**2017.Leng, Q.(d)**	34/30	I-IV	NSCLC	Cancer-free	miR-21, -210, -486-5p	Plasma	26	5	8	25	76.47%	83.33%	0.85
**2016.Zhu, W.(e)**	112/40	I-III	NSCLC	HC	miR-182, -183, -210, -126	Serum	91	0	21	40	81.20%	100.00%	0.97
**2016.Zhu, W.(f)**	87/40	I	NSCLC	HC	miR-182, -183, -210, -126	Serum	77	3	10	37	88.50%	92.50%	0.98
**2016.Zaporozhchenko, I. A.**	75/50	II-IV	LC	HC	miR-19b, -183	Plasma	71	2	4	48	94.74%	95.24%	0.99
**2016.Wang, X.(a)**	59/59	I-III	NSCLC	Cancer-free	miR-486, -210	Plasma	50	11	9	48	84.70%	81.10%	0.92

**2016.Wang, X.(b)**	59/59	I-III	NSCLC	Cancer-free	miR-486, -210	Plasma	49	13	10	46	83.10%	78.00%	0.89
**2015.Wang, P.(a)**	142/111	I-III	LC	HC	miR-125a-5p, -25, -126	Serum	125	19	17	92	88.00%	82.60%	0.93
**2015.Wang, P.(b)**	94/111	I-II	LC	HC	miR-125a-5p, -25, -126	Serum	82	14	12	97	87.50%	87.50%	0.94
**2014.Li, N.**	35/40	NA	NSCLC	Cancer-free	miR-31, -210	Sputum	23	6	12	34	65.71%	85.00%	0.86
**2013.Tang, D.[a]**	62/60	I-III	NSCLC	HC	miR-145, -155	Plasma	44	13	18	47	71.00%	78.30%	0.82
**2013.Tang, D.[b]**	62/60	I-III	NSCLC	HC	miR-21, -145, -155	Plasma	43	13	19	47	69.40%	78.30%	0.85
**2013.Tang, D.[c]**	62/60	I-III	NSCLC	HC	miR-21, -155	Plasma	39	13	23	47	62.90%	78.30%	0.76
**2013.Tang, D.[d]**	62/60	I-III	NSCLC	HC	miR-21, -145	Plasma	41	17	21	43	66.10%	71.70%	0.81
**2013.Tang, D.[e]**	40/60	I-II	AD	HC	miR-21, -145, -155	Plasma	28	13	12	47	70.60%	78.30%	NS
**2013.Tang, D.[f]**	9/60	I-II	SCC	HC	miR-21, -145, -155	Plasma	6	13	3	47	66.70%	78.30%	NS
**2013.Tang, D.[g]**	34/32	I-III	NSCLC	HC	miR-21, -145, -155	Plasma	26	6	8	26	76.50%	81.30%	0.87
**2013.Tang, D.[h]**	34/32	I-III	NSCLC	HC	miR-145, -155	Plasma	27	7	7	25	79.40%	78.10%	0.83
**2013.Tang, D.[i]**	34/32	I-III	NSCLC	HC	miR-21, -145	Plasma	25	6	9	26	73.50%	81.30%	0.85
**2013.Tang, D.[j]**	34/32	I-III	NSCLC	HC	miR-21, -155	Plasma	19	11	15	21	55.90%	65.60%	0.73
**2013.Anjuman, N.**	43/47	I	NSCLC	Cancer-free	miR-31, -210	Sputum	26	4	17	43	61.50%	90.50%	0.83
**2011.Shen, J[2].[a]**	76/80	I-IV	NSCLC	Cancer-free	miR-21, -210, -486-5p	Sputum	58	12	18	68	76.32%	85.00%	NS
**2011.Shen, J[2].[b]**	40/80	I-IV	AD	Cancer-free	miR-21, -210, -486-5p	Sputum	30	12	10	68	75.00%	85.00%	NS
**2011.Shen, J[2].[c]**	36/80	I-IV	SCC	Cancer-free	miR-21, -210, -486-5p	Sputum	28	12	8	68	77.78%	85.00%	NS
**2011.Shen, J[2].[d]**	30/80	II	NSCLC	Cancer-free	miR-21, -210, -486-5p	Sputum	24	12	6	68	80.00%	85.00%	NS
**2011.Shen, J[2].[e]**	24/80	I	NSCLC	Cancer-free	miR-21, -210, -486-5p	Sputum	18	12	6	68	75.00%	85.00%	NS
**2011.Shen, J[2].[f]**	22/80	III-IV	NSCLC	Cancer-free	miR-21, -210, -486-5p	Sputum	16	12	6	68	72.73%	85.00%	NS
**2011.Shen, J[2].[g]**	64/73	I-IV	LC	Cancer-free	miR-31, -210	Sputum	41	8	23	65	64.10%	89.20%	NS
**2011.Shen, J[2].[h]**	66/68	I-IV	LC	Cancer-free	miR-31, -210	Sputum	43	7	23	61	65.20%	89.70%	0.83

(1), (2): Different articles by the same author in the same year; (a), (b): Different diagnosis data in the same article; N/S: not specified.

AD: Adenocarcinoma; SCC: Squamous cell carcinoma; NSCLC: Non-Small Cell Lung Carcinoma; LC: lung cancer; HC: health control; EBC: exhaled breath condensate; PLF: Pleural lavage fluid; PB: Peripheral blood; PBMCs: Peripheral blood mononuclear cells; BALF: bronchoalveolar lavage; LT: Lung tissue; TP: true positive; FP: false positive; FN: false negative; TN: true negative; AUC: Area Under Curve; Sen: sensitivity; Spe: Specificity.
