# Supplementary material for: High diagnostic value of miRNAs for NSCLC: quantitative analysis for both single and combined miRNAs in lung cancer
Source: Ann Med. 2021 Dec 16;53(1):2178–93. doi: 10.1080/07853890.2021.2000634 (PMC8740622; doi:10.1080/07853890.2021.2000634)
Supplement: Supplemental Material [file IANN_A_2000634_SM6817.zip › Supplemental files/20210918_Appendix 2_Figures.pdf]

Supplementary material 2: Appendix Figures.

Appendix Figure 1. Quality assessment of 80 quantitative studies through the tool of Quality Assessment of Diagnostic Accuracy Studies-2 (QUADAS-2).

Appendix Figure 2. Funnel plots and Deek plots of publication bias for 20 miRNAs in NSCLC diagnosis.

Appendix Figure 3. Forest plots of 4 miRNAs and their diagnostic value in unclassified LC.

Appendix Figure 4. The SROC of 4 miRNAs and the diagnostic value in unclassified LC.

Appendix Figure 5. Forest plots of the association between 20 involved single-miRNAs and their diagnostic value in NSCLC.

Appendix Figure 6. The SROC of 20 involved single-miRNAs and their diagnostic value in NSCLC.

Appendix Figure 7. Forest plots of the association between involved single-miRNAs and their diagnostic value in early-stage NSCLC.

Appendix Figure 8. The SROC of single-miRNAs describing their diagnostic value in early-stage NSCLC.

Appendix Figure 9. Forest plots of miRNA panel1 and two models and their diagnostic value in NSCLC.

Appendix Figure 10. The SROC of miRNA panel1 and two models describing their diagnostic value in NSCLC.

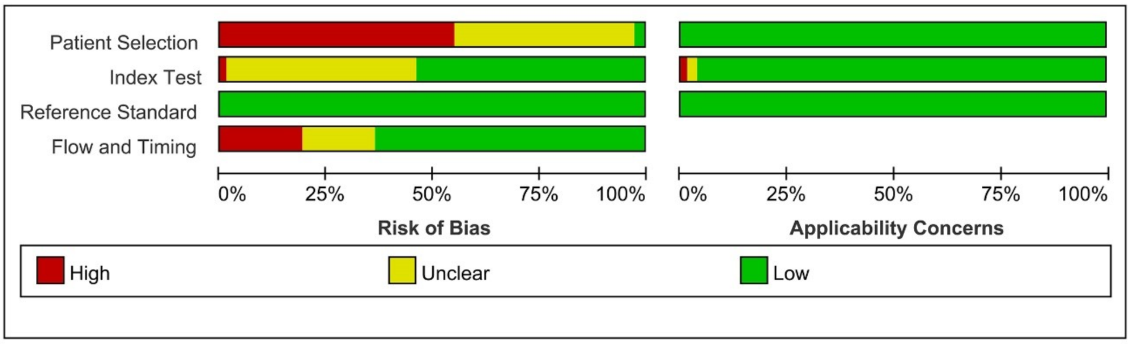

Appendix Figure 1. Quality assessment of 80 quantitative studies through the tool of Quality Assessment of Diagnostic Accuracy Studies-2 (QUADAS-2).

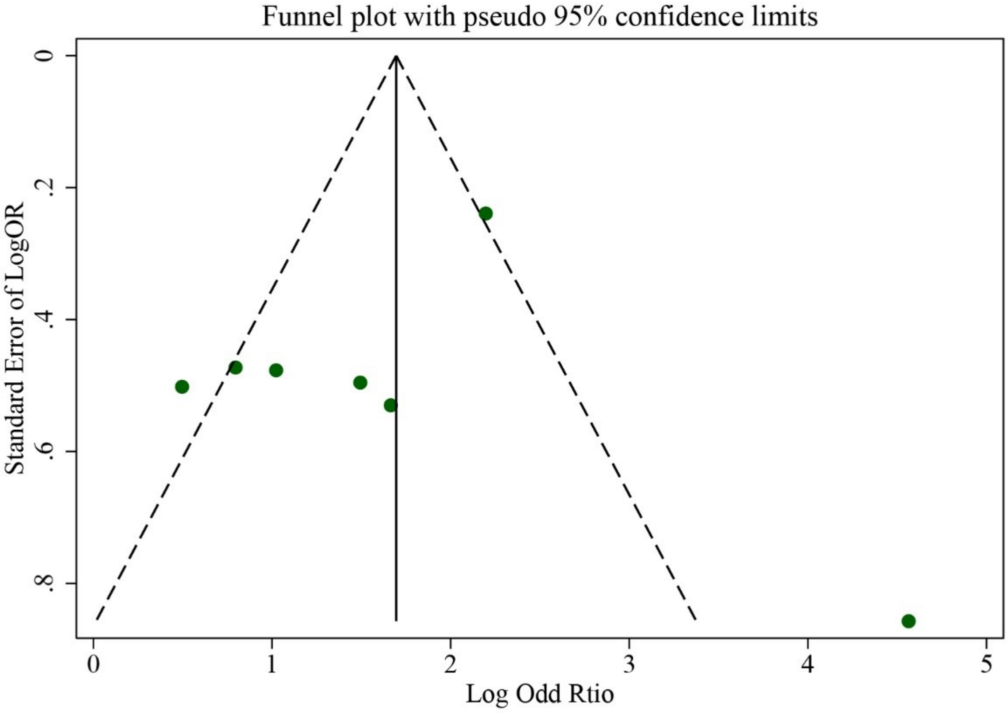

Appendix Figure 2a. Funnel plot of publication bias for Let-7 in NSCLC diagnosis.

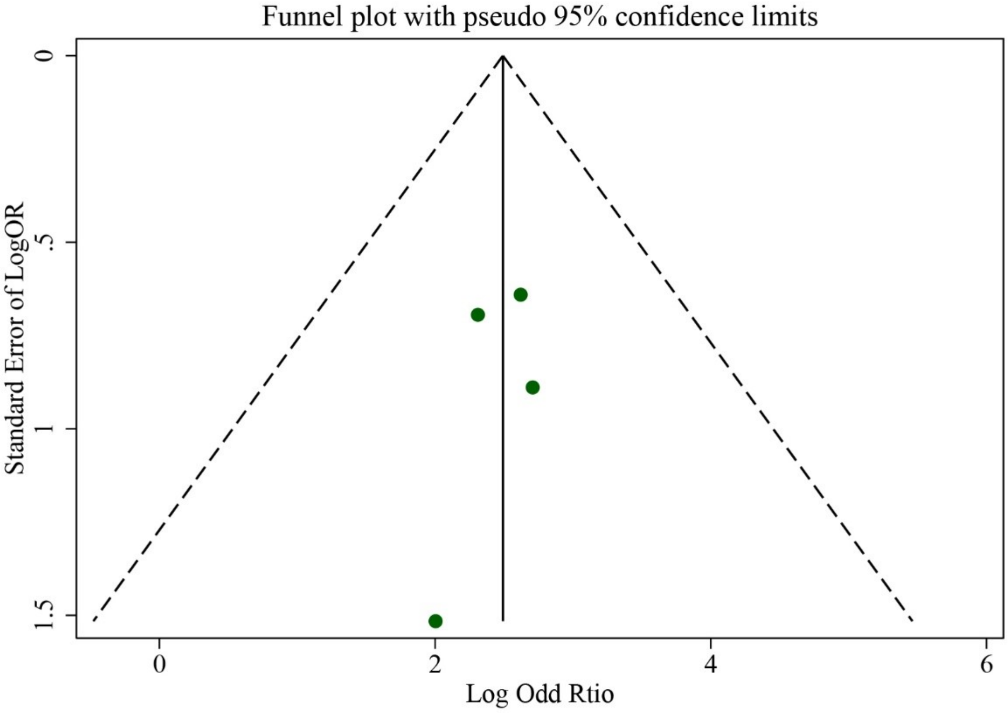

Appendix Figure 2b. Funnel plot of publication bias for miR-7 in NSCLC diagnosis.

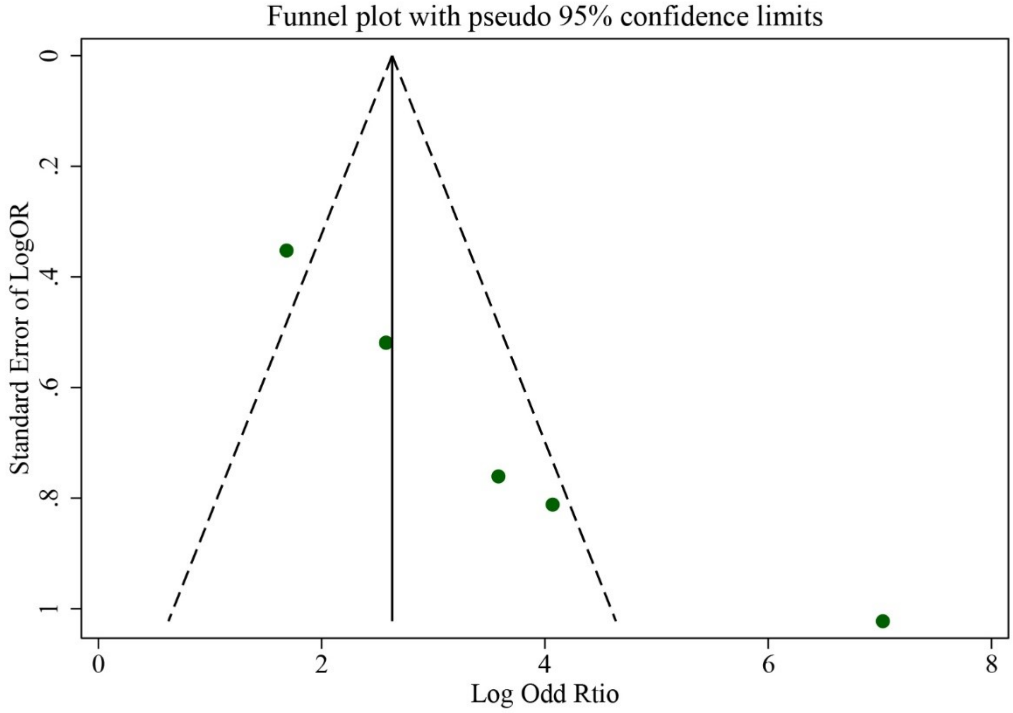

Appendix Figure 2c. Funnel plot of publication bias for miR-10 in NSCLC diagnosis.

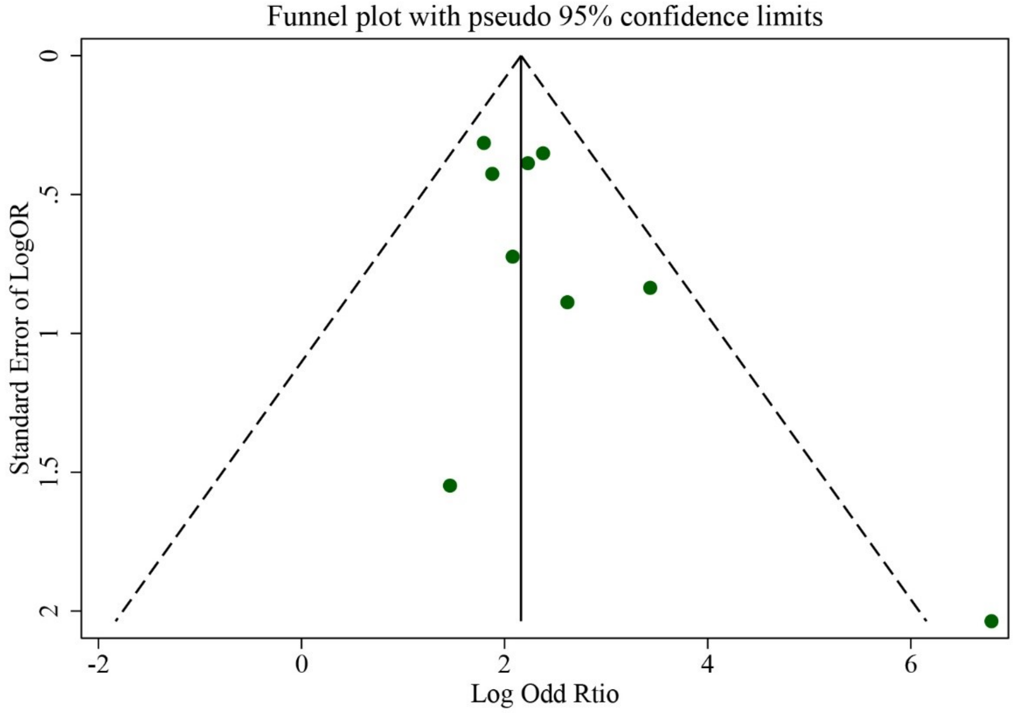

Appendix Figure 2d. Funnel plot of publication bias for miR-17 in NSCLC diagnosis.

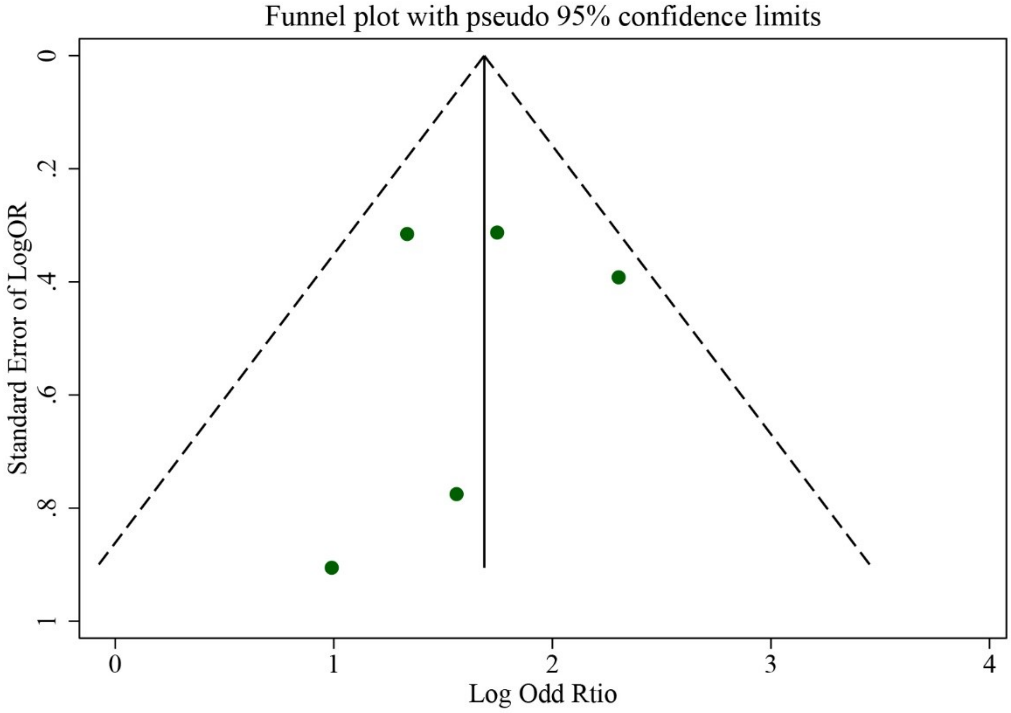

Appendix Figure 2e. Funnel plot of publication bias for miR-19 in NSCLC diagnosis.

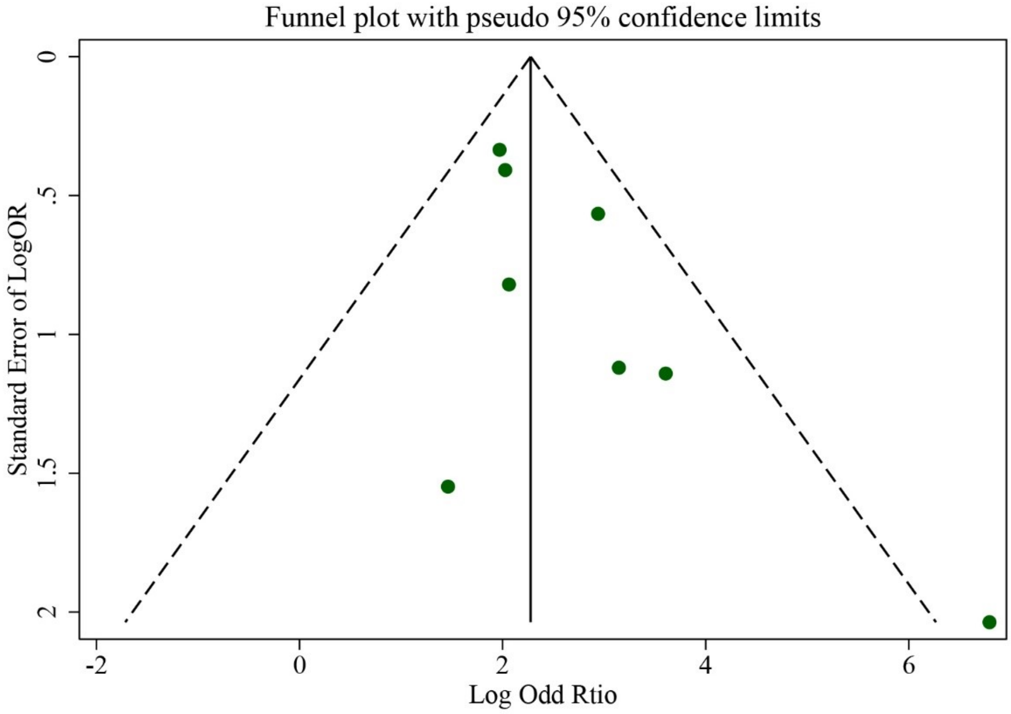

Appendix Figure 2f. Funnel plot of publication bias for miR-20 in NSCLC diagnosis.

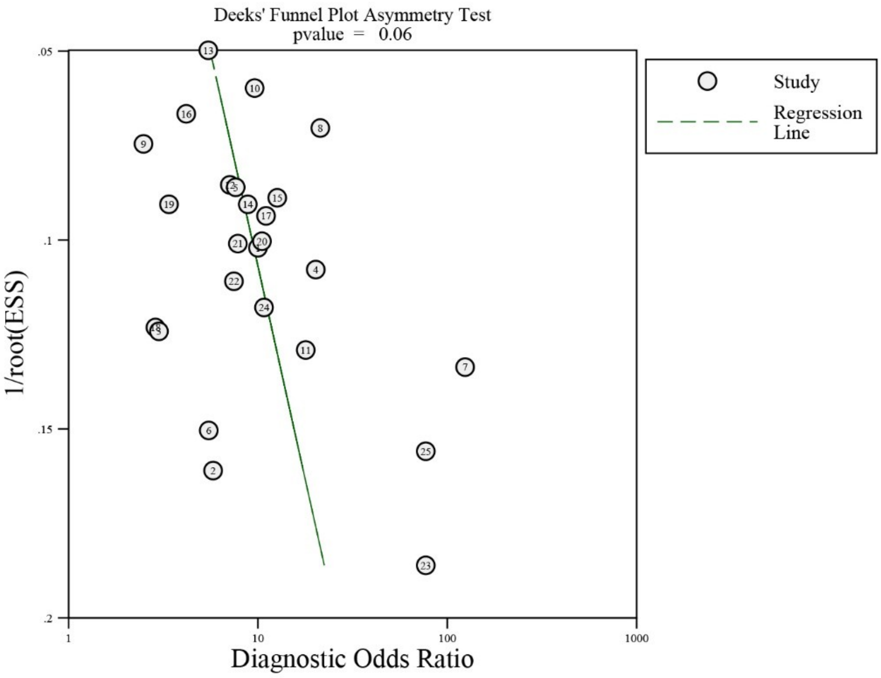

Appendix Figure 2g. Deek plot of publication bias for miR-21 in NSCLC diagnosis.

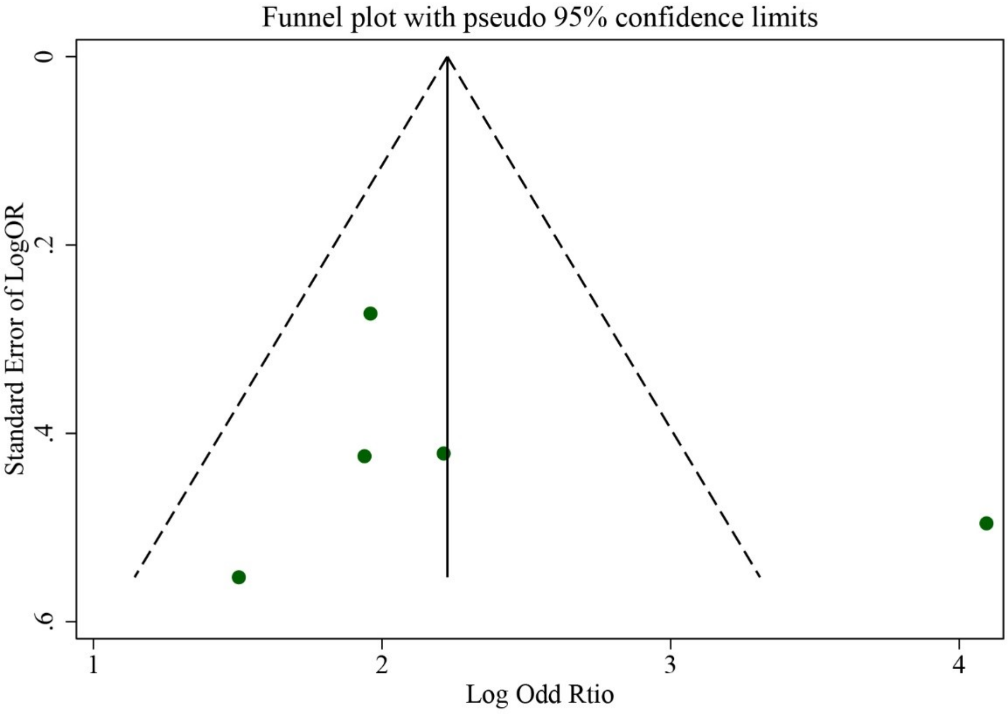

Appendix Figure 2h. Funnel plot of publication bias for miR-31 in NSCLC diagnosis.

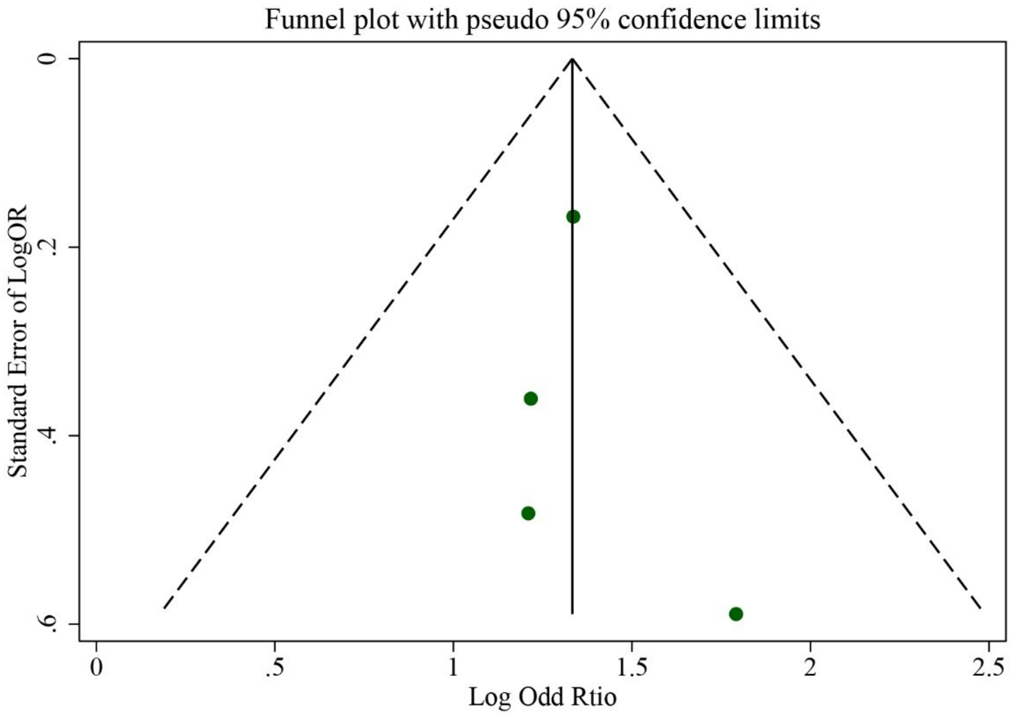

Appendix Figure 2i. Funnel plot of publication bias for miR-125 in NSCLC diagnosis.

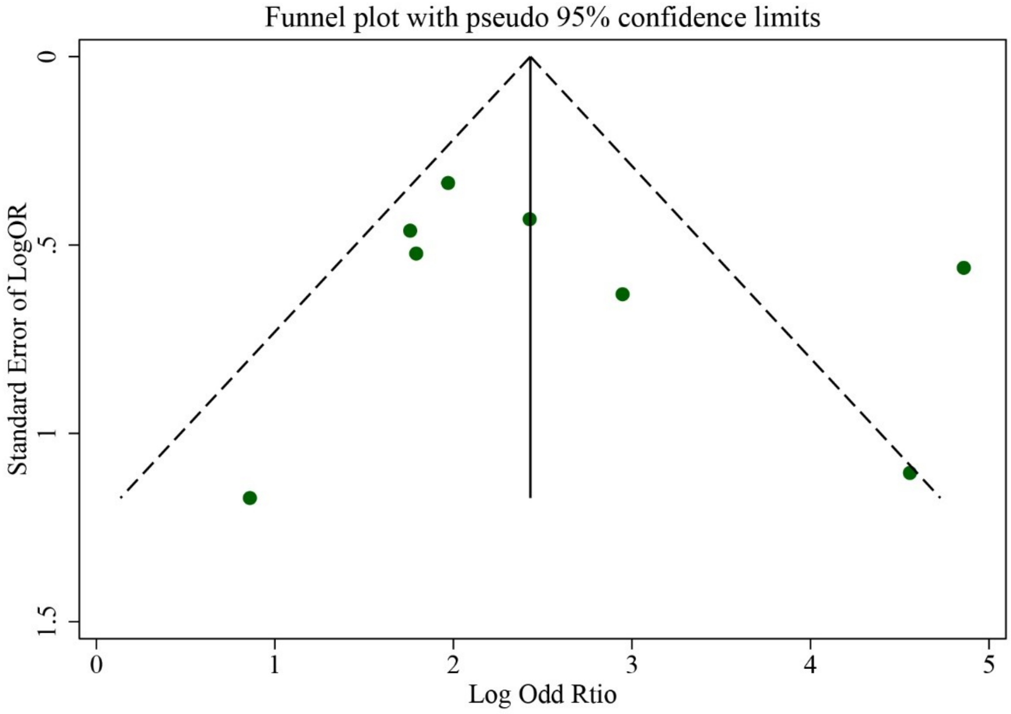

Appendix Figure 2j. Funnel plot of publication bias for miR-126 in NSCLC diagnosis.

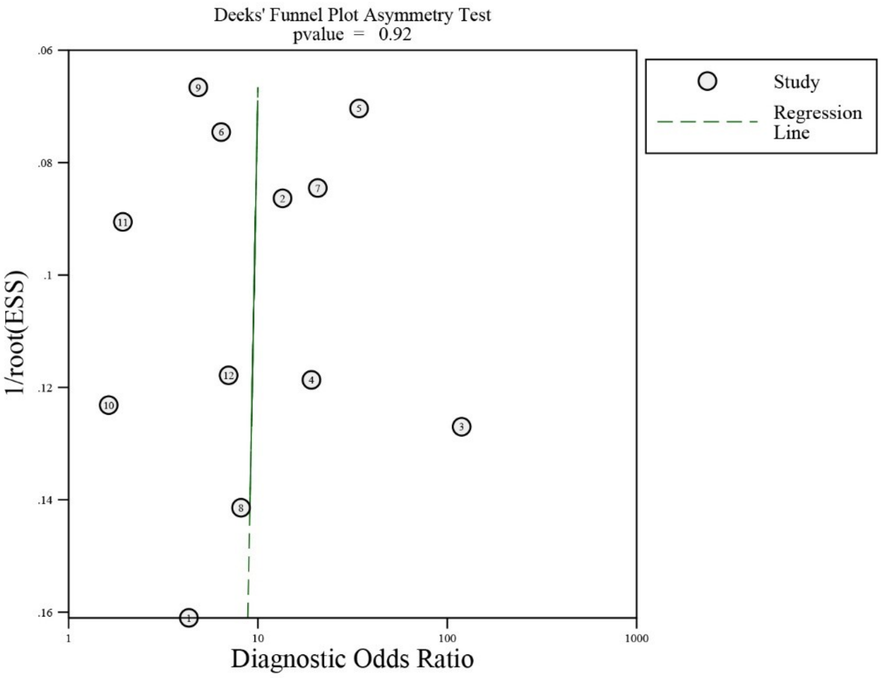

Appendix Figure 2k. Deek plot of publication bias for miR-145 in NSCLC diagnosis.

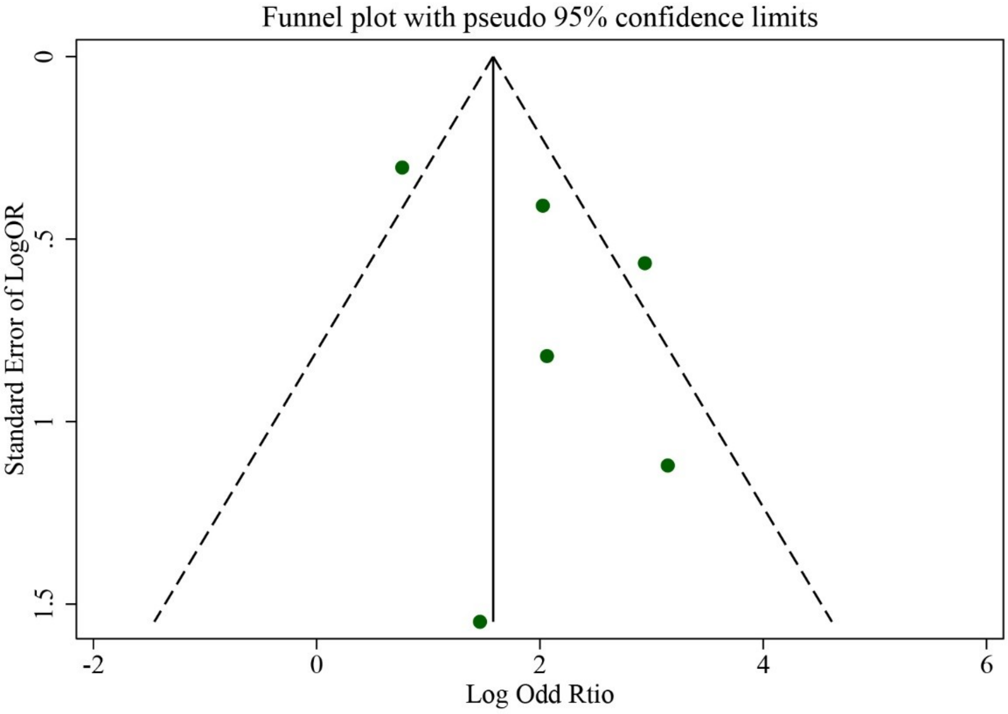

Appendix Figure 2l. Funnel plot of publication bias for miR-146 in NSCLC diagnosis.

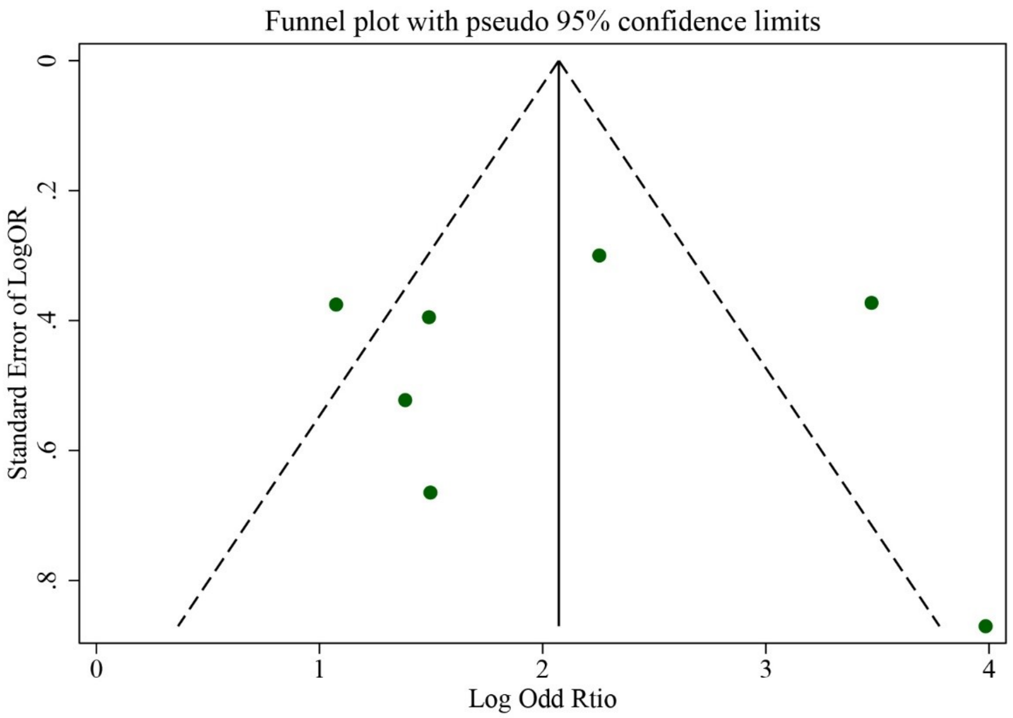

Appendix Figure 2m. Funnel plot of publication bias for miR-155 in NSCLC diagnosis.

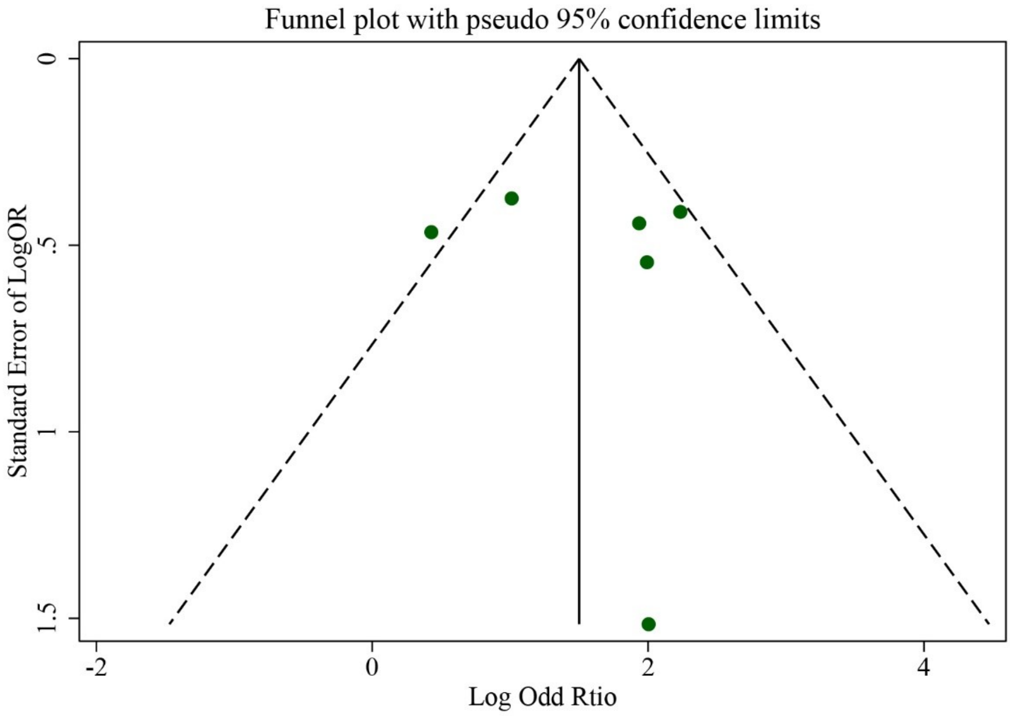

Appendix Figure 2n. Funnel plot of publication bias for miR-182 in NSCLC diagnosis.

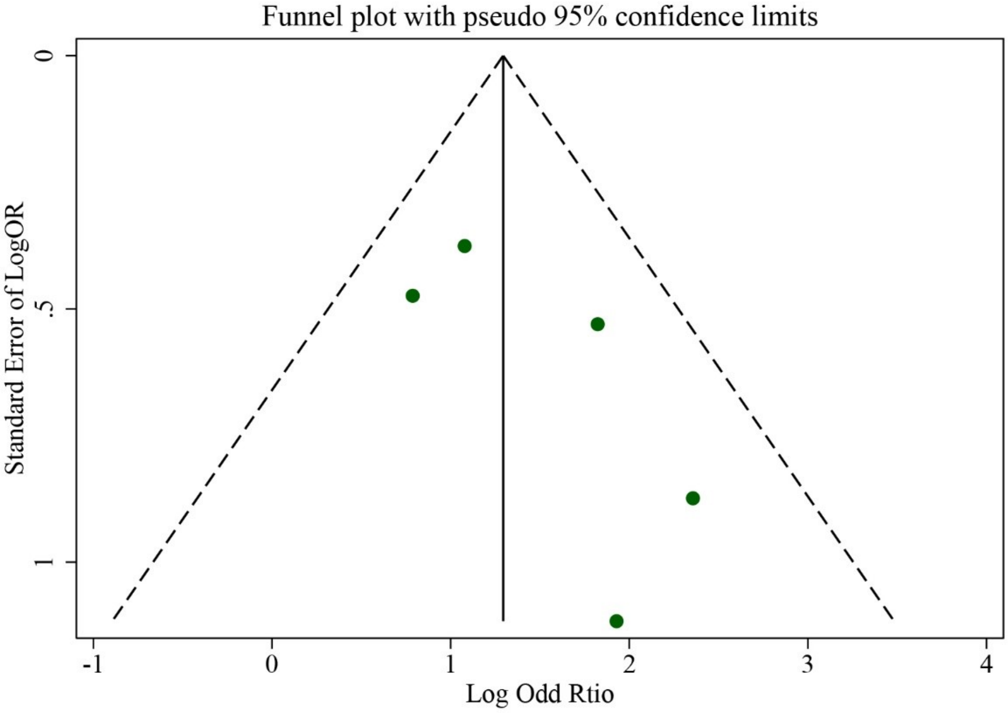

Appendix Figure 2o. Funnel plot of publication bias for miR-200 in NSCLC diagnosis.

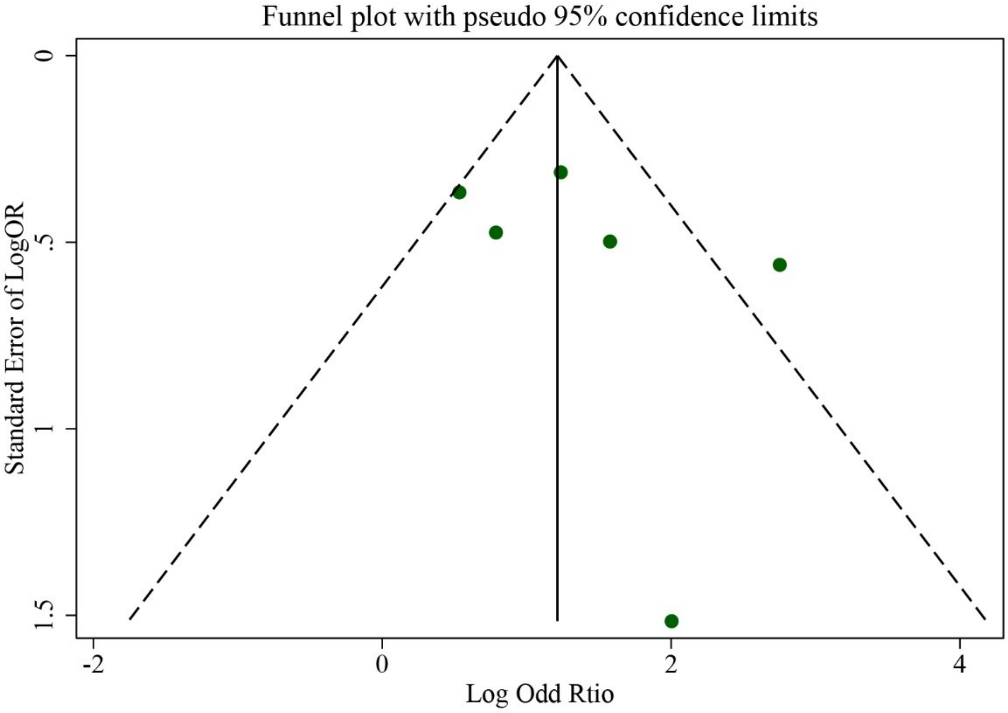

Appendix Figure 2p. Funnel plot of publication bias for miR-205 in NSCLC diagnosis.

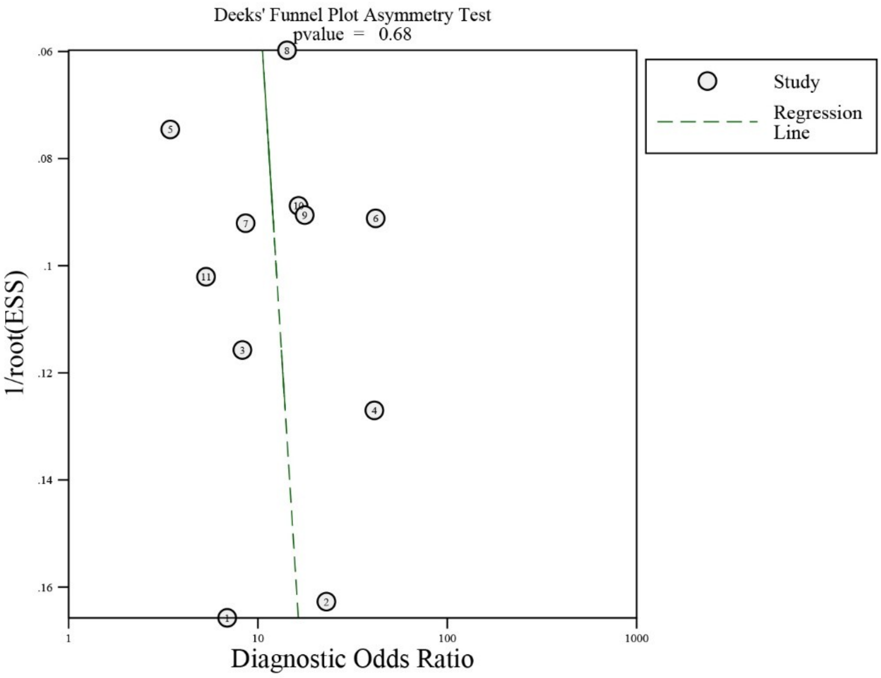

Appendix Figure 2q. Deek plot of publication bias for miR-210 in NSCLC diagnosis.

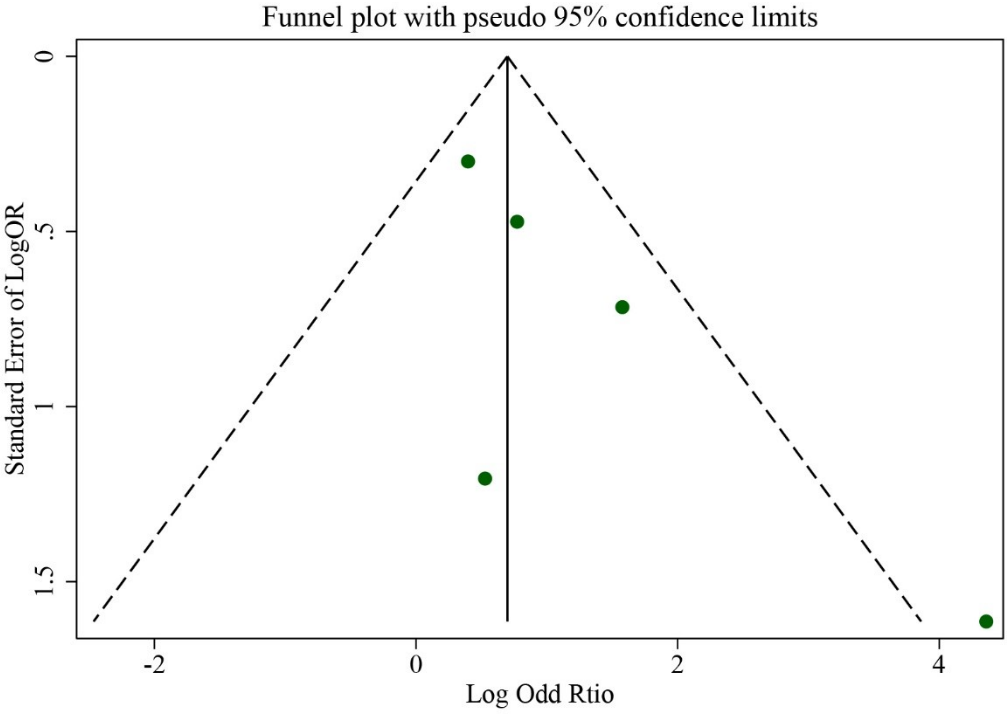

Appendix Figure 2r. Funnel plot of publication bias for miR-221 in NSCLC diagnosis.

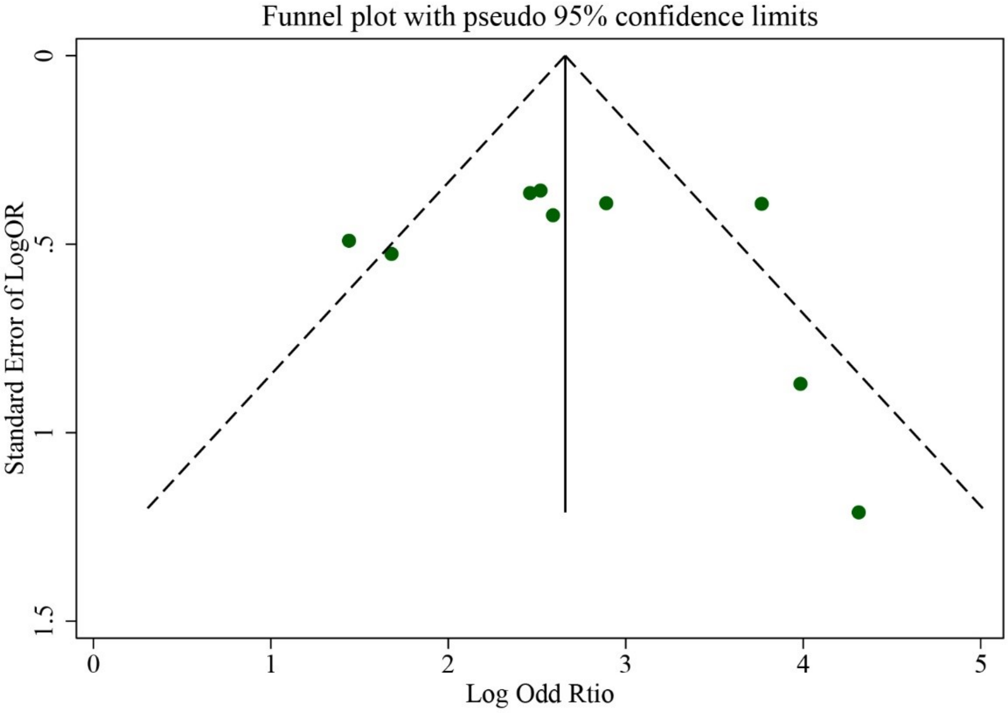

Appendix Figure 2s. Funnel plot of publication bias for miR-223 in NSCLC diagnosis.

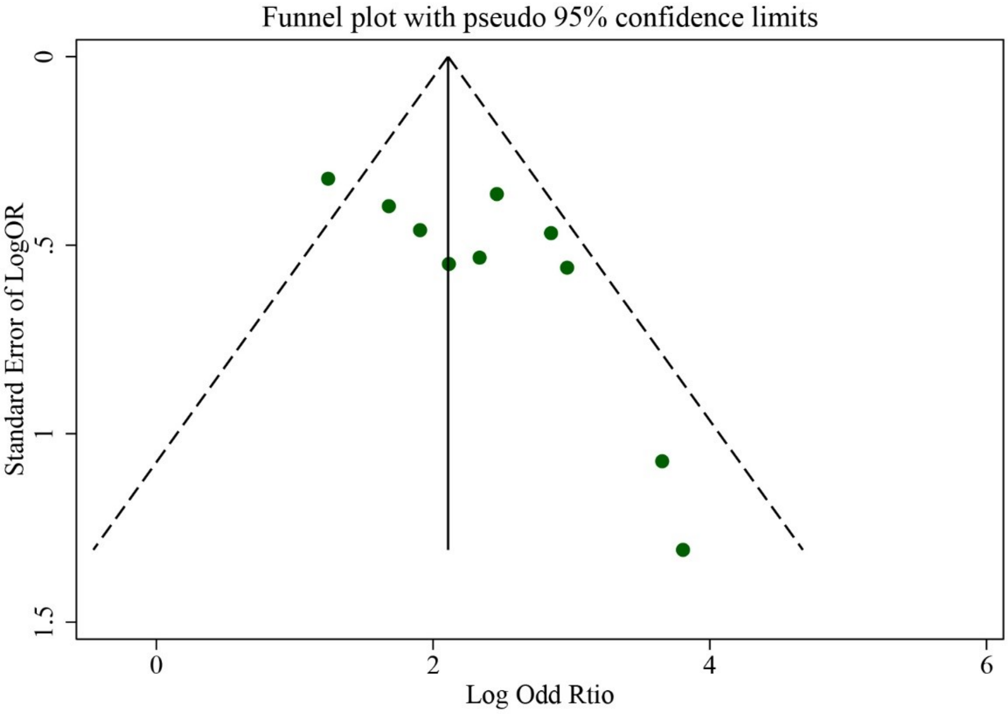

Appendix Figure 2t. Funnel plot of publication bias for miR-486 in NSCLC diagnosis.

Appendix Figure 2. Funnel plots and Deek plots of publication bias for 20 miRNAs in NSCLC diagnosis.

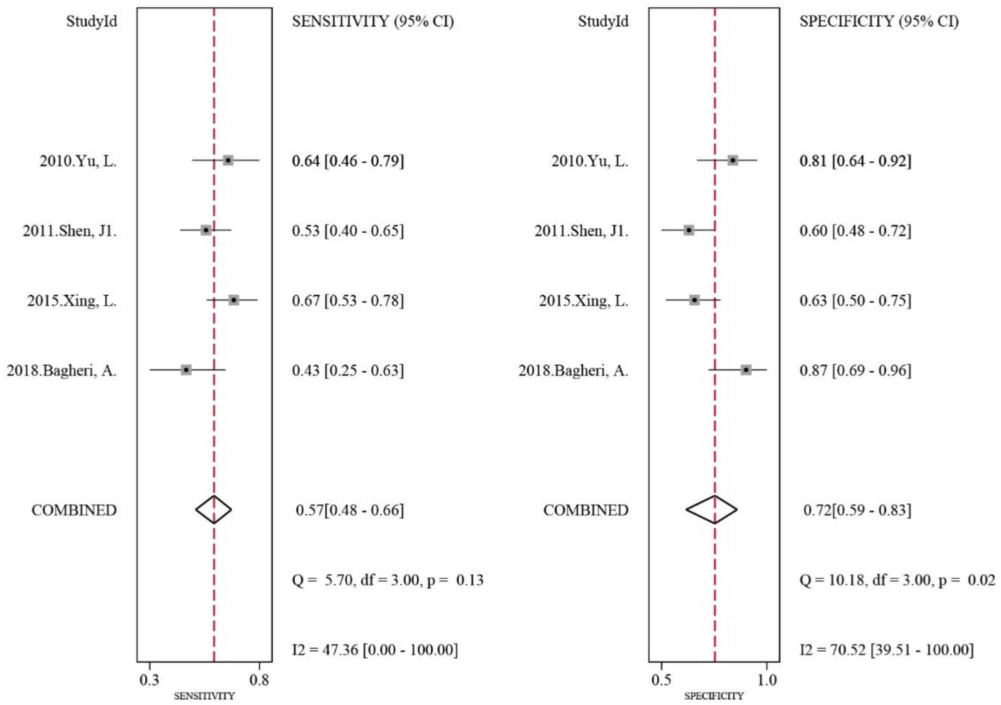

Appendix Figure 3a**.** Forest plots of miR-375 and the diagnostic value in unclassified LC.

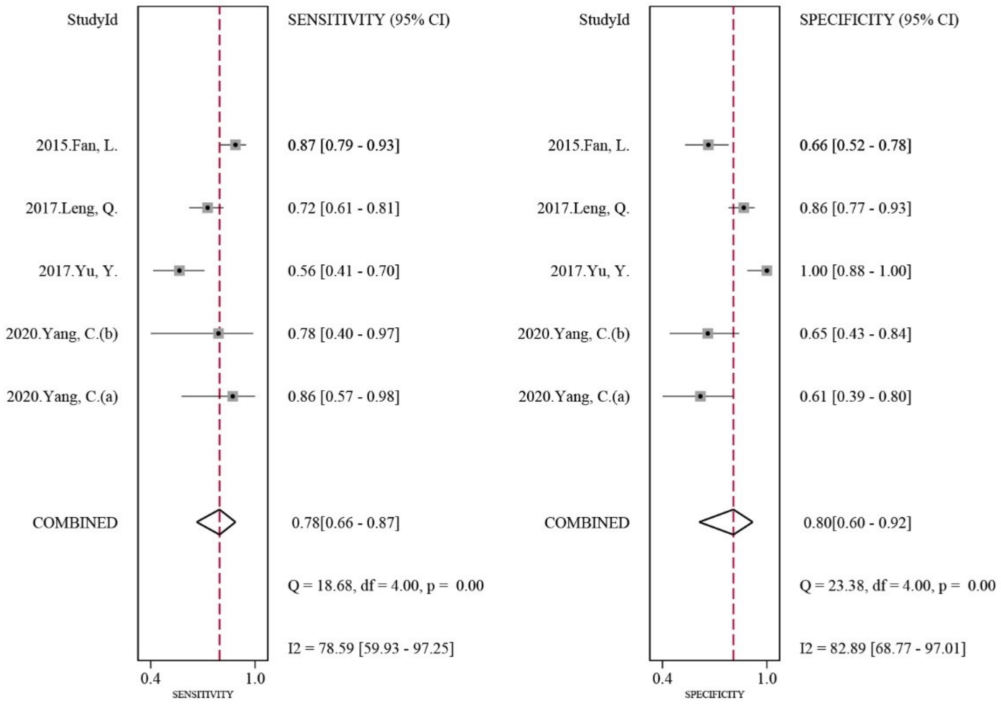

Appendix Figure 3b. Forest plots of miR-92 and the diagnostic value in unclassified LC.

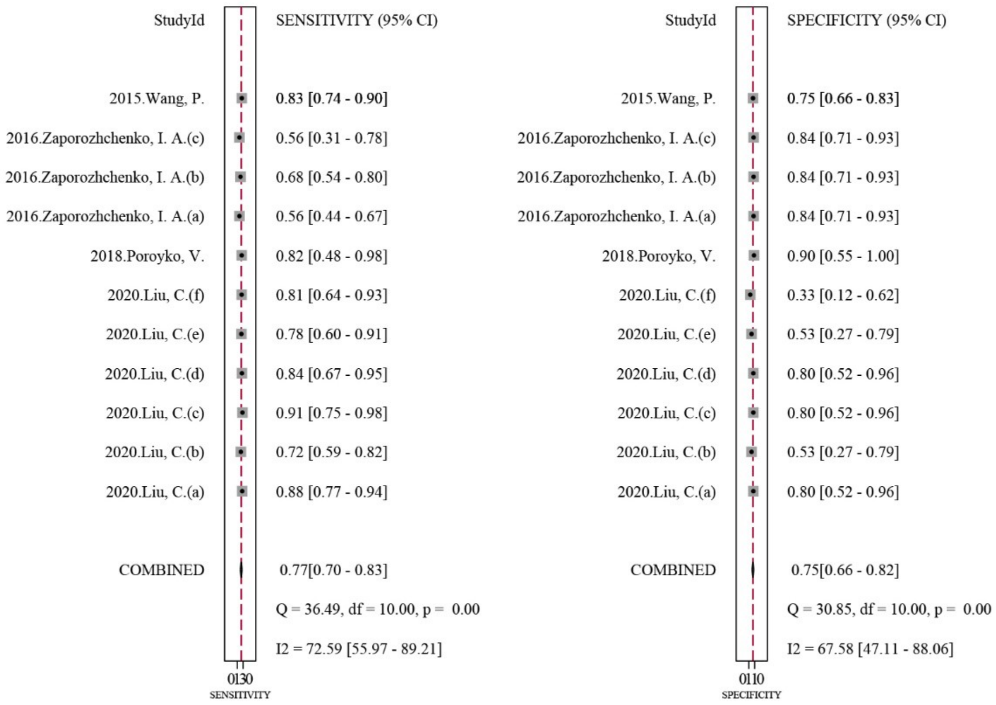

Appendix Figure 3c**.** Forest plots of miR-25 and the diagnostic value in unclassified LC.

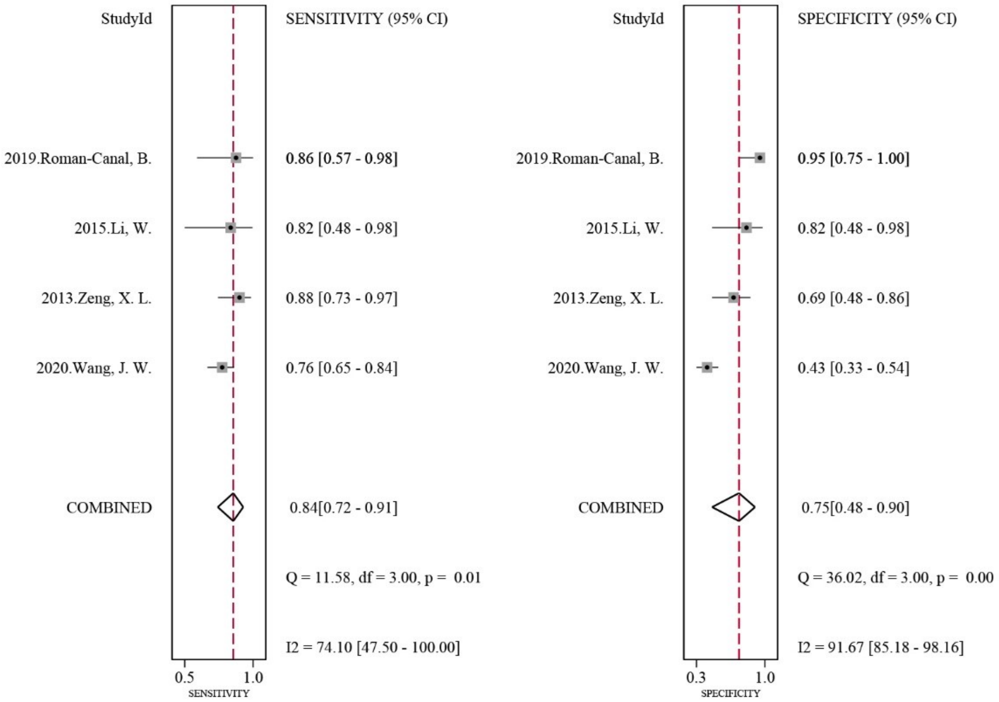

Appendix Figure 3d. Forest plots of miR-150 and the diagnostic value in unclassified LC.

Appendix Figure 3. Forest plots of 4 miRNAs and their diagnostic value in unclassified LC.

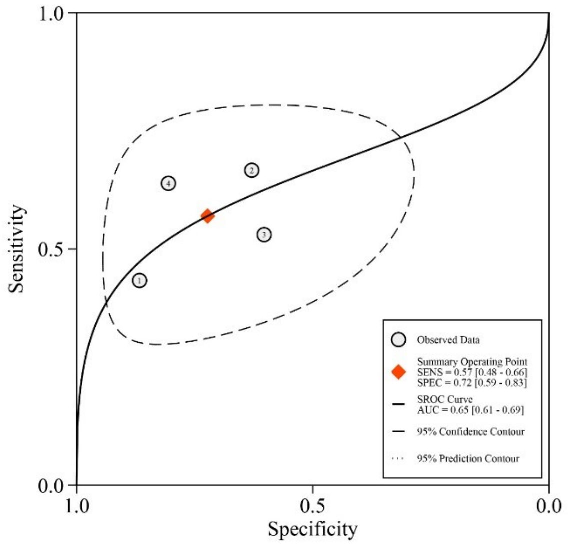

Appendix Figure 4a. The SROC of miR-375 and the diagnostic value in unclassified LC.

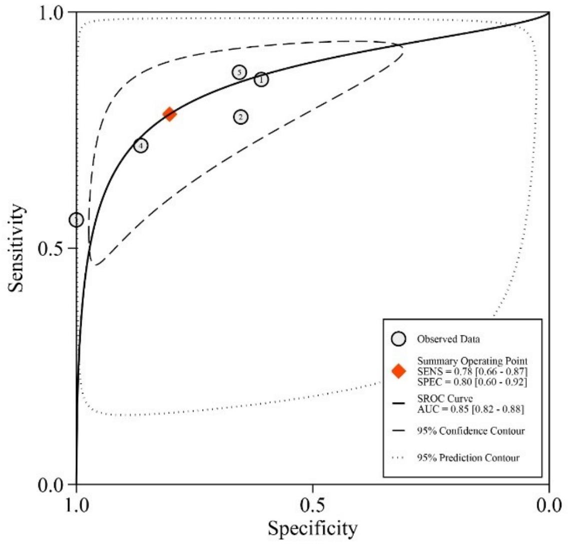

Appendix Figure 4b. The SROC of miR-92 and the diagnostic value in unclassified LC.

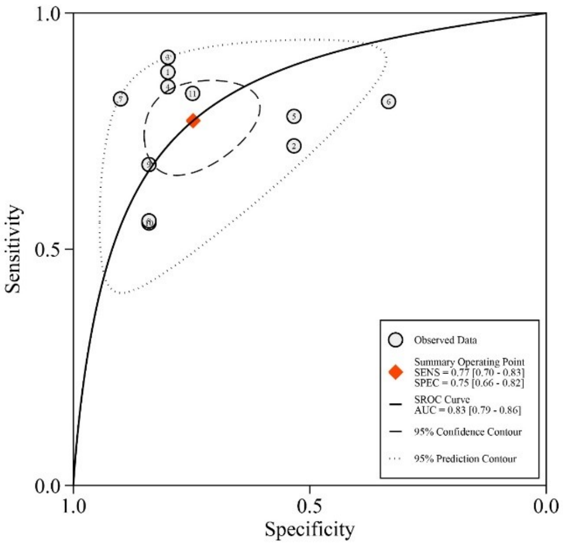

Appendix Figure 4c. The SROC of miR-25 and the diagnostic value in unclassified LC.

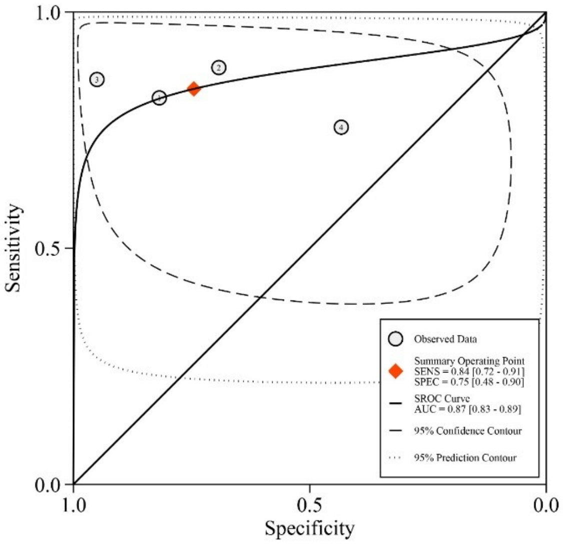

Appendix Figure 4d. The SROC of miR-150 and the diagnostic value in unclassified LC.

Appendix Figure 4. The SROC of 4 miRNAs and the diagnostic value in unclassified LC.

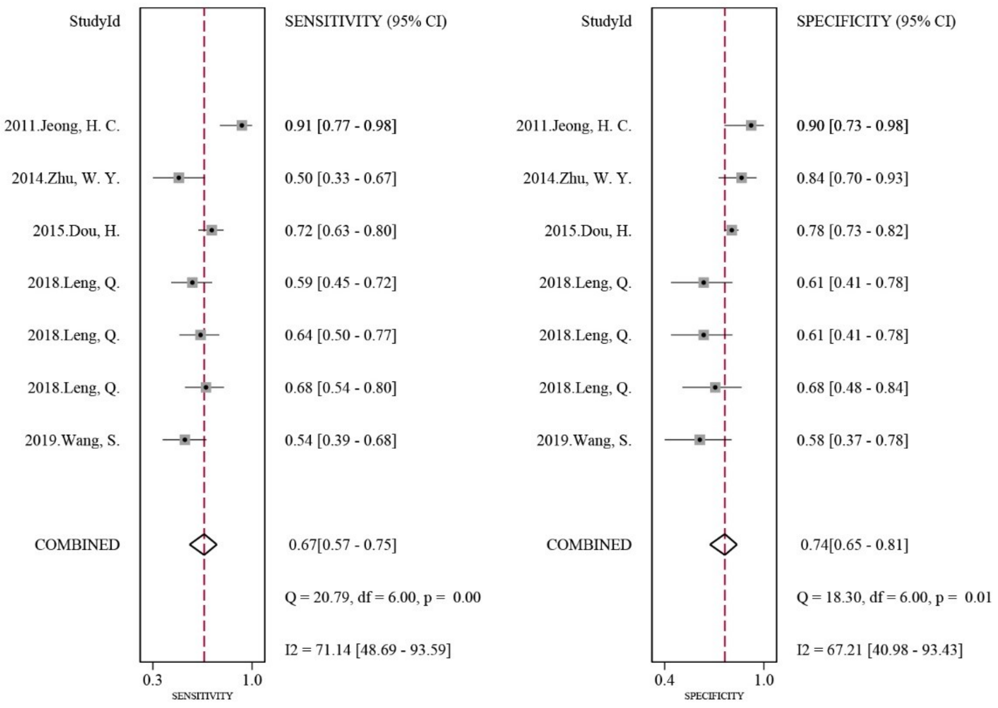

Appendix Figure 5a. Forest plots of the association between Let-7 and the diagnostic value in NSCLC.

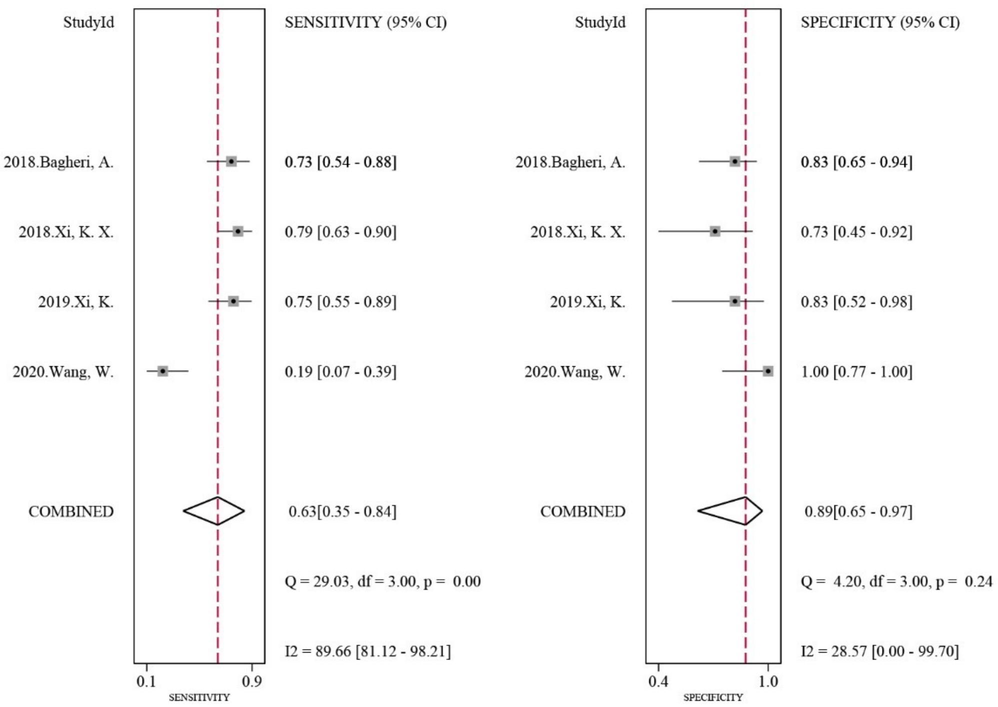

Appendix Figure 5b. Forest plots of the association between miR-7 and the diagnostic value in NSCLC.

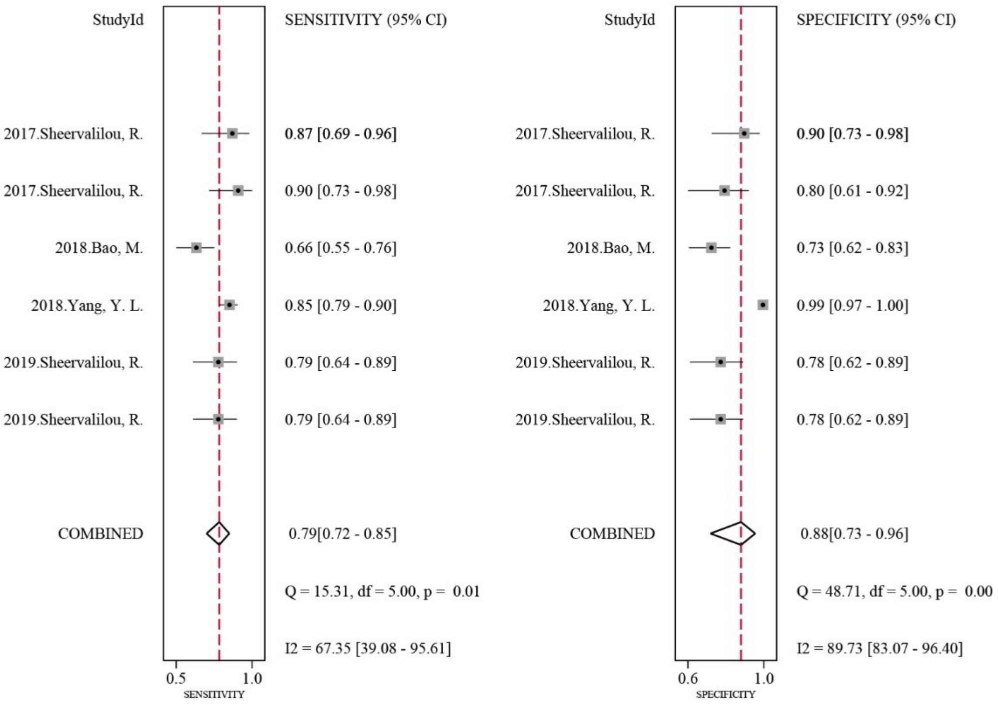

Appendix Figure 5c. Forest plots of the association between miR-10 and the diagnostic value in NSCLC.

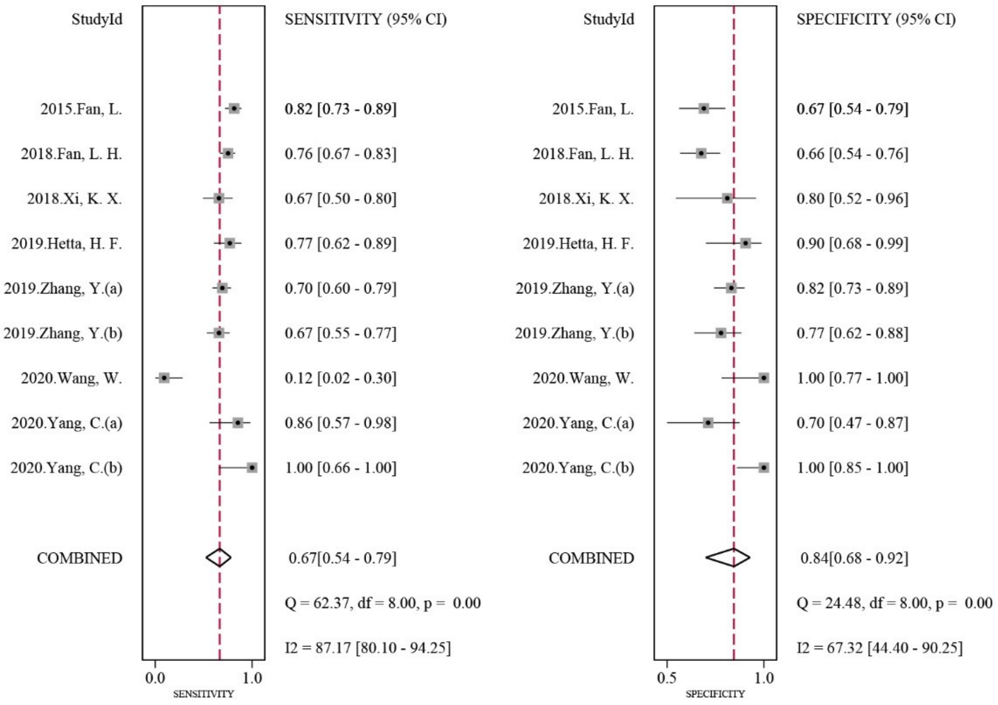

Appendix Figure 5d. Forest plots of the association between miR-17 and the diagnostic value in NSCLC.

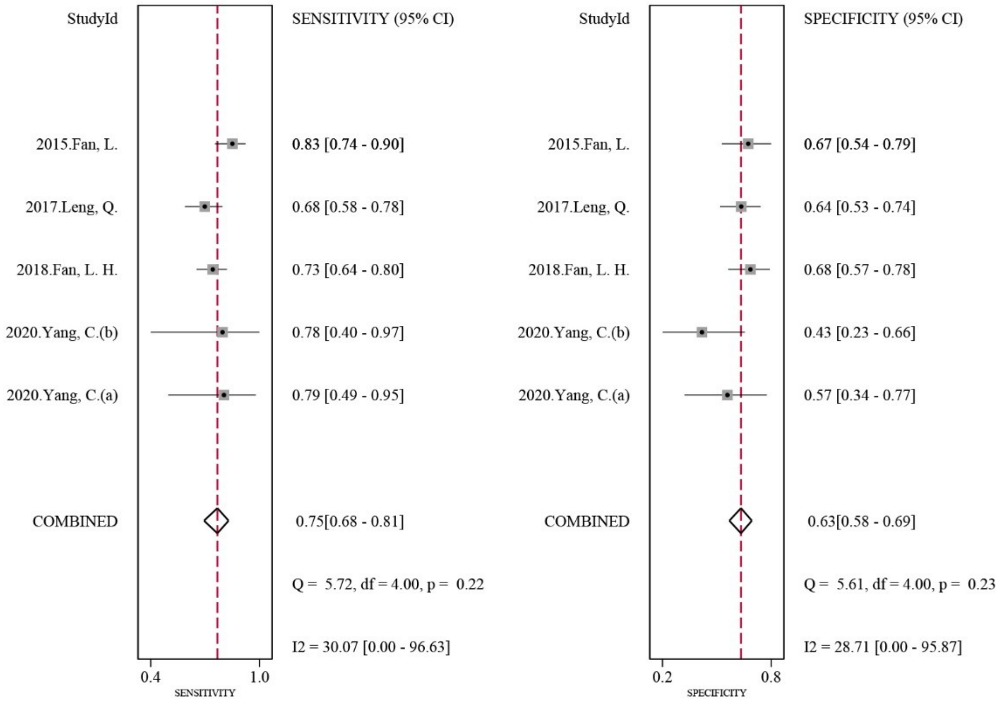

Appendix Figure 5e. Forest plots of the association between miR-19 and the diagnostic value in NSCLC.

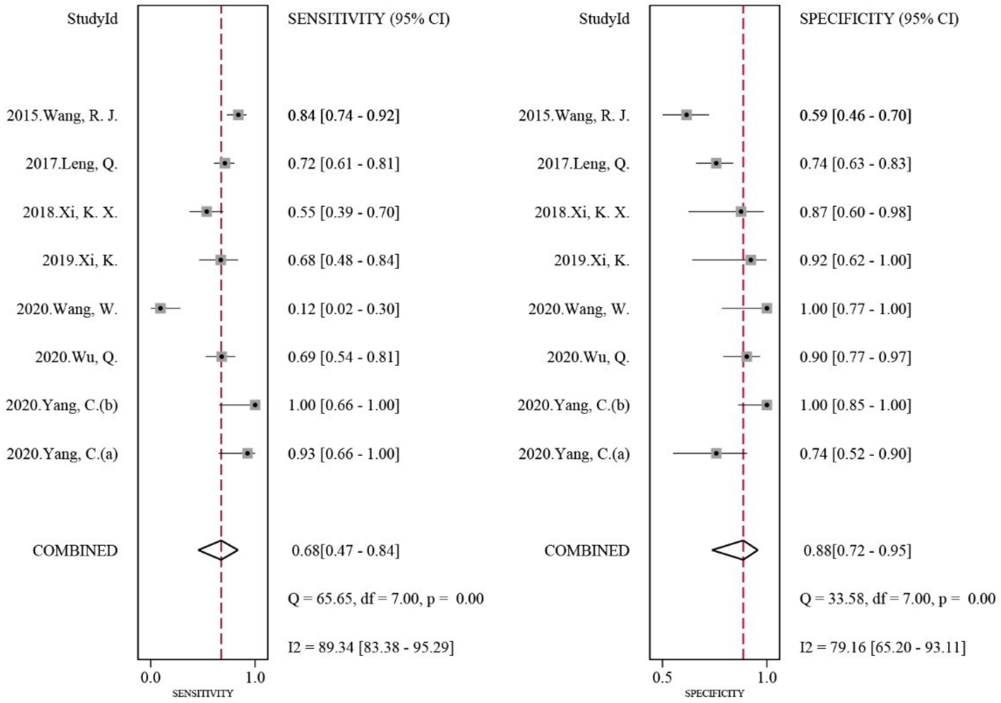

Appendix Figure 5f. Forest plots of the association between miR-20 and the diagnostic value in NSCLC.

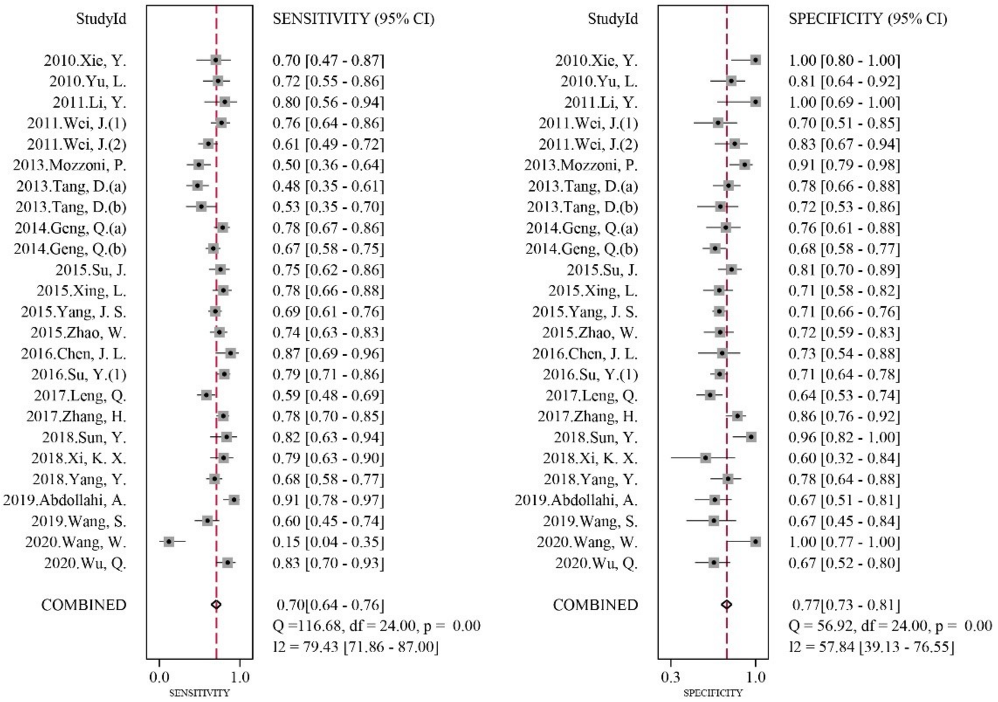

Appendix Figure 5g. Forest plots of the association between miR-21 and the diagnostic value in NSCLC.

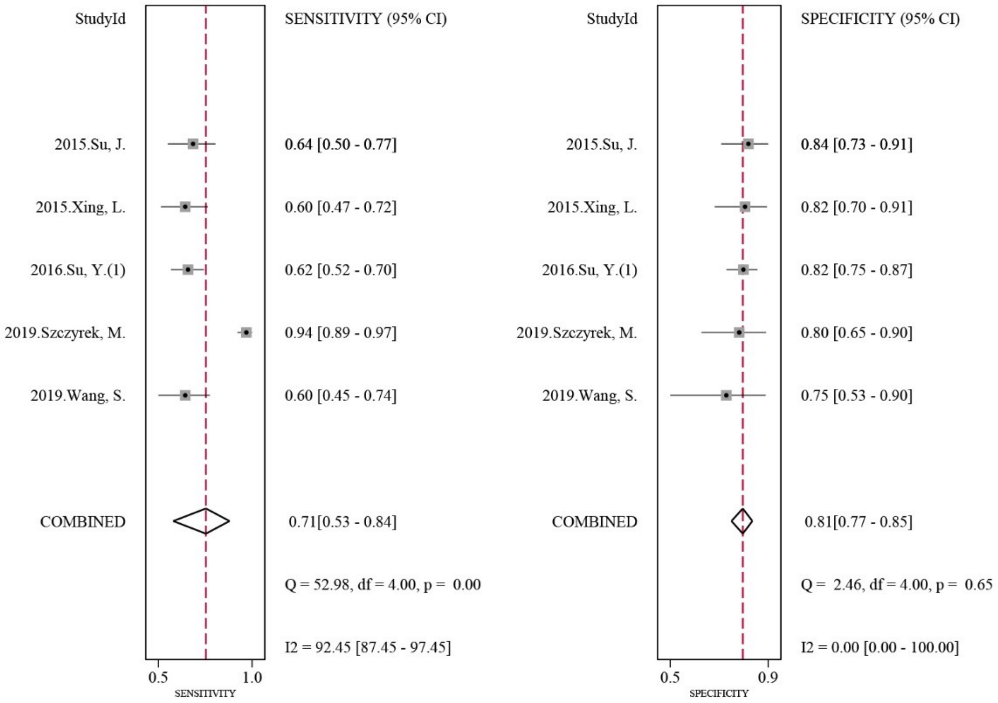

Appendix Figure 5h. Forest plots of the association between miR-31 and the diagnostic value in NSCLC.

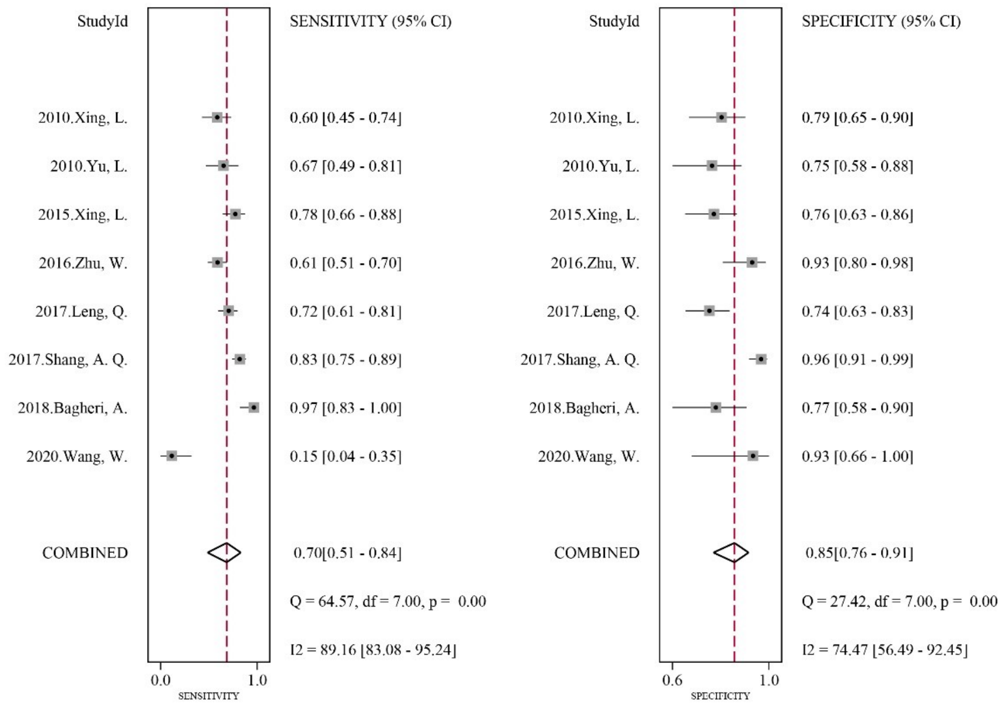

Appendix Figure 5i. Forest plots of the association between miR-126 and the diagnostic value in NSCLC.

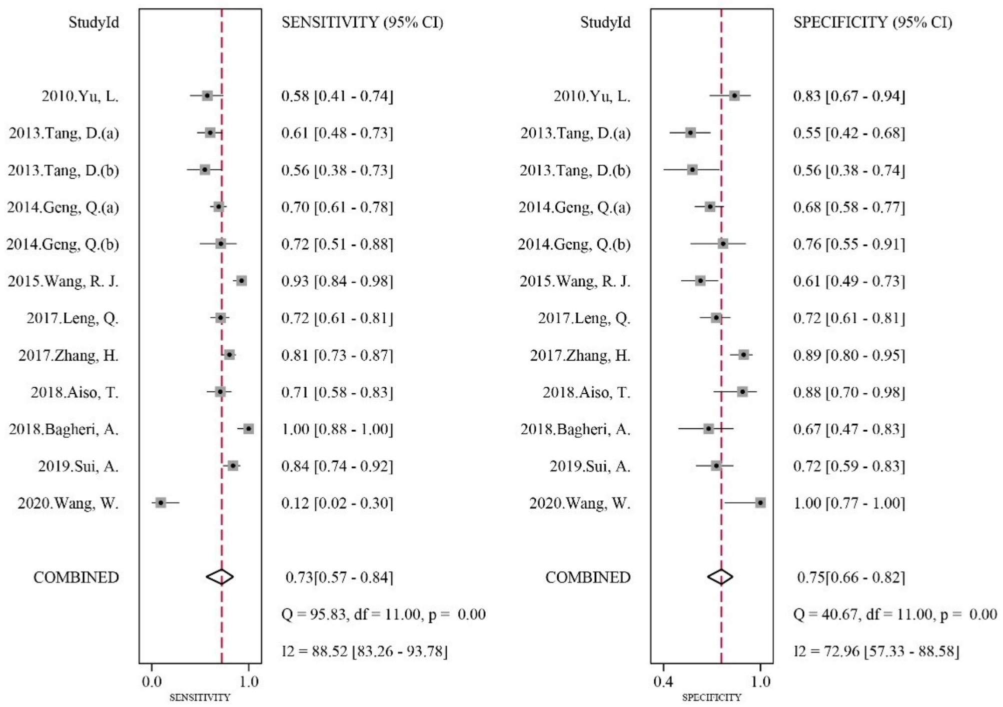

Appendix Figure 5j. Forest plots of the association between miR-145 and the diagnostic value in NSCLC.

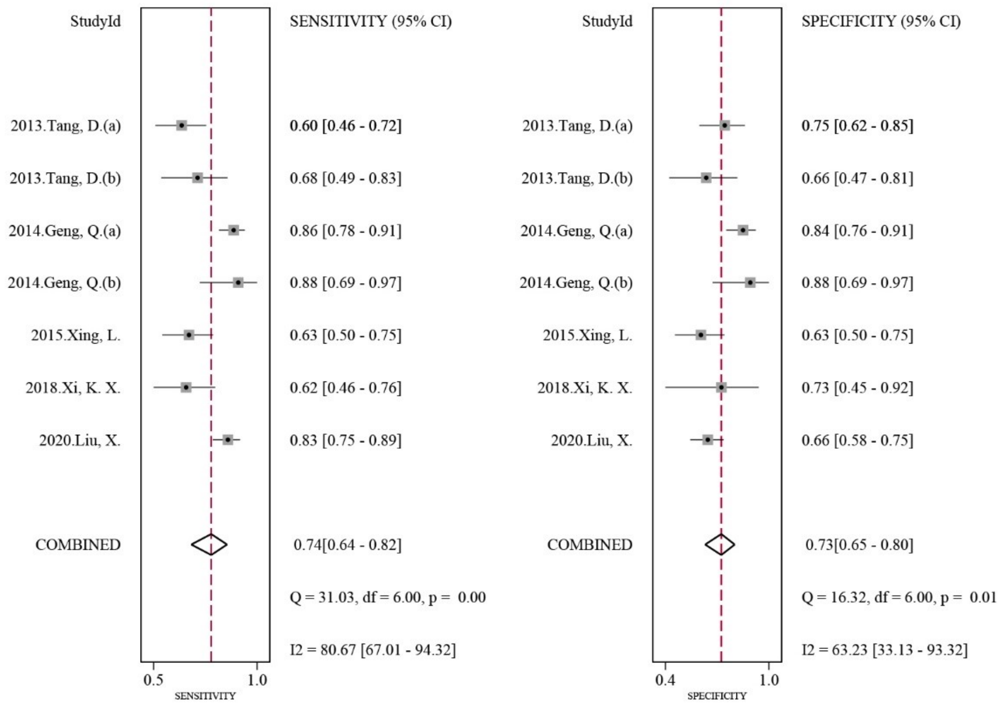

Appendix Figure 5k. Forest plots of the association between miR-155 and the diagnostic value in NSCLC.

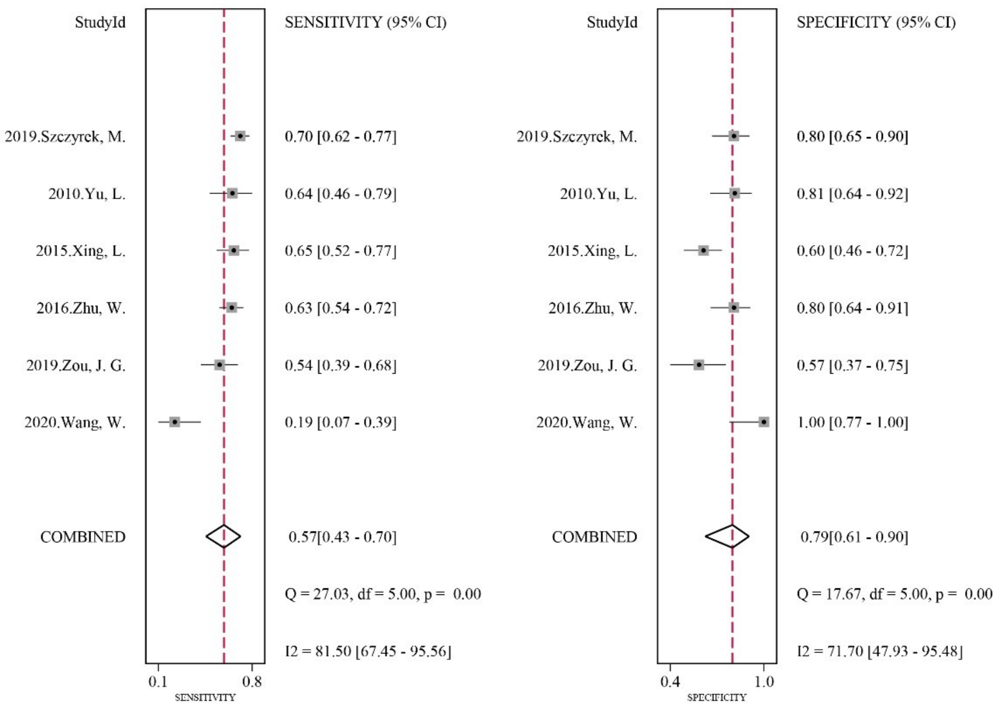

Appendix Figure 5l. Forest plots of the association between miR-182 and the diagnostic value in NSCLC.

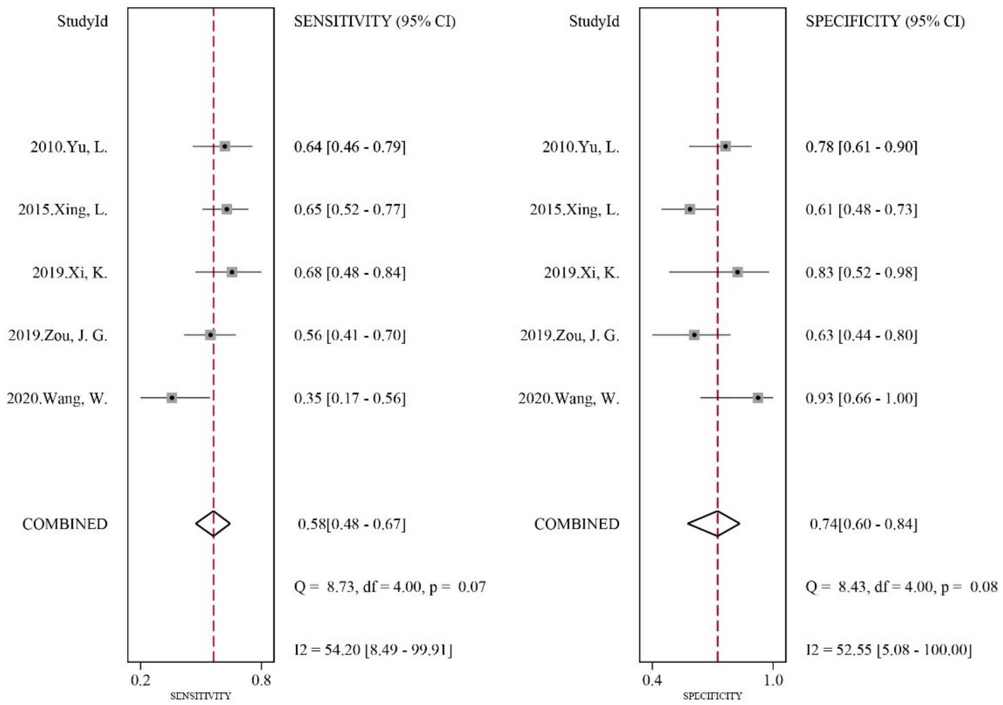

Appendix Figure 5m. Forest plots of the association between miR-200 and the diagnostic value in NSCLC.

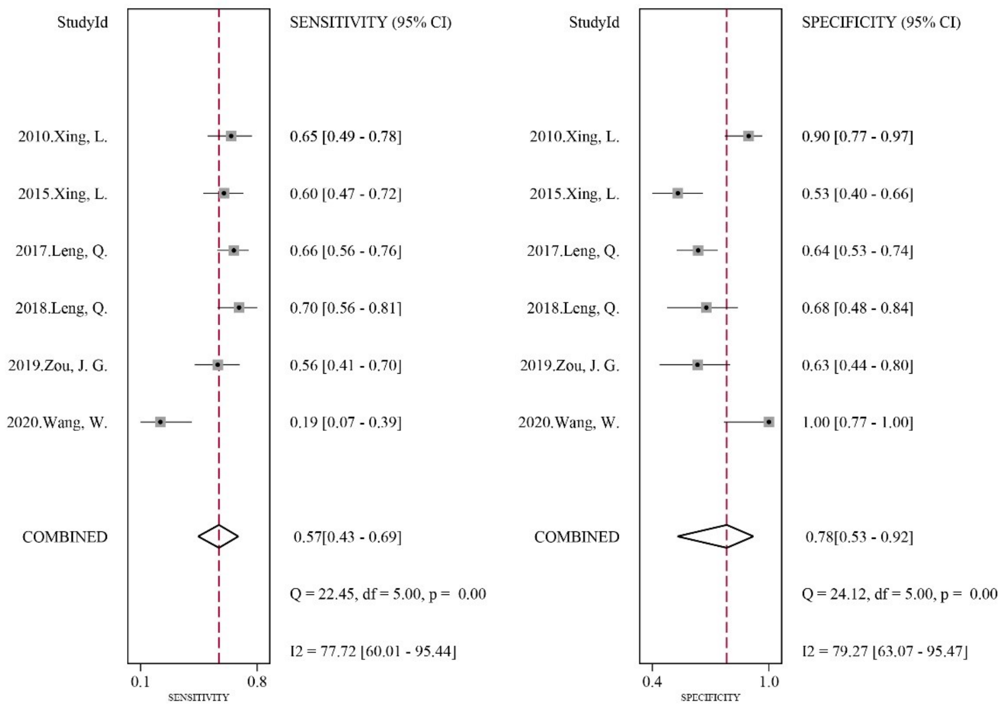

Appendix Figure 5n. Forest plots of the association between miR-205 and the diagnostic value in NSCLC.

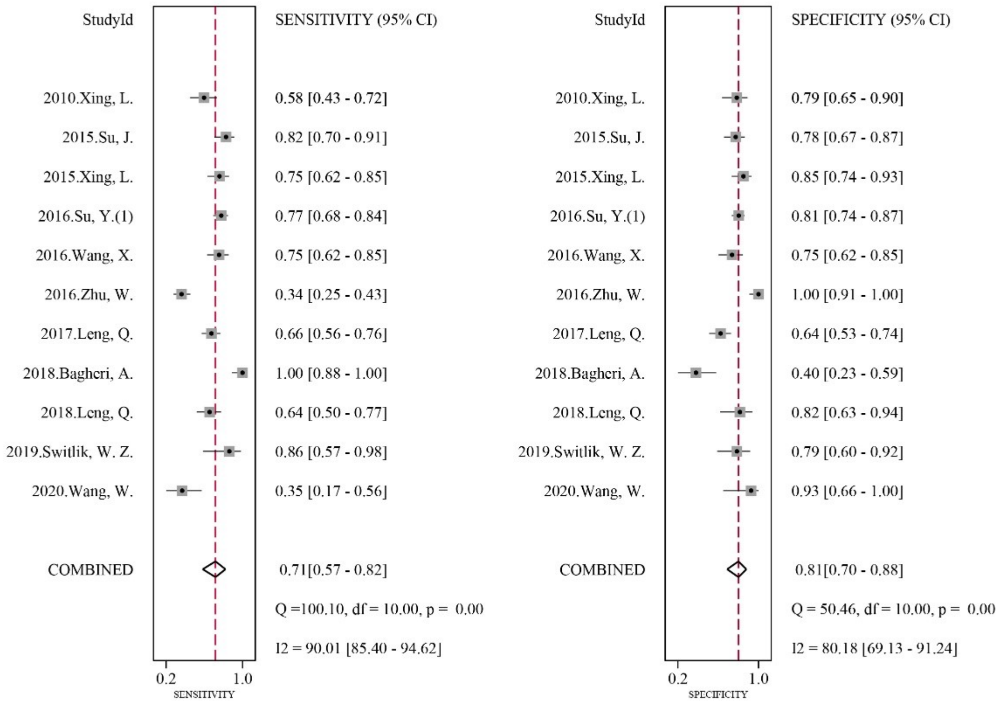

Appendix Figure 5o. Forest plots of the association between miR-210 and the diagnostic value in NSCLC.

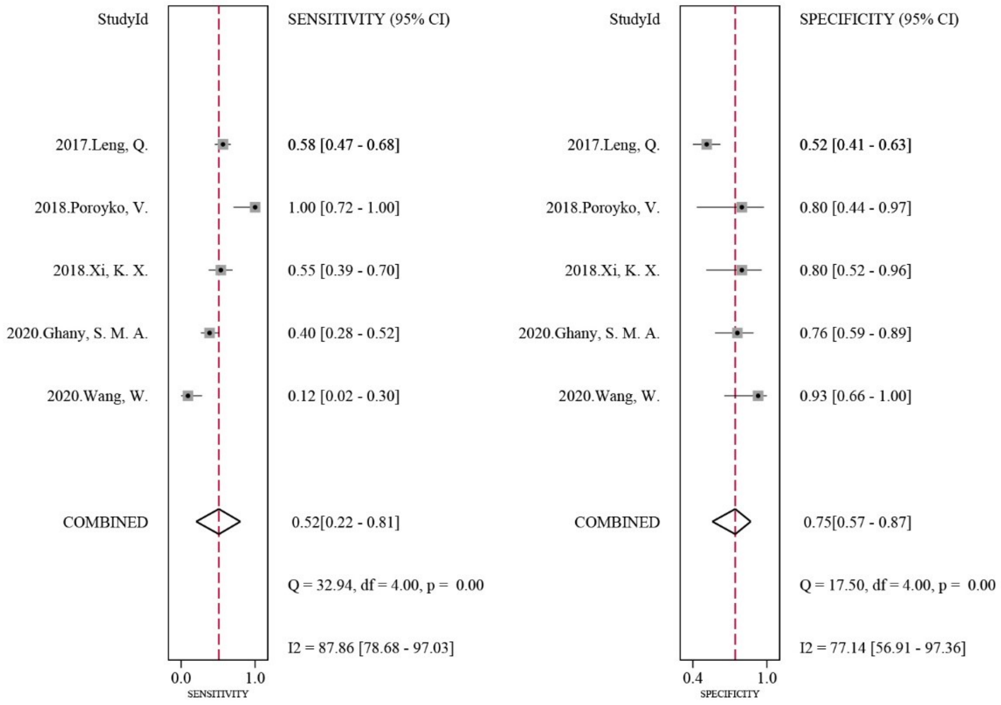

Appendix Figure 5p. Forest plots of the association between miR-221 and the diagnostic value in NSCLC.

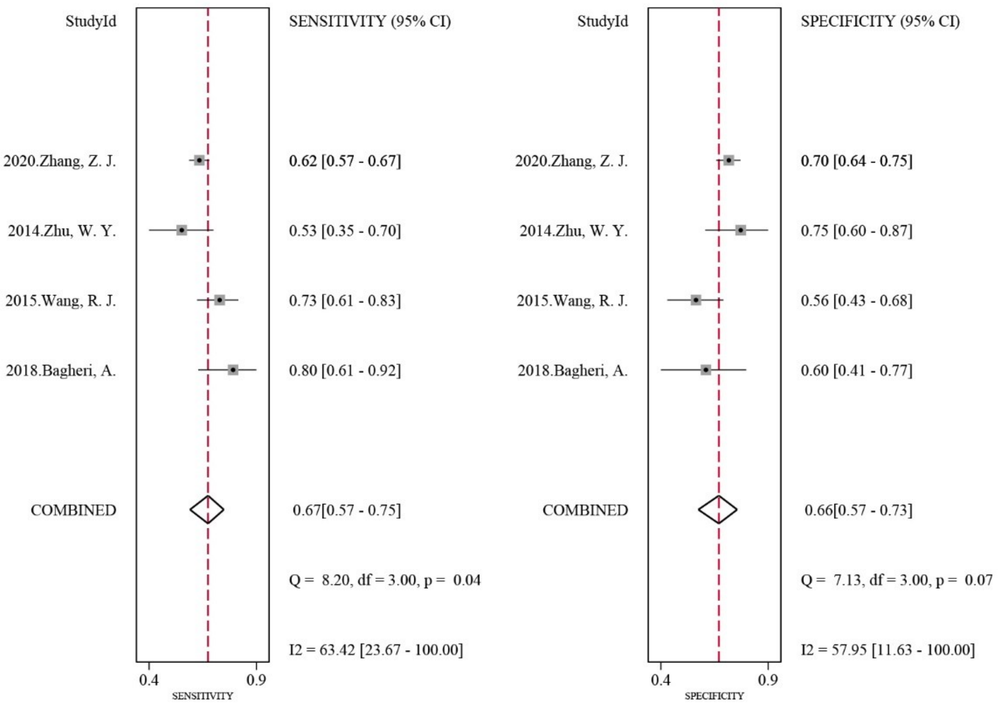

Appendix Figure 5q. Forest plots of the association between miR-125 and the diagnostic value in NSCLC.

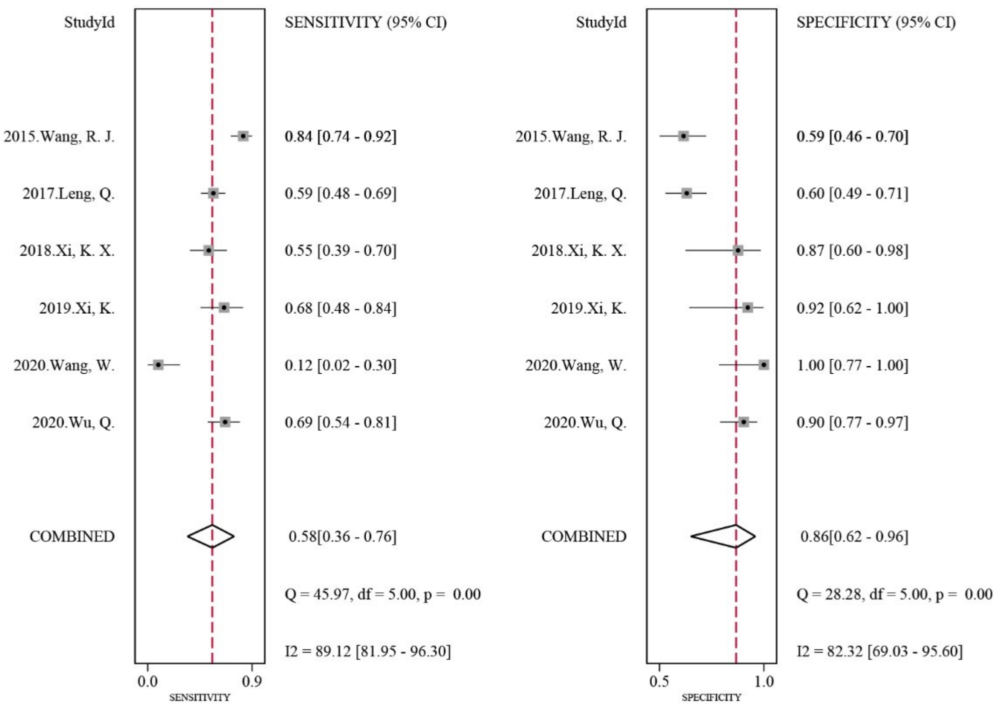

Appendix Figure 5r. Forest plots of the association between miR-146 and the diagnostic value in NSCLC.

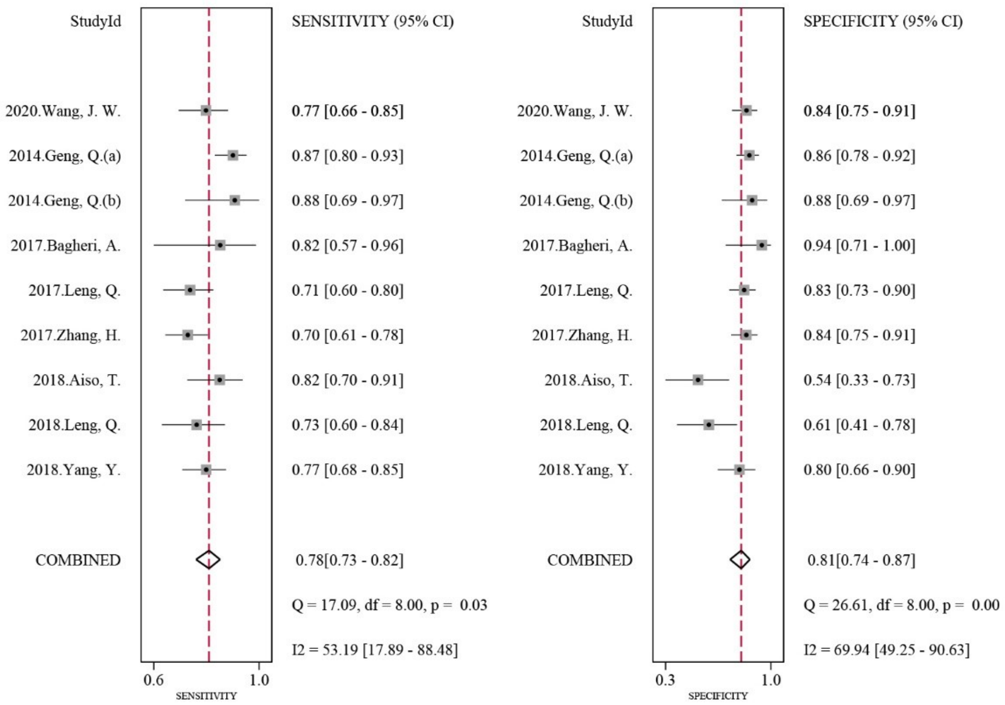

Appendix Figure 5s. Forest plots of the association between miR-223 and the diagnostic value in NSCLC.

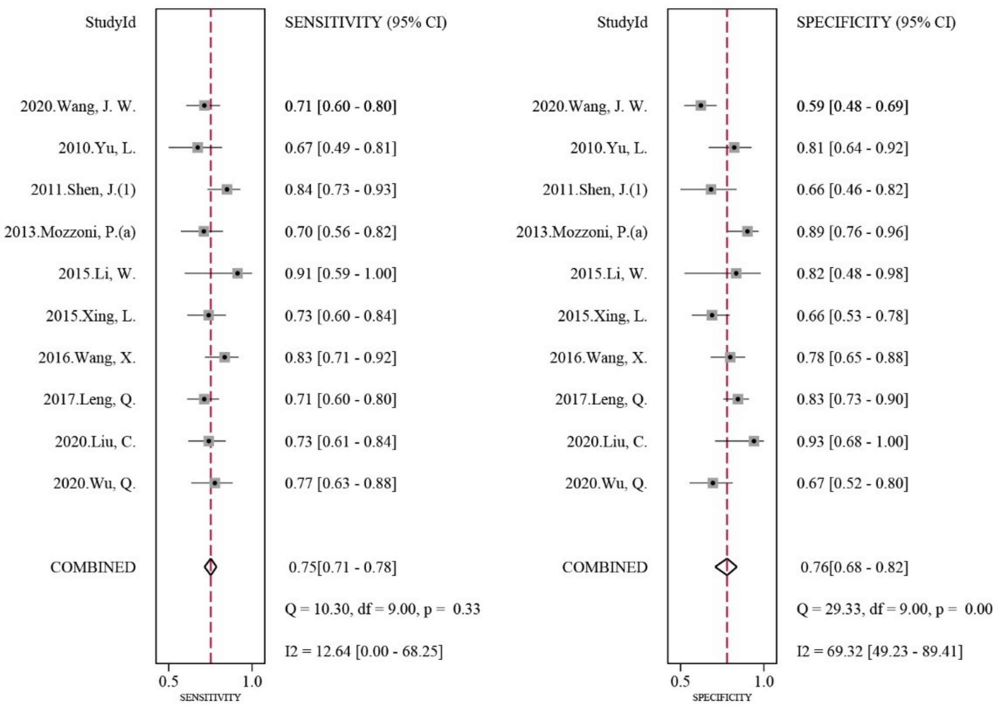

Appendix Figure 5t. Forest plots of the association between miR-486 and the diagnostic value in NSCLC.

Appendix Figure 5. Forest plots of the association between 20 involved single-miRNAs and their diagnostic value in NSCLC.

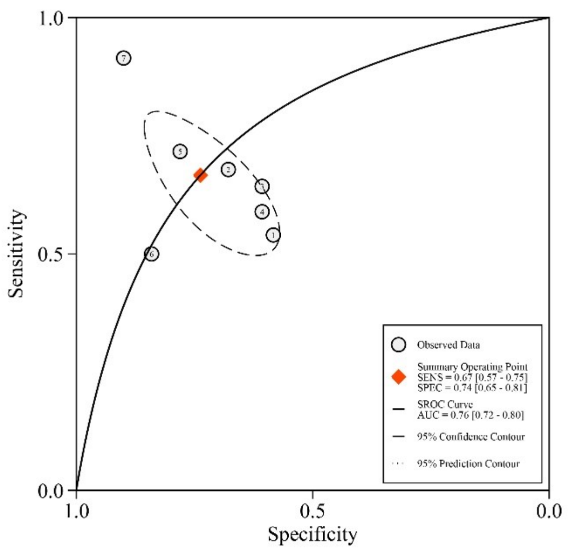

Appendix Figure 6a. The SROC of Let-7 and the diagnostic value in NSCLC.

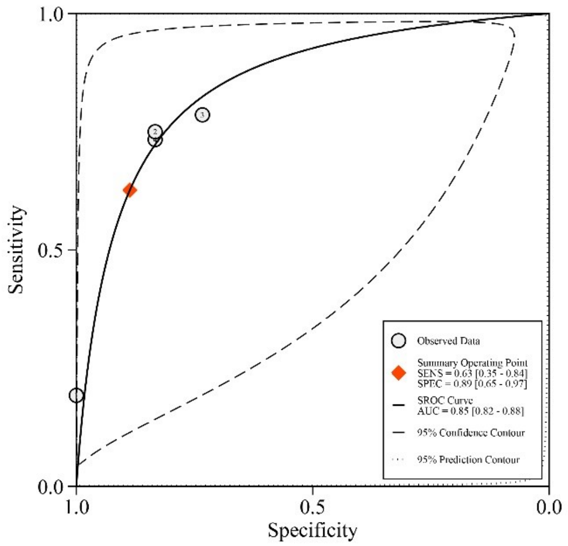

Appendix Figure 6b. The SROC of miR-7 and the diagnostic value in NSCLC.

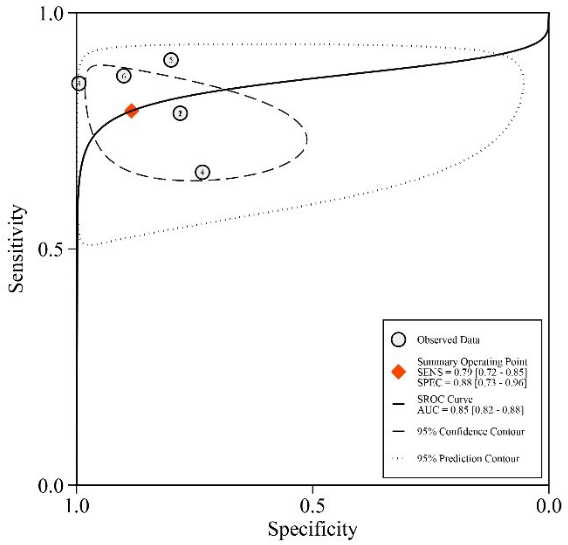

Appendix Figure 6c. The SROC of miR-10 and the diagnostic value in NSCLC.

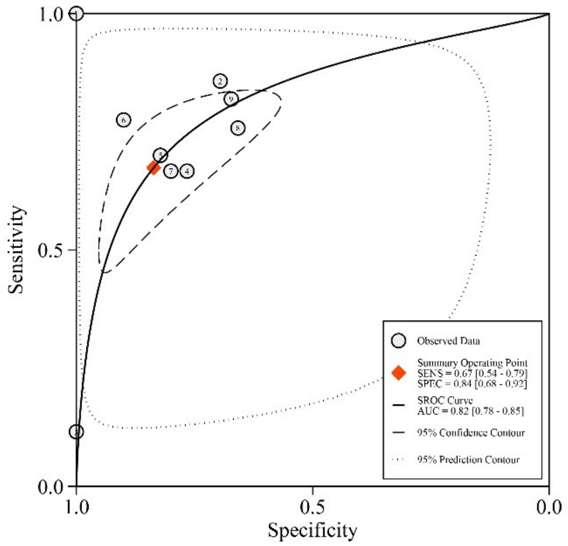

Appendix Figure 6d. The SROC of miR-17 and the diagnostic value in NSCLC.

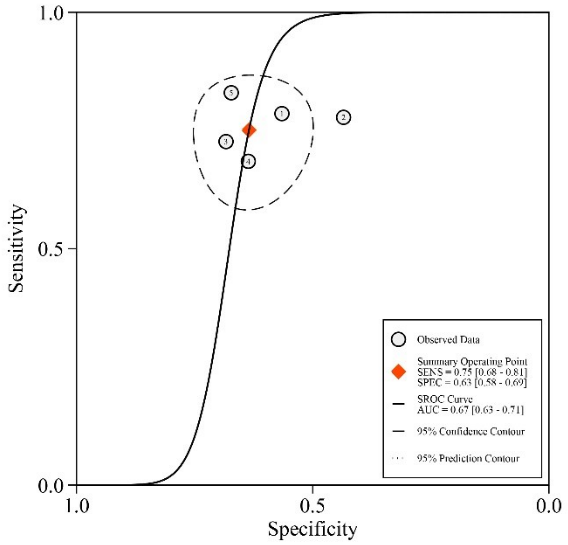

Appendix Figure 6e. The SROC of miR-19 and the diagnostic value in NSCLC.

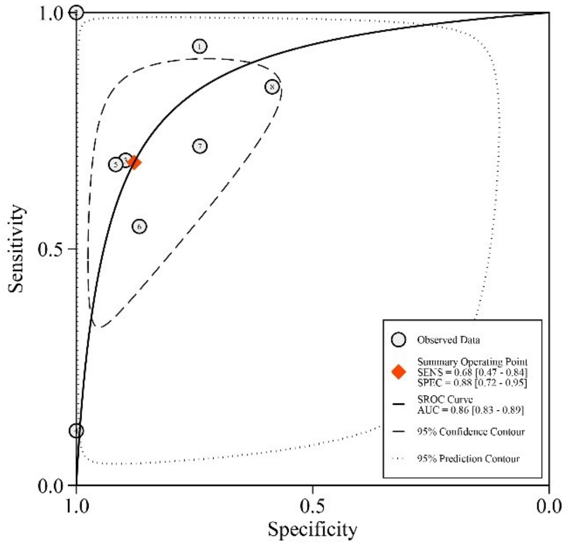

Appendix Figure 6f. The SROC of miR-20 and the diagnostic value in NSCLC.

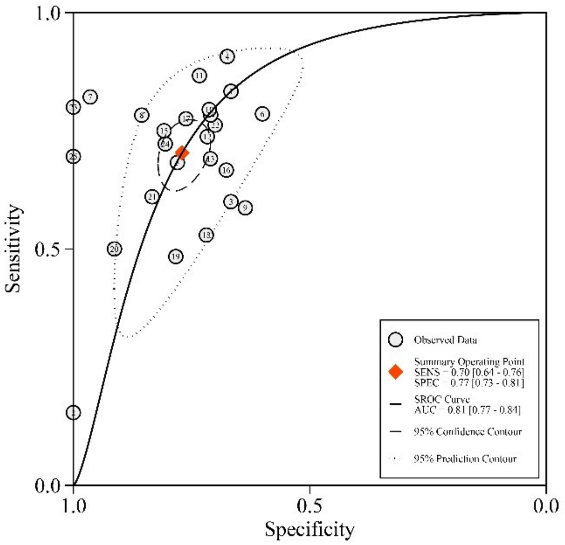

Appendix Figure 6g. The SROC of miR-21 and the diagnostic value in NSCLC.

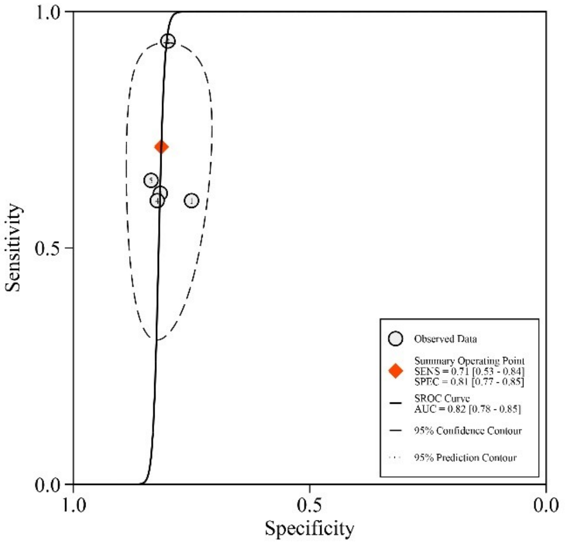

Appendix Figure 6h. The SROC of miR-31 and the diagnostic value in NSCLC.

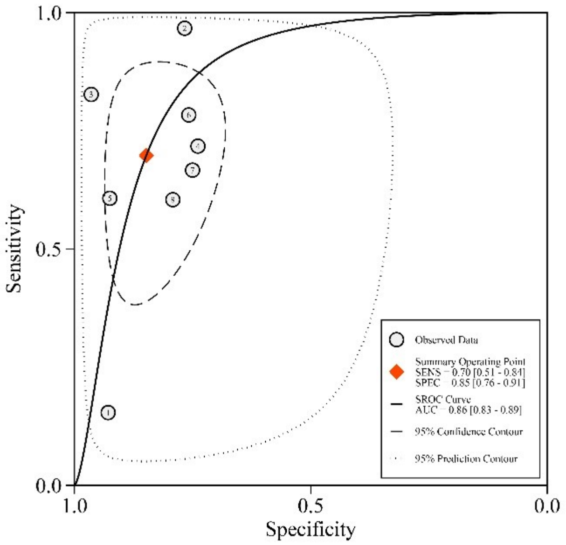

Appendix Figure 6i. The SROC of miR-126 and the diagnostic value in NSCLC.

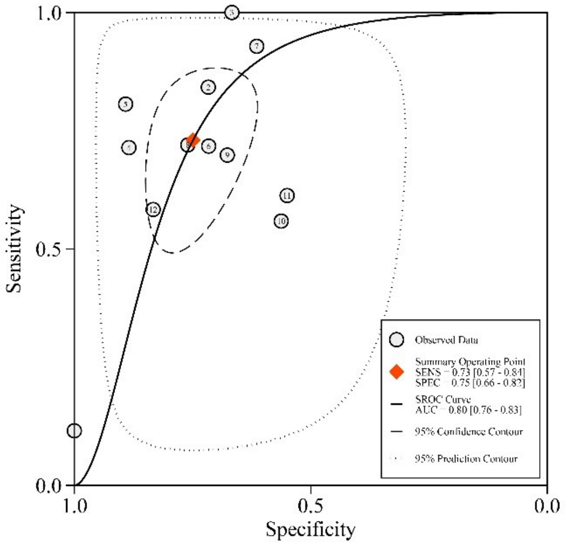

Appendix Figure 6j. The SROC of miR-145 and the diagnostic value in NSCLC.

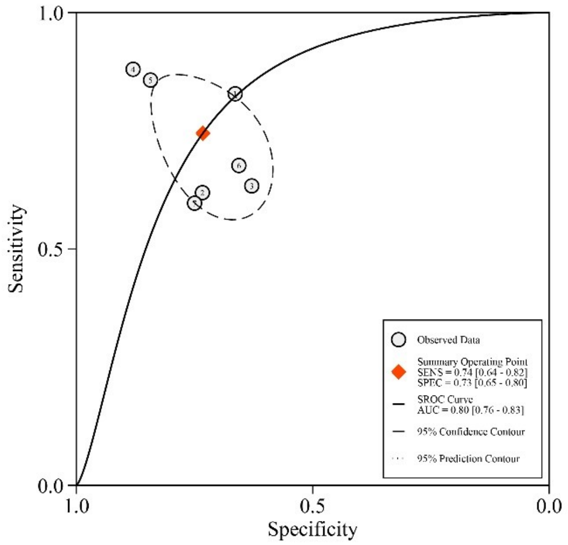

Appendix Figure 6k. The SROC of miR-155 and the diagnostic value in NSCLC.

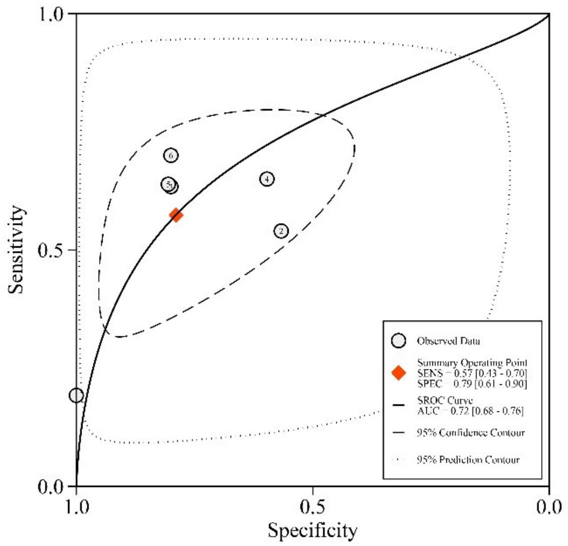

Appendix Figure 6l. The SROC of miR-182 and the diagnostic value in NSCLC.

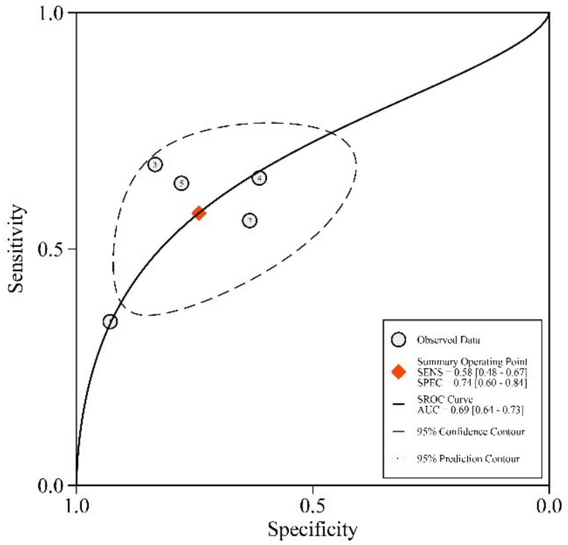

Appendix Figure 6m. The SROC of miR-200 and the diagnostic value in NSCLC.

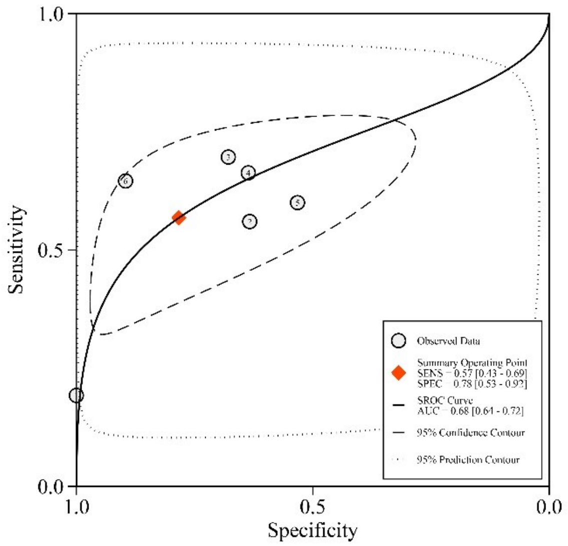

Appendix Figure 6n. The SROC of miR-205 and the diagnostic value in NSCLC.

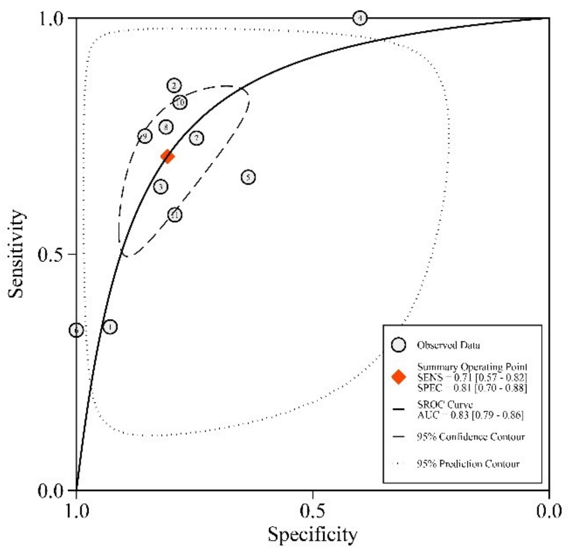

Appendix Figure 6o. The SROC of miR-210 and the diagnostic value in NSCLC.

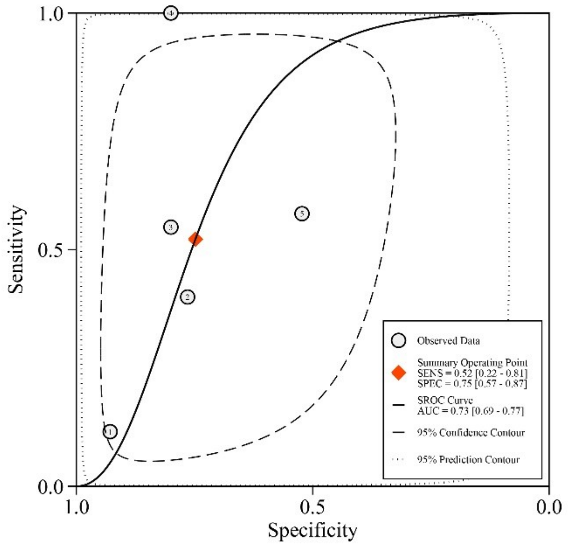

Appendix Figure 6p. The SROC of miR-221 and the diagnostic value in NSCLC.

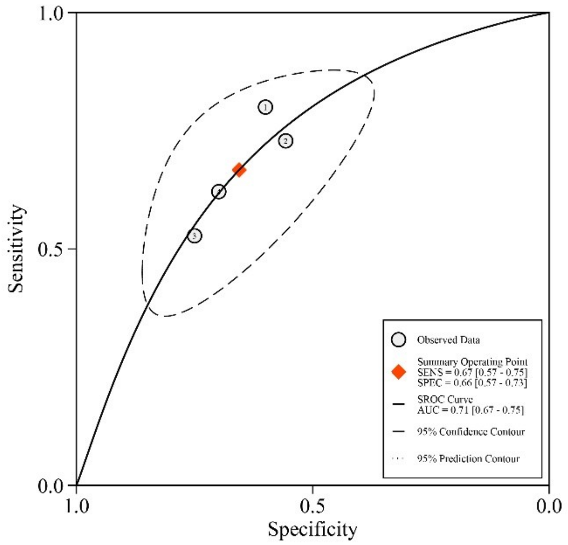

Appendix Figure 6q. The SROC of miR-125 and the diagnostic value in NSCLC.

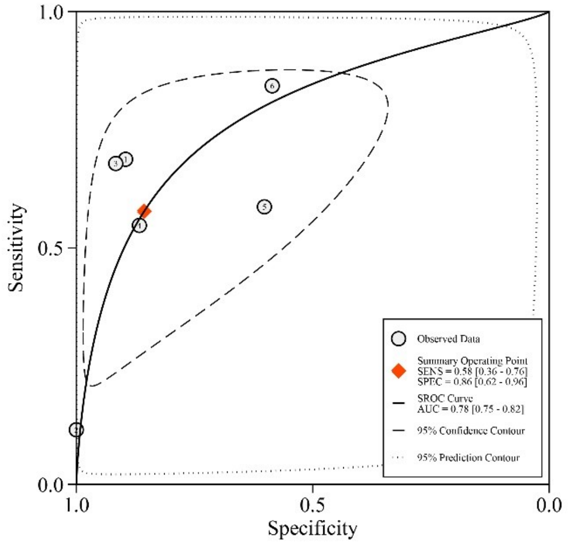

Appendix Figure 6r. The SROC of miR-146 and the diagnostic value in NSCLC.

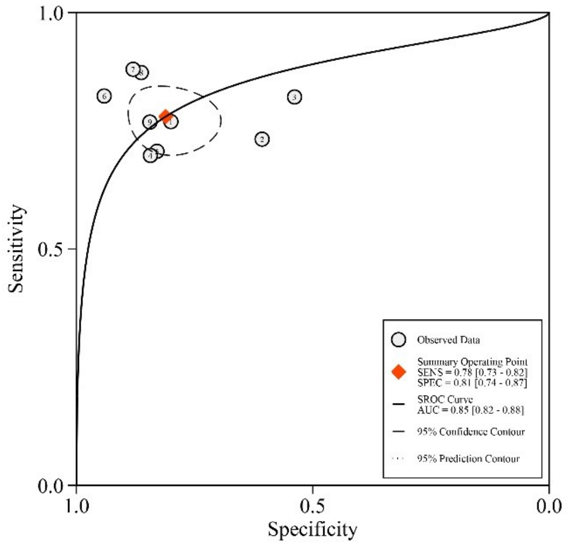

Appendix Figure 6s. The SROC of miR-223 and the diagnostic value in NSCLC.

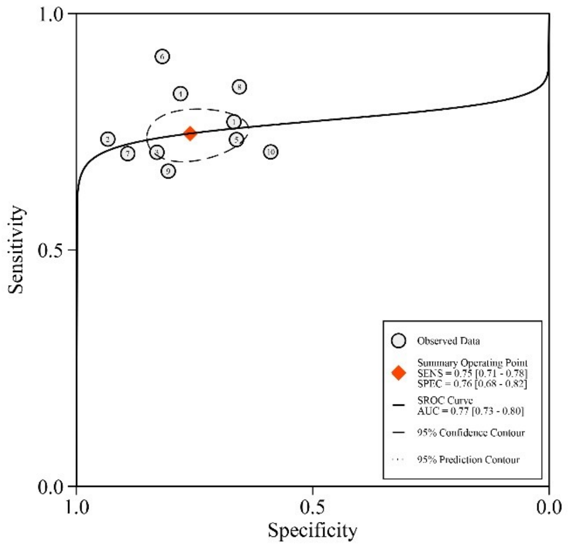

Appendix Figure 6t. The SROC of miR-486 and the diagnostic value in NSCLC.

Appendix Figure 6. The SROC of 20 involved single-miRNAs and their diagnostic value in NSCLC.

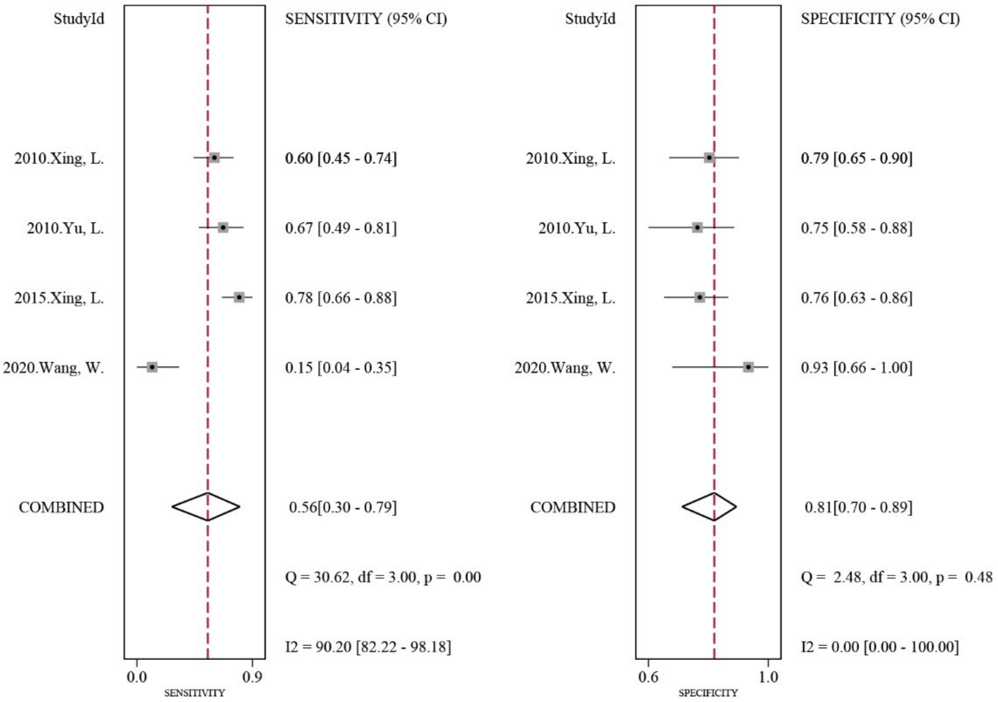

Appendix Figure 7a. Forest plots of the association between miR-126 and the diagnostic value in early-stage NSCLC.

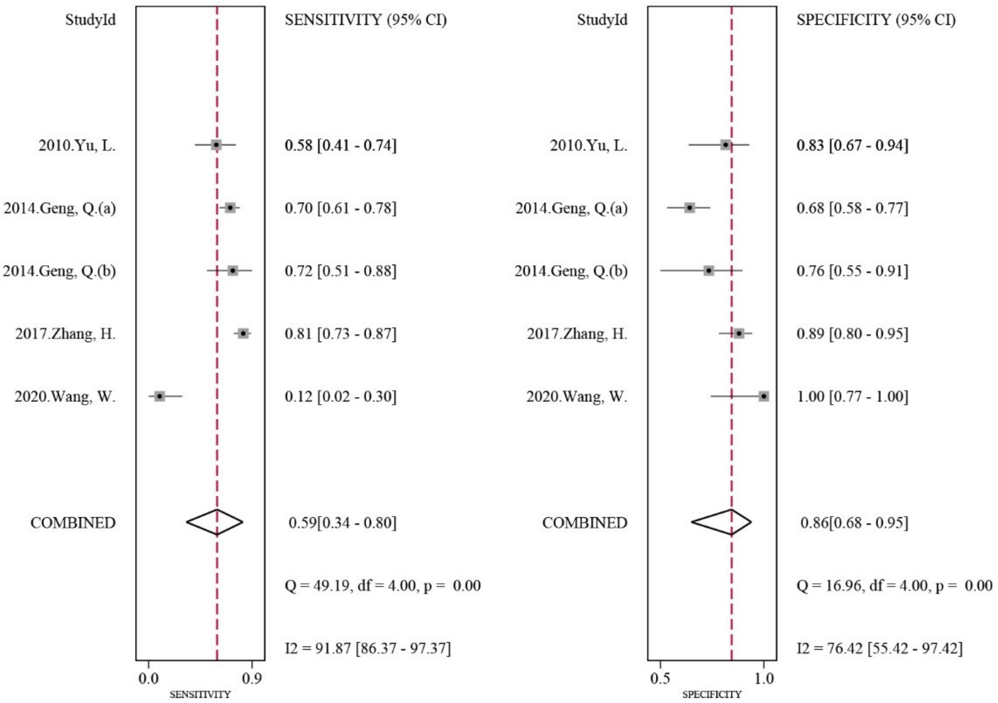

Appendix Figure 7b. Forest plots of the association between miR-145 and the diagnostic value in early-stage NSCLC.

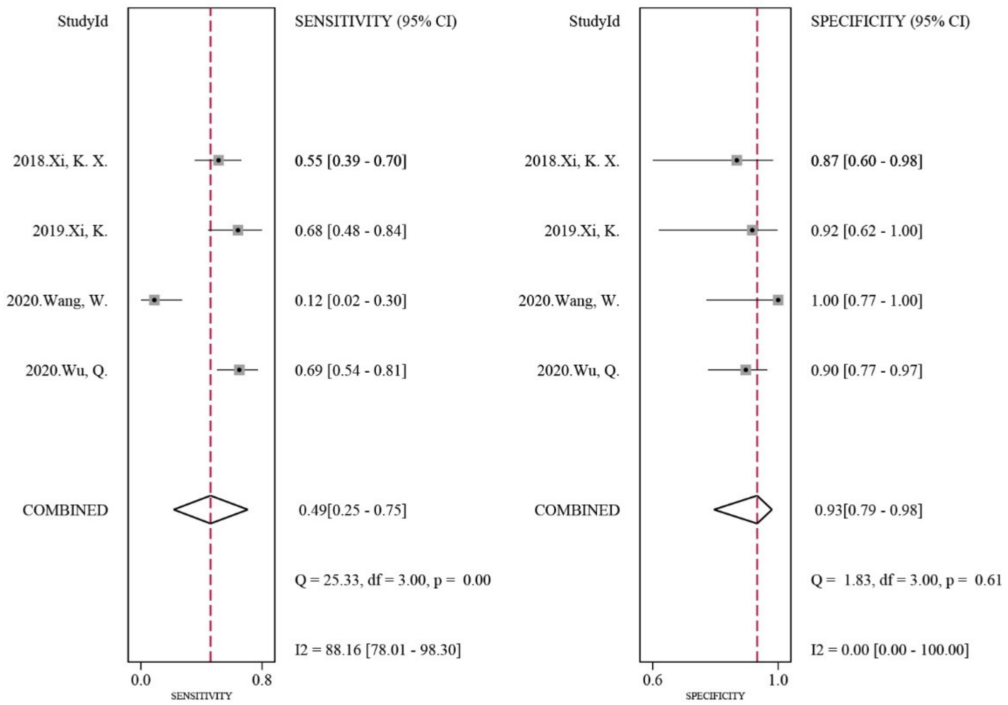

Appendix Figure 7c. Forest plots of the association between miR-146 and the diagnostic value in early-stage NSCLC.

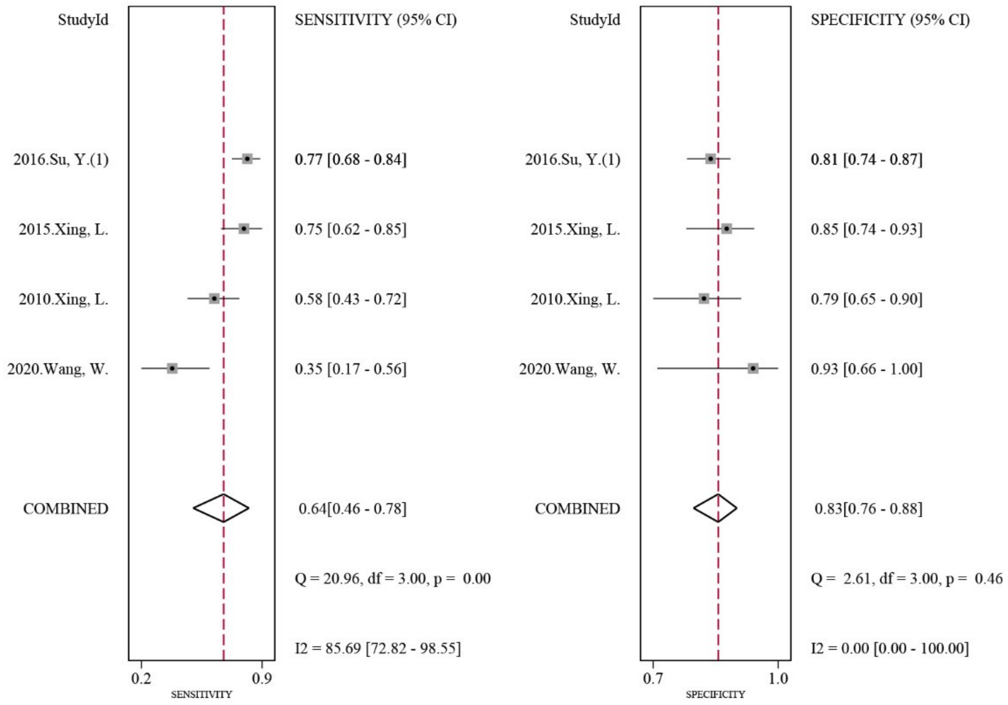

Appendix Figure 7d. Forest plots of the association between miR-210 and the diagnostic value in early-stage NSCLC.

Appendix Figure 7. Forest plots of the association between involved single-miRNAs and their diagnostic value in early-stage NSCLC.

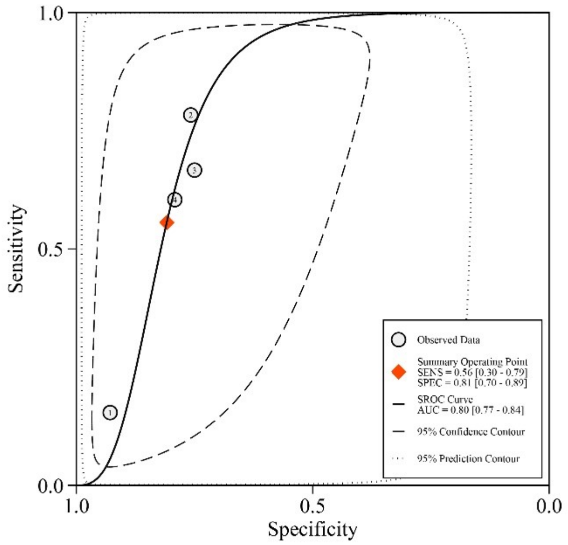

Appendix Figure 8a. The SROC of miR-126 and the diagnostic value in early-stage NSCLC.

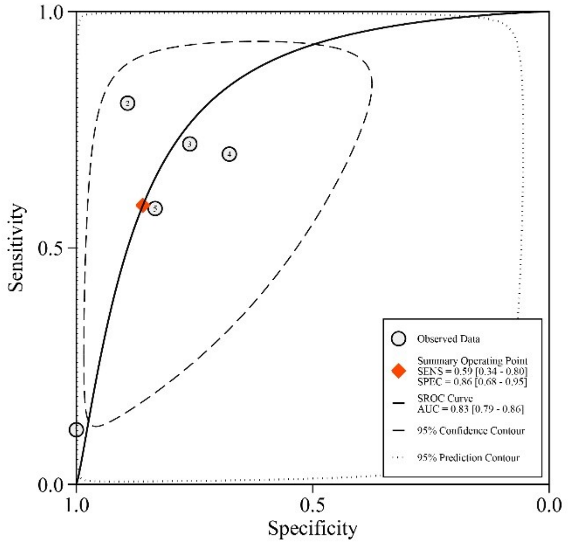

Appendix Figure 8b. The SROC of miR-145 and the diagnostic value in early-stage NSCLC.

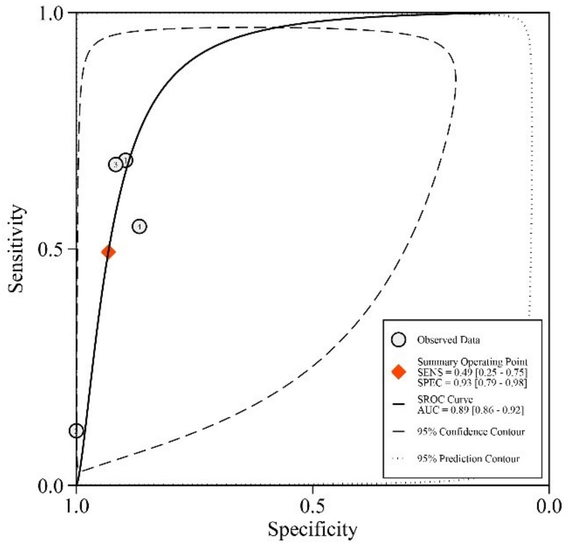

Appendix Figure 8c. The SROC of miR-146 and the diagnostic value in early-stage NSCLC.

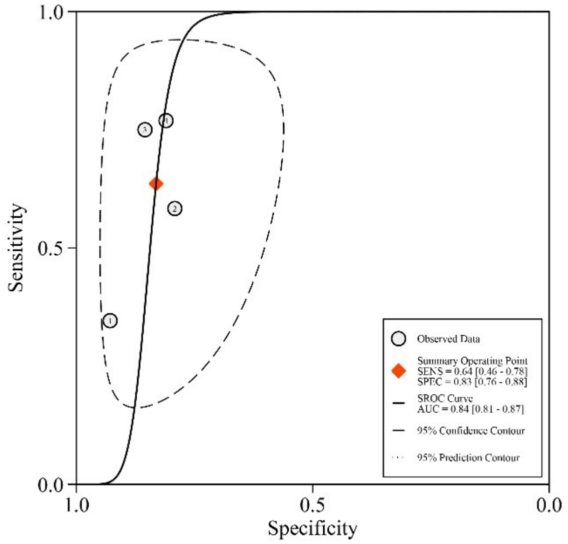

Appendix Figure 8d. The SROC of miR-210 and the diagnostic value in early-stage NSCLC.

Appendix Figure 8. The SROC of single-miRNAs describing their diagnostic value in early-stage NSCLC.

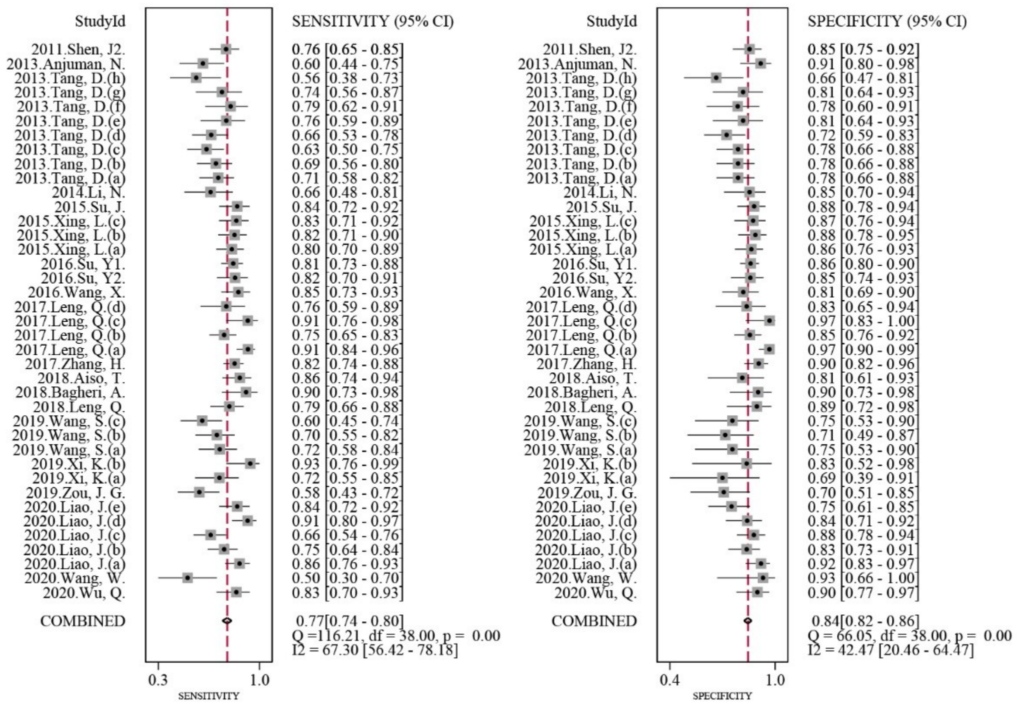

Appendix Figure 9a. Forest plots of Model-1 and the diagnostic value in NSCLC.

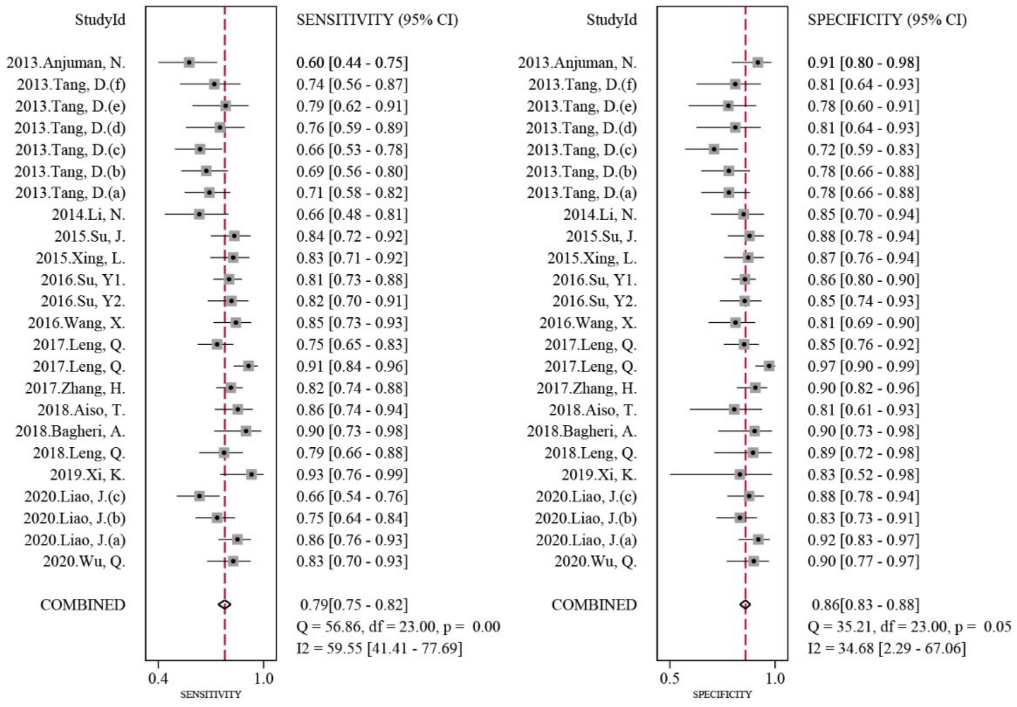

Appendix Figure 9b. Forest plots of Model-2 and the diagnostic value in NSCLC.

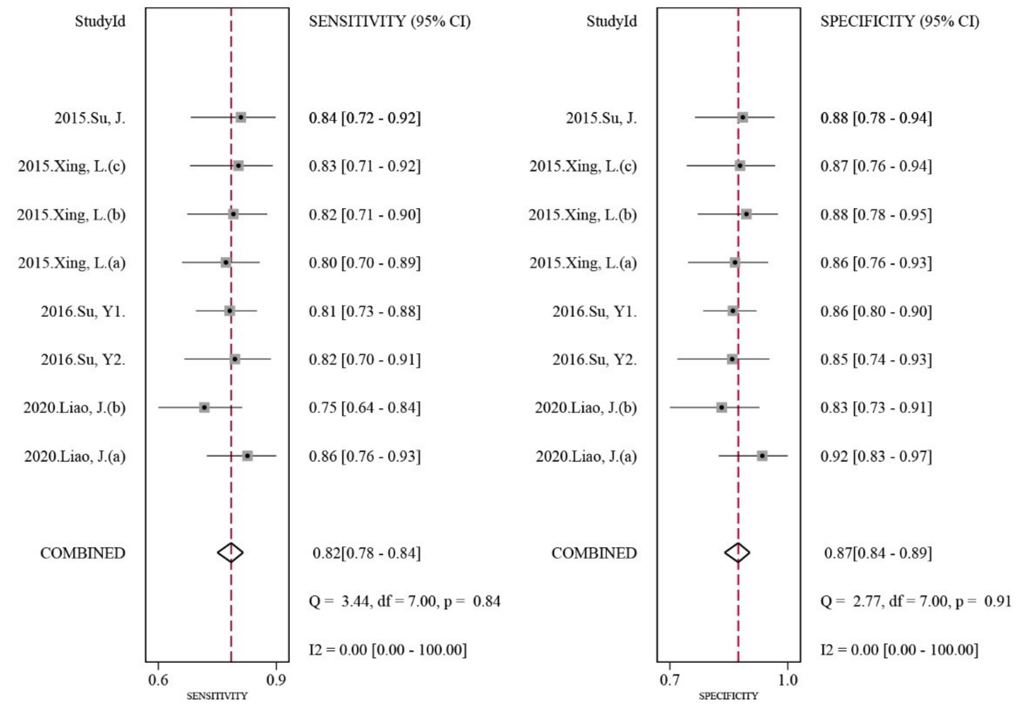

Appendix Figure 9c. Forest plots of Panel-1 and the diagnostic value in NSCLC.

Appendix Figure 9. Forest plots of miRNA panel1 and two models and their diagnostic value in NSCLC.

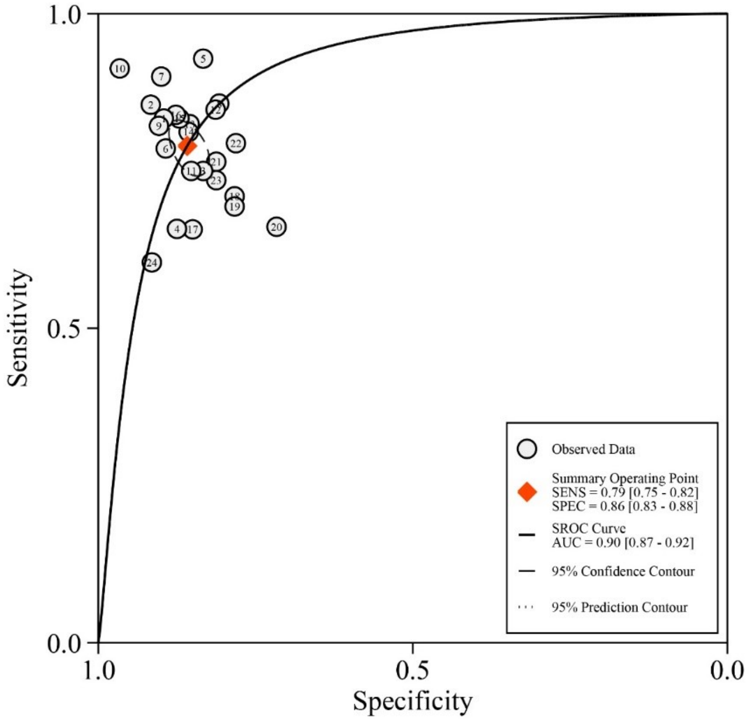

Appendix Figure 10a. The SROC of Model-1 describing the diagnostic value in NSCLC.

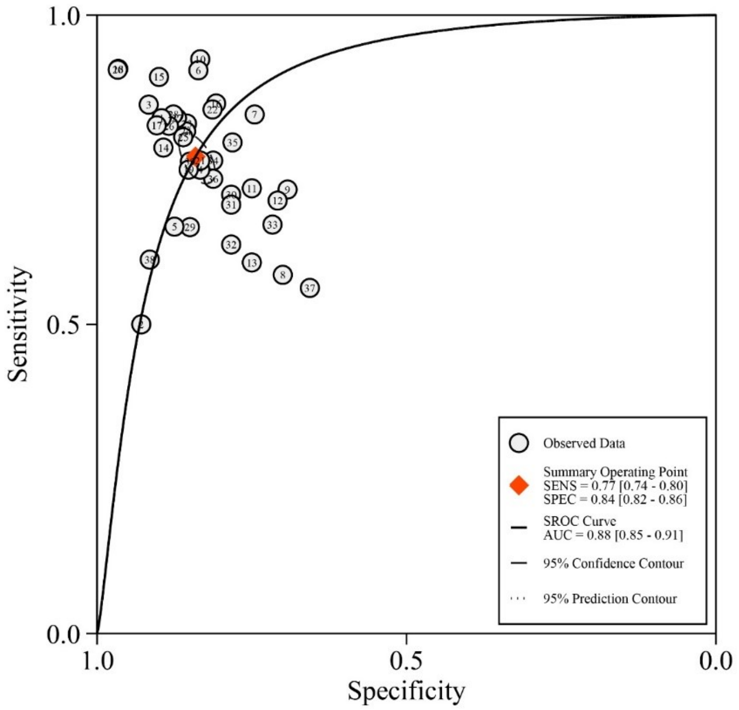

Appendix Figure 10b. The SROC of Model-2 describing the diagnostic value in NSCLC.

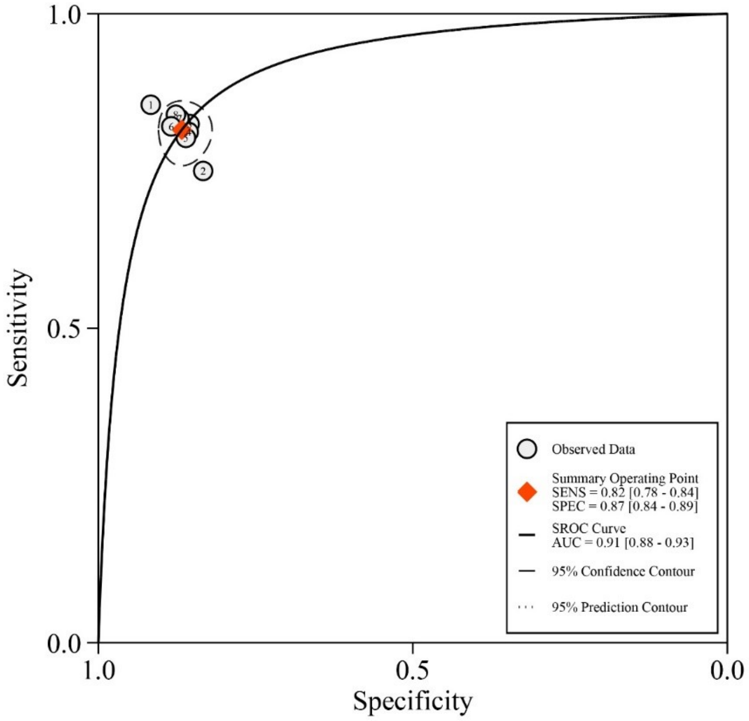

Appendix Figure 10c. The SROC of Panel-1 describing the diagnostic value in NSCLC.

Appendix Figure 10. The SROC of miRNA panel1 and two models describing their diagnostic value in NSCLC.
